# VDAC1: from structure to cancer therapy

**DOI:** 10.3389/fonc.2012.00164

**Published:** 2012-11-29

**Authors:** Varda Shoshan-Barmatz, Dario Mizrachi

**Affiliations:** ^1^Department of Life Sciences, Ben-Gurion University of the NegevBeer-Sheva, Israel; ^2^The National Institute for Biotechnology in the Negev, Ben-Gurion University of the NegevBeer-Sheva, Israel

**Keywords:** apoptosis, mitochondrial porin, cancer metabolism, VDAC protein, hexokinase, anti-apoptotic Bcl-2

## Abstract

Here, we review current evidence pointing to the function of VDAC1 in cell life and death, and highlight these functions in relation to cancer. Found at the outer mitochondrial membrane, VDAC1 assumes a crucial position in the cell, controlling the metabolic cross-talk between mitochondria and the rest of the cell. Moreover, its location at the boundary between the mitochondria and the cytosol enables VDAC1 to interact with proteins that mediate and regulate the integration of mitochondrial functions with other cellular activities. As a metabolite transporter, VDAC1 contributes to the metabolic phenotype of cancer cells. This is reflected by VDAC1 over-expression in many cancer types, and by inhibition of tumor development upon silencing VDAC1 expression. Along with regulating cellular energy production and metabolism, VDAC1 is also a key protein in mitochondria-mediated apoptosis, participating in the release of apoptotic proteins and interacting with anti-apoptotic proteins. The involvement of VDAC1 in the release of apoptotic proteins located in the inter-membranal space is discussed, as is VDAC1 oligomerization as an important step in apoptosis induction. VDAC also serves as an anchor point for mitochondria-interacting proteins, some of which are also highly expressed in many cancers, such as hexokinase (HK), Bcl2, and Bcl-xL. By binding to VDAC, HK provides both metabolic benefit and apoptosis-suppressive capacity that offers the cell a proliferative advantage and increases its resistance to chemotherapy. VDAC1-based peptides that bind specifically to HK, Bcl2, or Bcl-xL abolished the cell’s abilities to bypass the apoptotic pathway. Moreover, these peptides promote cell death in a panel of genetically characterized cell lines derived from different human cancers. These and other functions point to VDAC1 as a rational target for the development of a new generation of therapeutics.

## OVERVIEW

Research over the past decade has extended the prevailing view of the mitochondrion to include functions well beyond the critical bioenergetics role of supplying ATP to include cell signaling events, inter-organellar communication, aging, cell proliferation, a target in disease, and apoptosis. Mitochondria thus play a central role in the regulation of apoptosis and serve as the venue for cellular decisions leading to cell life or death. One of the mitochondrial proteins controlling cell life and death is the voltage-dependent anion channel (VDAC), also known as mitochondrial porin. VDAC, located in the mitochondrial outer membrane, functions as gatekeeper for the entry and exit of mitochondrial metabolites, thereby controlling cross-talk between mitochondria and the rest of the cell. VDAC is also a key player in mitochondria-mediated apoptosis. Thus, in addition to regulating the metabolic and energetic functions of mitochondria, VDAC appears to act as a convergence point for a variety of cell survival and cell death signals, mediated by its association with various ligands and proteins. The focus of this review will be on the central role of VDAC in cell life and death, addressing VDAC function in the regulation of mitochondria-mediated apoptosis, with an emphasis on structure–function relationships. Understanding VDAC structure–function relationships is critical for deciphering how this channel can perform such a variety of functions, all important for cell life and death. Finally, this review will also provide insight into VDAC function in Ca^2+^ homeostasis, protection against oxidative stress, regulation of apoptosis, and involvement in several diseases, as well as its role in the action of different drugs.

## VDAC: ISOFORMS, LOCATION, AND TRANSPORT ACTIVITY

### VDAC ISOFORMS

In mammals, three homologous genes encode three VDAC isoforms, namely VDAC1, VDAC2, and VDAC3 (Shoshan-Barmatz et al., 2010). The three proteins have similar molecular weights (30–35 kDa), each shares approximately 70% identity, and all three can be found in most tissues, albeit in different amounts, with the most abundant sub-type being VDAC1 and the least common form being VDAC3 (Cesar Mde and Wilson, 2004; Yamamoto et al., 2006; De Pinto et al., 2010a; Shoshan-Barmatz et al., 2010; Messina et al., 2012). VDAC1 and VDAC2 but not VDAC3 can form a channel upon reconstitution into an artificial lipid bilayer, with VDAC1 showing voltage-gated high conductance channel properties (see Channel Activity of VDAC1). VDAC2 also presents normal gating activity, while VDAC3 does not insert readily into membranes and generally does not gate well, even at high membrane potentials (up to 80 mV; Sampson et al., 1997; Xu et al., 1999).

The specific role of each VDAC isoform remains unclear, although evidence indicates that they serve different physiologic functions (Sampson et al., 1997; Xu et al., 1999; Wu et al., 1999). For example, the absence of VDAC1 caused multiple defects in respiratory complex activities in both skeletal and cardiac muscle, while in VDAC3-deficient mice, these defects are restricted to the heart (Anflous-Pharayra et al., 2010), suggesting that *in vivo*, these two isoforms fulfill distinct physiologic roles. In mice, *vdac1* and *vdac2* deletion reduces respiratory capacity (Wu et al., 1999), the absence of VDAC3 causes male sterility, and a lack of both VDAC1 and VDAC3 causes inhibited growth (Sampson et al., 2001). Furthermore, it was demonstrated that VDAC1- and VDAC3-lacking mice show deficits in learning behavior and synaptic plasticity (Weeber et al., 2002). VDAC3-lacking mice were male-infertile because their mitochondria and the axoneme of their sperm are structurally altered (Sampson et al., 2001). Finally, *vdac1-* and *vdac3-*deficient mice are viable, whereas embryos with a homozygous deletion of *vdac2* die during development (Cheng et al., 2003).

VDAC1 interacts with different proteins and factors, such as hexokinase (HK; Abu-Hamad et al., 2008) and glyceraldehyde-3-phosphate dehydrogenase (GAPDH; Tarze et al., 2007), while biochemical data indicate that VDAC1 but not VDAC2 binds HK (Blachly-Dyson et al., 1993). This, however, has been questioned (Azoulay-Zohar and Aflalo, 1999). Lately, it was demonstrated that HK-I and VDAC3 exhibit a higher degree of mitochondrial co-localization than does HK-I with either VDAC1 or VDAC2 (Neumann et al., 2010).

Large proteomic surveys and other studies have shown that all three VDAC isoforms are subject to both phosphorylation and acetylation at multiple sites (Distler et al., 2007; Wang et al., 2008; Choudhary et al., 2009; Gauci et al., 2009; Menzel et al., 2009; Kerner et al., 2012). Analysis of the amino acid sequence of VDAC1 showed that the first methionine is deleted, while the second amino acid, an alanine, is acetylated (Kayser et al., 1989; Gauci et al., 2009). Among the other post-translation modifications VDAC1 undergoes are phosphorylation of serine, threonine, and tyrosine residues (Distler et al., 2007; Kerner et al., 2012) and acetylation of lysines (Kim et al., 2006; Schwer et al., 2009; Zhao et al., 2010; Yang et al., 2011). Recently glycogen synthase kinase 3 (GSK3)-mediated VDAC phosphorylation was reported, allowing for control of outer mitochondrial membrane (OMM) permeabilization in hepatosteatosis (Martel et al., 2012). Currently, the effects of these modifications on VDAC activity are not clear.

### VDAC LOCATION AND METABOLITE TRANSPORT

**FIGURE 1:**
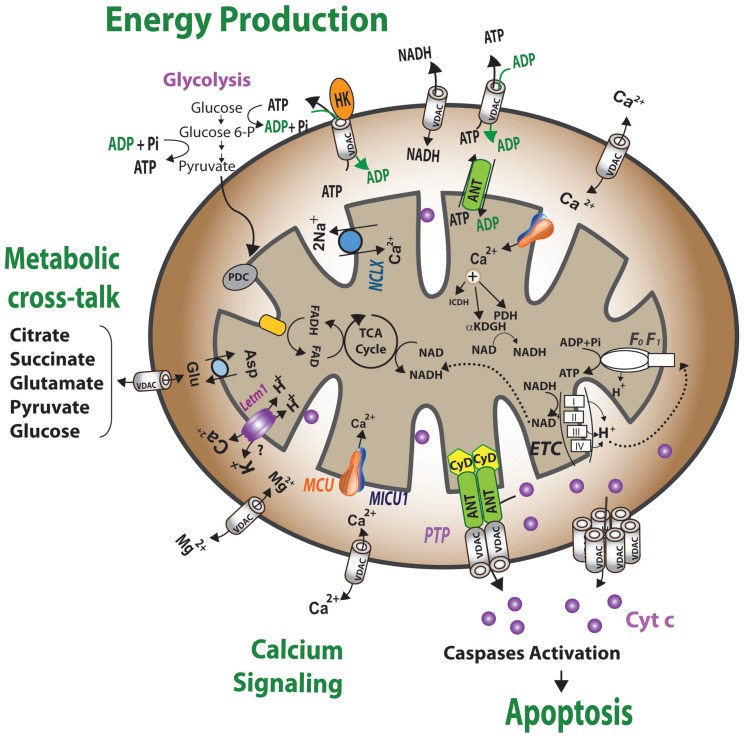
**Schematic representation of VDAC1 as a multi-functional channel and convergence point for a variety of cell survival and cell death signals.** The various functions of VDAC1 include control of the metabolic cross-talk between the mitochondria and the rest of the cell, cellular energy homeostasis by transporting ATP/ADP and NADH between the inter-membrane space and the cytosol and by binding HK, signaling by transporting Ca^2+^, ROS release to the cytosol and apoptosis, both by binding to the apoptosis regulatory proteins, Bcl-2 family and HK. Also presented are the Ca^2+^ influx and efflux transport systems in the outer and inner mitochondrial membranes and Ca^2+^-mediated regulation of the tricarboxylic acid (TCA) cycle. The activation of pyruvate dehydrogenase (PDH), isocitrate dehydrogenase (ICDH) and α-ketoglutarate dehydrogenase (αKGDH) by intra-mitochondrial Ca^2+^, leading to enhanced activity of the TCA cycle, is shown. The electron transport chain (ETC) and the ATP synthase (F_o_F_1_) are also presented. VDAC in the OMM is presented as a Ca^2+^ channel. In the IMM, the uptake of Ca^2+^ into the matrix is mediated by a Ca^2+^-selective transporter, the mitochondrial Ca^2+^ uniporter (MCU), regulated by a calcium-sensing accessory subunit (MICU1). Ca^2+^ efflux is mediated by NCLX, a Na^+^/Ca^2+^ exchanger. High levels of matrix Ca^2+^ accumulation trigger the opening of the PTP, a fast Ca^2+^ release channel. Molecular fluxes are indicated by arrows.

VDAC is localized to the OMM of all eukaryotes (Benz, 1994), where it assumes a crucial position in the cell, serving as the main interface between mitochondrial and cellular metabolisms. VDAC is permeable to uncharged molecules up to ∼5,000 Da in the open configuration, mediating the flux of ions, nucleotides and other metabolites across the OMM (Shoshan-Barmatz et al., 2010; **Figure 1**). In keeping with its two-way trafficking role, VDAC1 enables substrates, including pyruvate, malate, succinate, and NADH, to enter the mitochondria and mediates the exit of newly formed molecules, such as hemes (Shoshan-Barmatz et al., 2010). Hence, down-regulation of VDAC1 expression results in reduced metabolite exchange between mitochondria and the cytosol, making VDAC1 essential for energy production and cell growth (Abu-Hamad et al., 2006). Similarly, alterations in mitochondrial function are linked to VDAC closure, which limits the normal flow of metabolites in and out of mitochondria (Vander Heiden et al., 2000; Holmuhamedov and Lemasters, 2009). VDAC1, at the OMM, is also involved in the entry and exit of Ca^2+^ (see VDAC1 Transport of Ca^2+^ and Function in ER-mitochondria Cross-talk). VDAC, furthermore, functions in cholesterol transport across the OMM (Rone et al., 2009). Indeed, VDAC has been proposed to be a necessary component of a protein complex involved in mitochondrial membrane cholesterol distribution and transport and to play an important role in altered cholesterol synthesis and transport in Morris hepatoma cells (Campbell and Chan, 2008).

Given its location at the boundary between the mitochondria and the cytosol, VDAC1 is able to interact with proteins that mediate and regulate the integration of mitochondrial functions with other cellular activities. VDAC1 interacts with HK and creatine kinase to convert newly generated ATP into high-energy storage forms, like glucose-6-phosphate (G-6-P) and creatine phosphate, in brain and muscle, respectively. The over-expression of VDAC1 in some cancer cells may be related to its multi-functional activities, as required by high energy-demanding cells (Shoshan-Barmatz and Golan, 2012; see VDAC Expression Levels in Cancers and Enhancement by Pro-apoptotic Drugs).

Thus, VDAC is a dynamic regulator of global mitochondrial function both in health and disease (Lemasters and Holmuhamedov, 2006).

### EXTRA-MITOCHONDRIAL LOCALIZATION OF VDAC

VDAC1 was once thought to be localized solely to the OMM (Yu and Forte, 1996). Indeed, although VDAC is present in abundance in the OMM, various studies have revealed that VDAC is also localized to cell compartments other than mitochondria (Bathori et al., 2000; Shoshan-Barmatz and Israelson, 2005; De Pinto et al., 2010b). VDAC, first isolated from human plasma membrane lymphocytes (Thinnes et al., 1989), was subsequently detected in other cells of various tissues (Bathori et al., 2000; De Pinto et al., 2010b). A splice variant of the mitochondrial VDAC1 gene encoding a leader peptide of 13 amino acids at the NH_2_ terminus was detected and termed plasmalemmal VDAC1 (pl-VDAC1; Buettner et al., 2000). The protein from this splice variant exhibited extra-mitochondrial trafficking to the endoplasmic reticulum (ER), the Golgi apparatus, and the plasma membrane (Gonzalez-Gronow et al., 2003; De Pinto et al., 2010b). Caveolae and caveolae-like domains were shown to contain VDAC (Bathori et al., 1999). The extra-mitochondrial localization of VDAC in bovine outer dense fibers, a cytoskeletal component of the sperm flagellum, has been reported (Hinsch et al., 2004; Triphan et al., 2008; Cassara et al., 2010). VDAC2 was shown to be present in the membrane components of human spermatozoa (Liu et al., 2011). The presence of VDAC in the sarcoplasmic reticulum (SR) of amphibian and mammalian skeletal muscle and in the ER of rat cerebellum has also been demonstrated (Shoshan-Barmatz et al., 1996, 2004; Shoshan-Barmatz and Israelson, 2005).

The exact functions of the extra-mitochondrial VDAC are as yet unknown. pl-VDAC1 was suggested to facilitate regulatory volume decrease in epithelial cells and to play a role in cellular ATP release and volume control (Okada et al., 2004). The possible functions of VDAC in the SR/ER include providing a pathway for transport of Ca^2+^, nucleotides and other metabolites across the membrane, and involvement in apoptosis. The participation of VDAC in supra-molecular complexes and intracellular communication, including calcium signal delivery between the ER and mitochondria, has also been postulated (Shoshan-Barmatz and Israelson, 2005; see VDAC1 Transport of Ca^2+^ and Function in ER-mitochondria Cross-talk).

## CHANNEL ACTIVITY OF VDAC1

### VDAC CONDUCTANCE, ION SELECTIVITY, AND VOLTAGE-DEPENDENT CHANNEL GATING

**FIGURE 2:**
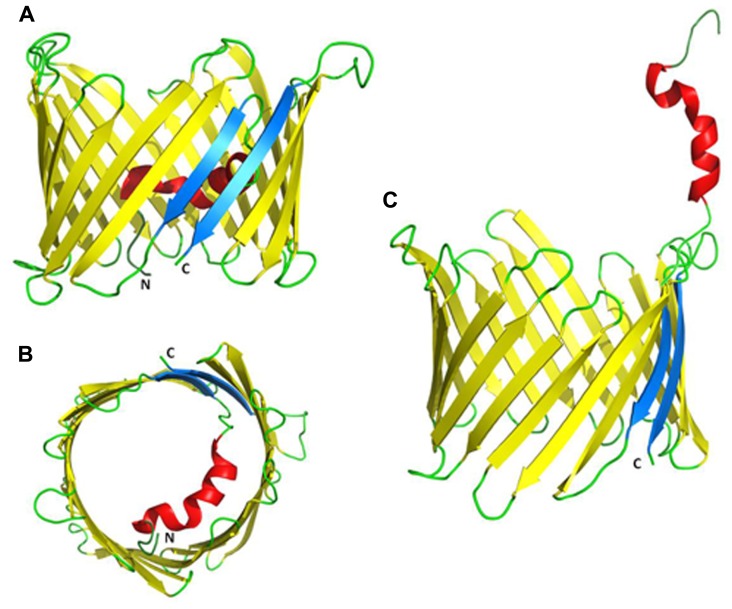
**Proposed three-dimensional structure of VDAC1. (A)** Side-view of the X-ray crystal structure of mouse VDAC1 (Ujwal et al., 2008) in a ribbon representation. The β-barrel is formed by 19 β-strands and the N-terminal helix is folded into the pore interior. β-strands 1 and 19 are parallel and colored blue. The C- and N-termini are annotated as C and N, respectively. Loops and unstructured regions are colored green. **(B)** Top-view of VDAC1 with the N-terminal inside the pore. **(C)** VDAC1 in a proposed conformation with the N-terminal outside the channel where it can interact with other proteins (PDB code: 3EMN).

The channel properties of VDAC1 have been examined following reconstitution of the purified protein into a planar lipid bilayer (PLB). VDAC1 has been purified using various procedures and detergents (Shoshan-Barmatz et al., 2010). Bilayer-reconstituted VDAC1 assumes multiple voltage-dependent conformational states, displaying different selectivities and permeabilities. VDAC1 shows symmetrical bell-shaped voltage-dependent conductance with the highest conductance (4 nS at 1 M KCl) at low potentials of -20 to +20 mV (Colombini, 1989; Benz, 1994; **Figure 2**). At low potentials, when in the fully open state, VDAC1 selectively conducts small ions (e.g., Cl^–^, K^+^, Na^+^), yet shows a preference for anions, such as phosphate, chloride, adenine nucleotides, glutamate, and other anionic metabolites, and large cations, such as acetylcholine, dopamine, and Tris (Shoshan-Barmatz et al., 2010). At higher positive or negative potentials (>30–60 mV), the channel conductance is reduced and the selectivity shifts to small cations. In this scenario, the channel becomes virtually impermeable to ATP and ADP (Colombini, 1989; Benz, 1994; Shoshan-Barmatz et al., 2010).

As a voltage-gated channel, VDAC1 must possess a voltage sensor to respond to changes in transmembrane voltage. It is believed that VDAC1 channels rely on two separate gating processes, one that occurs at positive transmembrane potentials and the other at negative potentials (Song et al., 1998). The N-terminal α-helical segment of the channel has been proposed to act as the voltage sensor, gating the pore via conformational changes and/or movements (see The N-terminal Region of VDAC1: Location, Translocation and Channel Gating). Clearly, the molecular nature of VDAC1 gating mechanism has not yet been resolved.

### VDAC CHANNEL MODULATORS AND INHIBITORS

Despite the critical involvement of VDAC1 in various mitochondrial functions, little is known of how VDAC is regulated. Accumulated evidence suggests that VDAC1 function is modulated by various physiological ligands, such as glutamate, adenine nucleotides, NADH, and non-physiological compounds, such as Koenig’s polyanion, ruthenium red (RuR), dicyclohexylcarbodiimide (DCCD), and 4,4′-diisothiocyanatostilbene-2,2′-disulfonic acid (DIDS; Shoshan-Barmatz et al., 2006).

NADH was found to regulate the gating of mammalian, fungal, and plant VDAC (Lee et al., 1994). For mammalian VDAC1, molecular and biochemical evidence indicates that the protein possesses one or more nucleotide-binding site(s) (Rostovtseva et al., 2002; Yehezkel et al., 2006, 2007). Glutamate specifically, but not aspartate or GABA, eliminates the bell shape of VDAC voltage-dependence channel conductance (Gincel et al., 2000). Accumulating evidence also suggests that VDAC possesses regulatory binding sites for Ca^2+^ (reviewed in Shoshan-Barmatz and Gincel, 2003; Shoshan-Barmatz et al., 2006).

Various compounds targeting apoptotic cell death or cell proliferation were found to mediate their activity via interaction with VDAC1, modulating VDAC1 activity (see VDAC as a Pharmacological Target for Compounds Affecting Cell Proliferation or Apoptosis). In addition, VDAC1, acting as a docking site for various proteins, is regulated via protein–protein interactions. Specifically, HK, Bcl2, Bcl-xL, actin, and tubulin were found to interact with VDAC1 and alter its channel conductance (see VDAC1 Association with Proteins and Cancer).

Finally, high cholesterol, known to reduce the activity of membrane-associated proteins, was found to inhibit channel conductance and the metabolic function of VDAC (Campbell and Chan, 2008). It has also been reported that plant VDAC undergoes a reversible regulation of selectivity and voltage-dependence in the presence of sterols (Mlayeh et al., 2010).

## VDAC STRUCTURE–FUNCTION RELATIONSHIPS

Since their discovery in the mid-1970s, great efforts have been devoted to understanding structure–function relationships of VDACs using numerous techniques, including circular dichroism (CD), atomic force microscopy (AFM), electron microscopy, NMR, crystallography, and others.

### THE THREE-DIMENSIONAL STRUCTURE OF hVDAC1

VDAC1 is a polypeptide of 283 amino acids with Met1 missing in the mature protein (Thinnes et al., 1989; Blachly-Dyson et al., 1993). Various studies have led to the development of models postulating the transmembrane organization of VDAC1, comprising a single α-helix at the N-terminus and 13, 16, or 19 transbilayer β-strands that form a β-barrel (De Pinto et al., 1991; Song et al., 1998; Casadio et al., 2002; Colombini, 2004).

**FIGURE 3:**
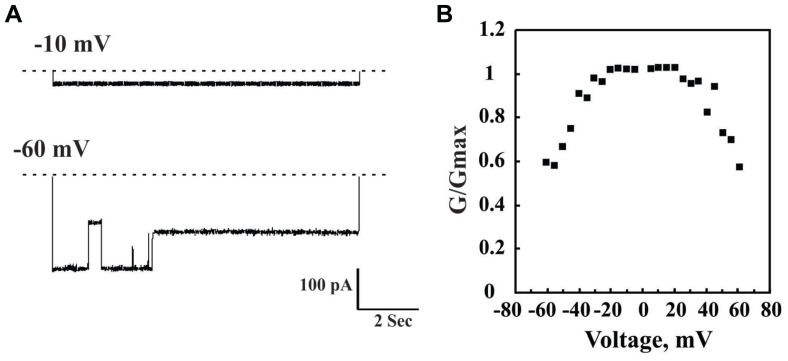
**Channel properties of bilayer-reconstituted purified VDAC1.** Bilayer-reconstituted VDAC single and multi-channel activity was assayed as described previously (Gincel et al., 2000). Purified VDAC (1–5 ng) was reconstituted into a PLB. In **(A)**, a typical activity recording of VDAC incorporated into a PLB is presented as current traces obtained in response to voltage steps from 0 mV to either -10 or -60 mV. In symmetric solution (1 M NaC), when the voltage was changed from -0 to 10 mV, the channel opens and remains stable in this conformation for up to 2 h. However, when the voltage was changed from 0 to -60 mV, the current first increased, due to a greater driving force. However, within less than 1 s, channel conductance decreased and VDAC assumed multiple conductance states. The dashed line indicates the zero current level, while the sub-states of the channel are indicated by arrowheads. In **(B)**, Multi-channel recordings of the average steady-state conductance of VDAC are presented as a function of voltage. The conductance (*G*_o_) at a given voltage was normalized to the conductance at -10 mV (*G*_max_). Each point is the average of three experiments. This voltage-dependent behavior is well known for VDAC.

Recently, the 3D structure of recombinant VDAC1 was solved using X-ray crystallography, NMR and a combination of the two. Such studies presented VDAC1 as composed of 19 β-strands arranged as a barrel, with strands β1 and β19 being in parallel conformation (**Figure 3**; Bayrhuber et al., 2008; Hiller et al., 2008; Ujwal et al., 2008). The N-terminal domain of VDAC1, consisting of 25 amino acids, was shown to be very dynamic and possesses different degrees of α-helical content in each of the three proposed structures (Bayrhuber et al., 2008; Hiller et al., 2008; Ujwal et al., 2008). All three methods employed refolded recombinant VDAC1 expressed in *E. coli* and purified from inclusion bodies. As such, it has been argued that the refolding conditions employed led to the appearance of non-native structures, as biochemical and biophysical approaches argue for the existence of additional extra-membranal VDAC regions (Colombini, 2009).

The pore diameter of the channel has been estimated to be between 3 and 3.8 nm, based on biochemical and structural methods (Colombini, 1980; Goncalves et al., 2007; Bayrhuber et al., 2008) and about 1.5 nm when the N-terminal α-helix is located inside the channel, according to the recent 3D studies (Bayrhuber et al., 2008). Finally, there is evidence that cholesterol is bound to mammalian mitochondrial VDAC and sterols seem to be important for the folding of VDAC (De Pinto et al., 1989; Hiller et al., 2008).

### THE N-TERMINAL REGION OF VDAC1: LOCATION, TRANSLOCATION, AND CHANNEL GATING

The location of the N-terminal region of VDAC1 with respect to the pore, its role in voltage-gating and its interaction with associated proteins have been the focus of intensive study, as described below.

#### Location and translocation of the N-terminal region

The VDAC N-terminal region was proposed to lie on the membrane surface (Reymann et al., 1995), to be exposed to the cytoplasm (De Pinto et al., 2003) or to cross the membrane (Colombini, 2004). The 3D structures of recombinant VDAC1, however, revealed that the N-terminal domain is located inside the channel pore, causing a partial obstruction of the wide pore, possibly providing structural reinforcement to the channel walls (Bayrhuber et al., 2008; Hiller et al., 2008; Ujwal et al., 2008; Schneider et al., 2010; **Figure 3**). Deletion of the N-terminal helical domain did not affect the correct mitochondrial targeting of the protein (De Pinto et al., 2007).

Various studies suggesting that the N-terminal region of VDAC1 constitutes a mobile component of the protein (see VDAC1 N-terminal Domain Function in Voltage-gating and Cell Death). In 2D crystals of *Neurospora crassa* VDAC1, oblique arrays of the N-terminal domain appear to extend laterally from the barrel, into the aqueous phase (Guo et al., 1995). The N-terminus of the protein was shown to be accessible to anti-VDAC1 antibodies raised against this part of the protein (Guo et al., 1995; Shoshan-Barmatz et al., 2004; Abu-Hamad et al., 2006). The same domain is exposed to kinases, as threonine-13 undergoes phosphorylation (Distler et al., 2007). Additionally, the N-terminal domain of VDAC1 interacts with cytosolic proteins and acts as a recruiting site for HK1, Bcl2, and Bcl-xL and thus is a key structural feature mediating VDAC interaction with anti-apoptotic proteins to enable their function (Shi et al., 2003b; Abu-Hamad et al., 2009; Arzoine et al., 2009; Arbel and Shoshan-Barmatz, 2010), suggesting that this VDAC region is exposed outside the pore. Finally, a recent *in silico* study supports the role of the N-terminal in controlling shape and permeability of the channel. In the absence of N-terminal, increased overall β-barrel motion, resulting in elliptic, semi-collapsed barrel shapes, is seen (Zachariae et al., 2012).

Taken together, these findings suggest that the N-terminal region of VDAC1 is loosely attached to the barrel wall and can undergo translocation to become exposed at the membrane surface. This movement allows VDAC1 to interact with the anti-apoptotic proteins, HK, Bcl-xL, and Bcl2, and may participate in VDAC1 oligomerization. The signal and nature of conformational changes inducing VDAC1 N-terminal region translocation, both under physiological and apoptotic conditions, await further study.

#### VDAC1 glycine-rich sequence

It was reported that in VDAC1, a glycine-rich sequence (GXXXG), highly conserved in mammals, connects the N-terminal domain to the β-barrel, thus providing the flexibility needed for N-terminal translocation in and out of the pore (Geula et al., 2012a). When this flexible region is mutated by replacing glycines with prolines, the N-terminal region favors a location out of the pore, thereby affecting channel gating. Moreover, in such mutants, the ability to form VDAC1 dimers was highly increased. The GXXXG motif has been linked with dimerization in proteins such as glycophorin A (Gerber and Shai, 2001), human carbonic anhydrase (Whittington et al., 2001), yeast ATP synthase (Saddar and Stuart, 2005), carnitine palmitoyltransferase (Jenei et al., 2009, and others). In VDAC1, this motif is not required for VDAC1 dimerization but may be involved in interaction with VDAC1-associated proteins (Geula et al., 2012a). Interestingly, such a motif in Bcl-xL, interacting with VDAC1 (Arbel et al., 2012), has been recently suggested to mediate VDAC interaction with other proteins (Ospina et al., 2011).

#### VDAC1 N-terminal domain function in voltage-gating and cell death

It was reported that during voltage-gating or interaction with molecules, such as cytochrome *c* (Cyto *c*), some motion of VDAC occurred (Stanley et al., 1995). N-terminal-truncated mV DAC1 was shown to exhibit high conductance at all tested voltages and was proposed to be a main effector in those apoptotic events dependent on VDAC1 (De Pinto et al., 2008; Abu-Hamad et al., 2009). Using recombinant mutated VDAC1 with the N-terminal cross-linked to channel wall, it was shown that voltage-gating was modified but not completely lost (Teijido et al., 2012). On the other hand, mobility of the N-terminal region and the contribution of this domain to channel gating and interaction with anti-apoptotic proteins were recently demonstrated using site-directed mutagenesis and cysteine substitution, together with a thiol-specific cross-linker (Geula et al., 2012a). Swapping the N-terminal domain of VDAC1 and with that of VDAC3 restores full activity of the channel (Reina et al., 2010).

Different models for the voltage-dependent gating of VDAC1 via the N-terminal region have been proposed: (i) Blockage of the pore by movement of the N-terminal domain within the lumen from the barrel wall toward the center of the channel (Hiller et al., 2008; Ujwal et al., 2008); (ii) transition of the N-terminal region from an α-helical structure that aligns with the barrel wall to a less-structured, unfolded helix element that interacts with the opposing barrel wall (Bayrhuber et al., 2008; Summers and Court, 2010), and (iii) movement of the N-terminal region into and out of the channel lumen (De Pinto et al., 2008; Abu-Hamad et al., 2009; Geula et al., 2012a; Teijido et al., 2012). Calculations using both Poisson–Boltzmann and Poisson–Nernst–Planck electrostatic equations agree with the N-terminal region being involved in gating but not via lateral or horizontal movement of the helix (Choudhary et al., 2010). Thus, the accumulated results and the derived models point to the N-terminal domain as being involved in the channel gating, although the precise mechanism has yet to be described.

#### VDAC1 C-terminal domain

In contrast to the extensive studies directed at defining N-terminal domain structure–function, very little research effort has addressed the VDAC1 C-terminal domain. Recently, a frame-shift resulting in appearance of an early stop codon in the VDAC1 gene, leading to the absence of approximately 60% of the C-terminal portion of VDAC1, was noted in gastric and colonic cancers with microsatellite instability (Yoo et al., 2011). Patients with this somatic mutation are heterozygous (Yoo et al., 2011).

A link between the VDAC1 C-terminal region and cancer also comes from studies connecting hypoxia and C-terminal cleavage (Brahimi-Horn et al., 2012). Hypoxic conditions were found to trigger cleavage of the VDAC1 C-terminal to yield a 26-kDa protein (Brahimi-Horn et al., 2012). This C-terminal-cleaved VDAC1 was regulated by HIF-1α and was correlated with hypoxic cell survival and chemotherapy resistance (Brahimi-Horn et al., 2012). Based on the incidence of δC-VDAC1 in lung cancer (about 50% on average and higher in late stage tumors), this form of the protein was proposed to serve as a biomarker to stratify tumor progression in lung cancer patients (Brahimi-Horn et al., 2012). Finally, it was reported that an unidentified mitochondrial calpain cleaves the apoptotic induction factor (AIF) and VDAC1 in a Ca^2+^-dependent manner, and that such cleavage triggers tVDAC–Bax pores that are associated with the release of tAIF (Ozaki et al., 2009).

### VDAC1 OLIGOMERIZATION AND FUNCTION

Purified and membrane-embedded mammalian VDAC1 were shown to assemble into dimers, trimers, tetramers, and higher oligomers, as revealed by chemical cross-linking and fluorescence resonance energy transfer (FRET) analysis (Zalk et al., 2005). A low-resolution (15 Å) surface structure of VDAC1, obtained by metal shadowing and cryo-electron microscopy of human VDAC1 crystals grown in the presence of phospholipids, showed a dimeric organization of VDAC1 (Dolder et al., 1999). High-resolution AFM of purified native OMM from potato tubers (Hoogenboom et al., 2007) or yeast (Goncalves et al., 2007) showed the distribution of VDAC in an equilibrium ranging from single membrane-embedded pores to hexamers and higher-order oligomers (Hoogenboom et al., 2007), including arrays of up to 20 molecules (Goncalves et al., 2007). In addition, the use of symmetry operators on the NMR-based structure of recombinant hVDAC1 implied that it forms a dimer of monomers arranged in parallel (Bayrhuber et al., 2008), while analysis of the crystal packing of mVDAC1 revealed strong anti-parallel dimers that further assemble into hexamers (Ujwal et al., 2009).

Interestingly, VDAC1 oligomerization is highly increased upon apoptosis induction (Keinan et al., 2010). Structural and computational-based approaches, in combination with site-directed mutagenesis, cysteine replacement, and chemical cross-linking, identified the contact sites between VDAC1 molecules in dimers and higher oligomers (Geula et al., 2012b). Two forms of dimeric VDAC1, one with a contact site involving β-strands 1, 2, and 19 and the second involving β-strands 16 and 17, were identified. Moreover, the results suggest that VDAC1 exists as a dimer that undergoes conformational changes upon apoptosis induction to assemble into higher oligomeric states with contact sites also involving β-strand 8 (Geula et al., 2012b).

The function of VDAC1 oligomers is not known. It was proposed that an organization of VDAC1 beyond the monomeric or dimeric forms may contribute to stabilizing the protein (Ujwal et al., 2009). On the other hand, it was proposed that the oligomeric assembly of VDAC1 offers a platform for other proteins to oligomerize, such as HK (Zalk et al., 2005). HK-I assumes a tetrameric structure that is greatly enhanced when the enzyme is bound to mitochondria (Xie and Wilson, 1990) or when it interacts with the mitochondria to inhibit permeability transition pore (PTP) opening (Azoulay-Zohar et al., 2004). Creatine kinase, when bound to VDAC1 at the inter-membrane space, forms high order oligomers (Brdiczka et al., 1994; Stachowiak et al., 1998), interacting with VDAC1 exclusively in the octameric state, with the dimeric state only showing weak affinity for VDAC1 (Schlattner et al., 2001).

Recently, the function of VDAC1 oligomerization in apoptosis, namely mediating the pathway for the release of Cyto *c*, was proposed (Zalk et al., 2005; Shoshan-Barmatz et al., 2006, 2008b; Abu-Hamad et al., 2009; Keinan et al., 2010). VDAC1 oligomerization was strongly correlate with apoptosis induction (Shoshan-Barmatz et al., 2008b, 2009; Abu-Hamad et al., 2009; Keinan et al., 2010), while apoptosis induction by various inducers was accompanied by an up to 20-fold increase in VDAC1 oligomerization, indicating a shift in VDAC1 organization toward the oligomeric form under such conditions (Shoshan-Barmatz et al., 2008b; Keinan et al., 2010; see VDAC1 Oligomerization and Release of Cytochrome *c*).

## VDAC1 AND MITOCHONDRIA-MEDIATED APOPTOSIS

In apoptosis, a multi-step process that can be initiated by a variety of stimuli, a cascade of cysteine proteases, caspases, are activated, subsequently leading to organized cell demise. Defects in the regulation of apoptosis are often associated with various diseases, with the ability of cells to evade apoptosis being a hallmark of cancer (Hanahan and Weinberg, 2000). Alterations of apoptosis are involved in tumorigenesis, as well as in cellular responses to anti-tumor treatments (Hickman, 2002; Johnstone et al., 2002).

### THE EXTRINSIC AND INTRINSIC APOPTOTIC PATHWAYS

Apoptosis can be activated via the extrinsic or intrinsic pathways. In the extrinsic pathway, apoptosis is induced by extrinsic apoptotic signals initiated by ligand engagement of cell surface receptors, such as Fas and TNF receptors. The intrinsic pathway is initiated in mitochondria in response to different stimuli (Green and Evan, 2002; Johnstone et al., 2002; Chowdhury et al., 2006). Receptor binding by an extrinsic signal typically leads to the recruitment of adapter proteins that promote caspase oligomerization and auto-processing. The extrinsic apoptotic pathway can induce activation of the intrinsic pathway via caspase 8-dependent cleavage of Bid and translocation of the truncated form (tBid) to the mitochondria (Korsmeyer et al., 2000; Yin, 2000). Thus, apoptotic signals initiated by death receptors can be linked to mitochondria-mediated apoptosis.

The intrinsic apoptotic pathway involves mitochondria. Mitochondria contain an arsenal of apoptogenic factors, normally residing in the inter-membranal space (IMS), such as Cyto *c*, AIF, Smac/DIABLO, and endonuclease G. During transduction of an apoptotic signal into the cell, an alteration in mitochondrial permeability occurs, causing the release of these apoptogenic factors from the IMS (Green and Evan, 2002; Chowdhury et al., 2006; Shoshan-Barmatz et al., 2010). These proteins participate in complex processes resulting in the activation of proteases and nucleases, leading to protein and DNA degradation, and ultimately, cell death. Most notable among the released protein is Cyto *c* that initiates apoptosis by binding to a central apoptotic regulator, Apaf-1, promoting oligomerization of Apaf-1 and activation of caspase 9, which subsequently activates effector caspases, such as caspases 3, 6, and 7, encouraging execution of cell death. How Cyto *c* and other apoptogenic factors are released from mitochondria is not clear, and will be discussed below.

Mitochondria-mediated apoptosis can be induced in response to different stimuli (Costantini et al., 2000), including high levels of cytoplasmic Ca^2+^, reactive oxygen species (ROS), activation of pro-apoptotic Bcl-2 family proteins (Le Bras et al., 2005; Keeble and Gilmore, 2007; Kroemer et al., 2007) or UV damage (Denning et al., 2002). Chemotherapeutic agents act via different mechanisms to induce mitochondria-dependent apoptosis (Costantini et al., 2000). These include betulinic acid (Fulda et al., 1998), PK11195 (Hirsch et al., 1998), diamide (Zamzami et al., 1998), lonidamine (LND; Ravagnan et al., 1999), arsenite (Larochette et al., 1999), 6[3-adamantyl-4-hydroxyphenyl]-2-naphthalene carboxylic acid (CD437; Marchetti et al., 1999), 2-chloro-2′-deoxyadenosine, 2-chloro-2′-ara-fluorodeoxy-adenosine (Genini et al., 2000), MT-21 (Watabe et al., 2000), verteporfin (Belzacq et al., 2001), resveratrol (Tinhofer et al., 2001), and paclitaxel (Andre et al., 2000).

As mitochondria represent an appropriate target for therapeutic agents designed to modulate apoptosis, continued research into the mechanisms of mitochondria-mediated apoptotic cell death, coupled with further characterization of the released molecules, will offer new and promising targets for chemotherapeutic intervention in a host of pathologies.

### VDAC1 AND APOPTOSIS

The involvement of VDAC1 in mitochondria-mediated apoptosis has been proposed based on several lines of experimental evidence: (a) Anti-VDAC1 antibodies specifically and effectively prevent As_2_O_3_-induced Cyto *c* release from isolated mitochondria (Zheng et al., 2004) and when microinjected into cells, prevented Bax-induced Cyto *c* release and subsequent apoptosis, as well as etoposide-, paclitaxel-, and staurosporine (STS)-induced apoptosis (Shimizu et al., 2001). Anti-VDAC1 antibodies also inhibited the interaction of Bax with VDAC and the triggering of cell death (Madesh and Hajnoczky, 2001; Shimizu et al., 2001; Zheng et al., 2004). Microinjection of anti-VDAC antibodies into primary rat hepatocytes effectively prevents apoptosis and Bax–VDAC interactions, as induced by ethanol (Adachi et al., 2004); (b) VDAC1 is the proposed target of pro- and anti-apoptotic members of the Bcl2 family and of HK-I and HK-II (Shimizu et al., 2001; Shi et al., 2003b; Azoulay-Zohar et al., 2004; Zaid et al., 2005; Arbel and Shoshan-Barmatz, 2010; see VDAC1 Transport of Ca^2+^ and Function in ER-mitochondria Cross-talk); (c) inhibition of Cyto *c* release and cell death mediated by HK occurred in cells expressing native but not mutated VDAC1 (Zaid et al., 2005; Abu-Hamad et al., 2008; Arzoine et al., 2009); (d) RuR, interacting with Ca^2+^-binding proteins, interacts with native but not mutated VDAC1 to prevent Cyto *c* release and cell death (Zaid et al., 2005; Israelson et al., 2008); (e) siRNA-mediated down-expression of VDAC1 prevented cell death and activation of Bax induced by cisplatin and strongly reduced cisplatin-induced release of Cyto *c* and AIF, as well as the maturation of caspases-3 (Tajeddine et al., 2008). Similarly, reducing VDAC1 expression by siRNA attenuated endostatin-induced apoptosis (Yuan et al., 2008); (f) over-expression of human, murine, yeast, and rice VDAC induce apoptotic cell death (Godbole et al., 2003; Zaid et al., 2005; Abu-Hamad et al., 2008); (g) knockdown of VDAC1 in non-small cell lung cancer (NSCLC) cells inhibited TRAIL-induced activation of caspase-8 and subsequent apoptosis (Chacko et al., 2010); and (h) release of Cyto *c* was obtained using purified VDAC1 reconstituted into liposomes in which Cyto *c* had been encapsulated (Madesh and Hajnoczky, 2001; Zalk et al., 2005). Finally, (i) growth factor removal from the medium resulted in reduced nucleotide exchange between mitochondria and cytosol, leading to Cyto *c* release and apoptosis (Vander Heiden et al., 2000).

In general, there are two views as to the relationship between the conducting state of the VDAC1 channel and cell death. While one view suggests that closure, rather than opening of VDAC leads to OMM permeabilization and apoptosis (Vander Heiden et al., 2000), the second model suggest a distinct VDAC1-based conducting pathways, namely a pore formed in the center of a VDAC1 oligomer (Shimizu et al., 1999; Zalk et al., 2005; Shoshan-Barmatz et al., 2006; see VDAC1 Oligomerization and Function).

### PROPOSED MECHANISMS OF CYTOCHROME *c* RELEASE FROM THE MITOCHONDRIA

**FIGURE 4:**
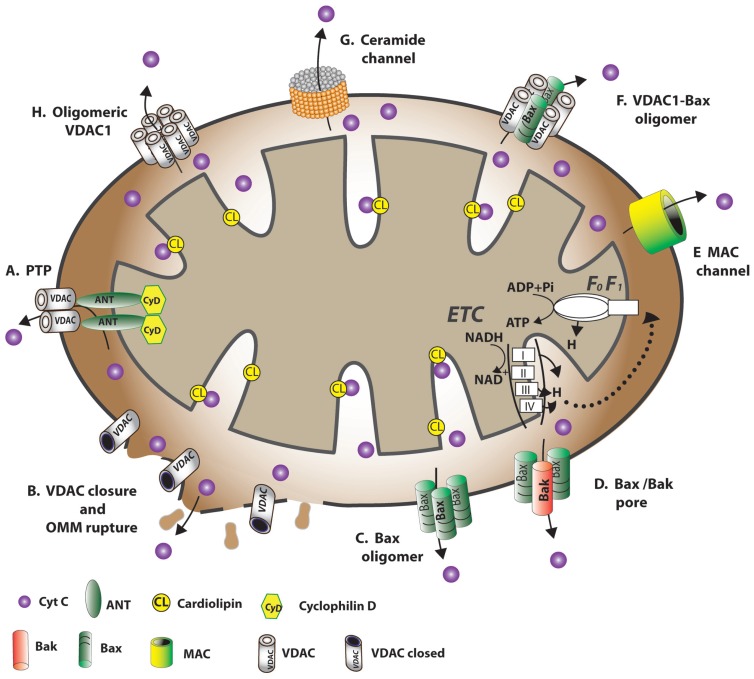
**Schematic representation of proposed models for the release of apoptogenic proteins from the mitochondrial inter-membrane space mediating the mitochondrial death decision.** Different models explaining how OMM permeability changes during apoptosis induction, allowing the release of apoptogenic factors, such as Cyto *c*. **(A)** A permeability transition pore (PTP) provides the apoptogenic proteins release pathway. It is proposed that a large conductance pore-forming complex, the PTP, composed of VDAC at the OMM, ANT at the IMM and CypD in the matrix, allows apoptogenic protein release. **(B)** VDAC closure and OMM rupture serves as the cytochrome c release pathway. Prolonged VDAC closure leads to mitochondrial matrix swelling, OMM rupture, and hence, the appearance of a non-specific release pathway for apoptogenic proteins. **(C)** Bax activation followed by its oligomerization resulting in OMM permeabilization. Upon apoptosis induction, Bax became associated with mitochondria as a large oligomer/complex, forming a Cyto *c*-conducting channel in the OMM. **(D)** A pore formed by oligomerized forms of Bax and Bak after their activation by tBID. BH3-only proteins (e.g., Bid) induce oligomerization of Bax/Bak on the OMM, resulting in Bax activation and OMM permeabilization. **(E)** MAC as the release pathway. The mitochondrial apoptosis-induced channel, MAC, is a high-conductance channel that forms during early apoptosis and is a putative cytochrome *c* release channel. MAC formation occurs without loss of outer membrane integrity and depolarization. Members of the Bcl-2 family of proteins regulate apoptosis by controlling the formation of MAC. **(F)** A Bax- and VDAC-based hetero-oligomer mediates cytochrome c release. The interaction of pro-apoptotic proteins (Bax/Bak) with VDAC forms a cytochrome *c* release pathway. **(G)** A lipid channel formed by the lipid, ceramide. Ceramides were shown to induce apoptosis via direct action on mitochondria. A self-assembled ceramide channel is proposed to act as the apoptotic protein release pathway. **(H)** Oligomeric VDAC1 as a channel for the release of apoptotic proteins. A protein-conducting channel is formed within a VDAC1 homo-oligomer. VDAC1 oligomerization thus functions in mitochondria-mediated apoptosis (see VDAC1 Oligomerization and Release of Cytochrome *c*). The dynamic equilibrium between VDAC monomeric and oligomeric states can be regulated by various factors, such as Ca^2+^, oxidative stress and cytochrome *c*.

Several proposals regarding the mechanism of Cyto *c* crossing the OMM have been proposed (**Figure 4**). However, none of the current models of mitochondrial membrane permeabilization can account for all of the experimental data.

In healthy cells, Cyto *c* is located in the mitochondrial IMS, where it serves as an electron shuttle between complexes III and IV of the respiratory chain, with most Cyto *c* being bound to cardiolipin (CL). Signals inducing mitochondria-mediated apoptosis result in the release of IMS proteins, including Cyto *c* (see The Extrinsic and Intrinsic Apoptotic Pathways). The mechanisms by which Cyto *c* and other pro-apoptotic effector molecules are released have challenged many researchers and several competing models have been proposed to explain the workings of this event (**Figure 4**; Shoshan-Barmatz et al., 2008a). While some models suggest that the proteins crossing the OMM exclusively involve an increase in OMM permeability due to the formation of a channel large enough to allow for the release of proteins, such as Cyto *c*, others consider efflux of the proteins to be due to disruption of OMM integrity. The following briefly describes the various proposed models:

#### The permeability transition pore and the release of pro-apoptotic proteins

One model (Model A, **Figure 4**) for mitochondrial membrane permeability (MMP) suggests the formation of a PTP, a large high-conductance multi-protein complex comprising several components and spanning both mitochondrial membranes (Bernardi, 1999; Lemasters et al., 1999; Halestrap and Green, 2000; Shoshan-Barmatz and Gincel, 2003; Tsujimoto and Shimizu, 2007). PTP opening is followed by Ca^2+^ accumulation in the matrix, leading to a sudden increase in permeability to solutes (up to 1,500 Da; Bernardi, 1999; Lemasters et al., 1999; Halestrap and Green, 2000; Shoshan-Barmatz and Gincel, 2003; Tsujimoto and Shimizu, 2007). This Ca^2+^-dependent increase in MMP leads to loss of membrane potential, mitochondrial swelling, and rupture of the OMM. According to this model, other factors, such as changes in the energetic balance of the mitochondria, anoxia, and ROS can also induce MMP due to PTP opening (reviewed in Tsujimoto and Shimizu, 2007).

The proposed PTP complex components include VDAC1 at the OMM, ANT in the inner mitochondrial membrane (IMM), and cyclophilin D (CypD) in the matrix (Bernardi, 1999; Green and Evan, 2002; Shoshan-Barmatz and Gincel, 2003; Tsujimoto and Shimizu, 2007). Recent studies on the PTP, however, have raised doubts about the proposed members of the complex and the importance of PTP in triggering apoptosis (Kroemer, 1997; Kokoszka et al., 2004; Belizario et al., 2007). Mitochondria isolated from animal models by knockout of genes encoding one or more ANT or VDAC isoforms exhibit Ca^2+^- and oxidative stress-induced PTP opening, suggesting that the proposed components of the standard model of the PTP complex needs to be reconsidered and further characterized (Kokoszka et al., 2004; Krauskopf et al., 2006; Baines et al., 2007; Berridge et al., 2009). On the other hand, CypD was found to be essential for MMP mediated by Ca^2+^ overload and that the CypD-dependent MMP regulates some forms of necrotic cell death but not apoptotic death (Belizario et al., 2007).

Although the mechanism(s) responsible for PTP opening and its physiological function have not yet been resolved, a variety of agents were found to promote or inhibit PTP opening, including Ca^2+^, inorganic phosphate, various oxidizing agents, glutamate, nucleotides, CypD ligands, gelsolin, HK, and proteins of the Bcl-2 family. Some of these compounds have also been shown to interact with VDAC1 directly and modify its channel activity (Shimizu et al., 2000c; Sugiyama et al., 2002; Tsujimoto and Shimizu, 2002; Shoshan-Barmatz and Gincel, 2003; Gincel and Shoshan-Barmatz, 2004; Shoshan-Barmatz et al., 2006). Similarly, the ANT ligands, atractyloside and bongkrekic acid, modulate PTP opening (Haworth and Hunter, 2000). The function of PTP in apoptosis and necrosis (Rasola and Bernardi, 2011) and as a potential therapeutic target for cancer, ischemia-reperfusion injury, and neurodegeneration were proposed (Peixoto et al., 2012).

#### Osmotic matrix swelling and OMM rupture leading to non-specific release of inter-membrane proteins into the cytosol

This model proposes that a sudden increase in IMM permeability to solutes of low molecular weight leads to mitochondria swelling and rupture of the OMM, allowing the efflux of IMS proteins, such as Cyto *c*, to the cytosol (Feldmann et al., 2000). It has been suggested that matrix swelling and OMM rupture result from a defect in mitochondrial ATP/ADP exchange due to VDAC1 closure, as a result, for example, of removal of a normal growth factor or exposure to G3139 (Model B, **Figure 4**; Vander Heiden et al., 2000, 2001; Lemasters and Holmuhamedov, 2006). Several studies, however, demonstrated that Cyto *c* release preceded membrane potential (Δψ_m_) loss in cerebellar granule neurons undergoing apoptotic death and was not accompanied by mitochondrial swelling or OMM rupture (Al-Abdulla et al., 1998; Desagher et al., 1999; Wigdal et al., 2002).

#### Bax oligomers constitute the OMM cytochrome *c*-conducting channel

A third proposed mechanism for Cyto *c* release suggests that oligomeric Bax forms the Cyto *c*-conducting channel in the OMM (Model C, **Figure 4**; Antonsson et al., 2000, 2001; Eskes et al., 2000; Zamzami et al., 2000; Kuwana et al., 2002; Reed, 2006). Upon apoptosis induction with STS or UV irradiation, Bax became associated with mitochondria as a large oligomer/complex of 96–260 kDa. While tBid enhances oligomerization of Bax (Lovell et al., 2008), Bcl-2 prevented Bax oligomerization and insertion into the mitochondrial membrane. Several studies, however, showed that apoptosis can be induced in the absence of Bax (Lindenboim et al., 2005; Mizuta et al., 2007; Wan et al., 2008), suggesting that the protein is not obligatory for apoptosis induction and that other mechanisms exist.

#### Bax and bak oligomers form pores for pro-apoptotic factor efflux during apoptosis

The appearance of a channel formed by Bax and Bak hetero-oligomers upon apoptotic insult enabling efflux of pro-apoptotic effectors has been suggested (Model D, **Figure 4**; Gross et al., 1998; Desagher et al., 1999; Wei et al., 2000, 2001; Antignani and Youle, 2006). tBid was proposed to activate the generation of Bax and Bak complexes up to 500 kDa (Sundararajan et al., 2001). At the same time, anti-apoptotic proteins, such as Bcl-2 and Bcl-xL, were shown to protect cells from apoptosis via a blockage of the Bax–Bak interaction, subsequently preventing Cyto *c* release (Mikhailov et al., 2003; Dlugosz et al., 2006).

#### Mitochondrial apoptosis-induced channel as a pathway for cytochrome *c* release

Mitochondrial apoptosis-induced channel (MAC), a supra-molecular high-conductance channel in the OMM, is thought to assemble during early apoptosis and serve as the Cyto *c* release channel that is regulated by Bcl-2 family members (Guo et al., 2004; Martinez-Caballero et al., 2004, 2005; Dejean et al., 2005, 2006a,b; Model E, **Figure 4**). The complete molecular identity of MAC is unknown. Recently, it was proposed that Bax is an essential constituent of MAC in some systems, as the electrophysiological characteristics of MAC are very similar to those of Bax channels, while depletion of Bax significantly diminishes MAC activity (Martinez-Caballero et al., 2009).

#### Hetero-oligomers composed of VDAC1 and bax form the apoptotic protein release channel

The formation of hetero-oligomers composed of VDAC1 and Bax were also proposed as a mechanism for Cyto *c* efflux (Model F, **Figure 4**; Shimizu et al., 1999; Shimizu and Tsujimoto, 2000; Banerjee and Ghosh, 2004). It was found that recombinant Bax induced permeability in liposomes containing VDAC, implying that VDAC can induce membrane permeability in the presence of Bax (Shimizu and Tsujimoto, 2000). In addition, intracellular microinjection of anti-VDAC antibodies prevented Bax-induced Cyto *c* release (Shimizu et al., 2001). Electrophysiological studies of Bax and VDAC in PLB revealed that when combined, single-channel conductance rises by factors of 4 and 10 over values attained with VDAC and Bax channels alone, respectively (Banerjee and Ghosh, 2004). Moreover, HK-I and HK-II compete with Bax for interaction with VDAC1 (Pastorino et al., 2002). It was also demonstrated that siRNA-mediated down-expression of VDAC1 strongly suppressed cisplatin-induced activation of Bax (Tajeddine et al., 2008). Finally, another version of this model suggests that oligomeric VDAC is the prime Cyto *c* release channel and that its pore is regulated by Bax (Debatin et al., 2002).

#### Ceramides and the release of cytochrome *c*

A self-assembled ceramide-based lipid channel that forms in the OMM was suggested as being the Cyto *c* release pathway (Model G, **Figure 4**; Siskind et al., 2006; Stiban et al., 2008). Ceramides were postulated to form a pore in the OMM with a diameter large enough to accommodate Cyto *c* (Siskind and Colombini, 2000; Siskind et al., 2006; Stiban et al., 2008). An alternative mechanism proposes that ceramides promote the dissociation of Cyto *c* by altering IMM lipid microdomains (Yuan et al., 2003). Another proposal suggest that ceramides and cholesterol both affect membrane microenvironments so as to favor Bax activation (Birbes et al., 2005; Oh et al., 2006), with translocation to mitochondria fostering propagation of the apoptotic cascade (Martinez-Abundis et al., 2009).

#### VDAC1 oligomerization and release of cytochrome *c*

In a model describing Cyto *c* release developed in our group, it is proposed that mitochondrial pore formation during apoptosis involves the assembly of homo-oligomers of VDAC1 (Model H, **Figure 4**; Zalk et al., 2005; Shoshan-Barmatz et al., 2006, 2008a; Abu-Hamad et al., 2009). This model is based on a VDAC1 diameter pore (2.5–3.0 nm; Bayrhuber et al., 2008) being large enough for the movement of nucleotides and small molecules but too small to allow passage of a folded protein, like Cyto *c*. Thus, a model in which Cyto *c* release takes place through the formation of large protein-conducting channel within a VDAC1 homo-oligomer or in a hetero-oligomer containing VDAC1 and pro-apoptotic proteins formed by oligomerization of VDAC1 is offered (Zalk et al., 2005; Shoshan-Barmatz et al., 2006, 2008a; Abu-Hamad et al., 2009). Substantial evidence for the formation of higher ordered VDAC1-containing complexes exists (see VDAC1 Oligomerization and Function).

**FIGURE 5:**
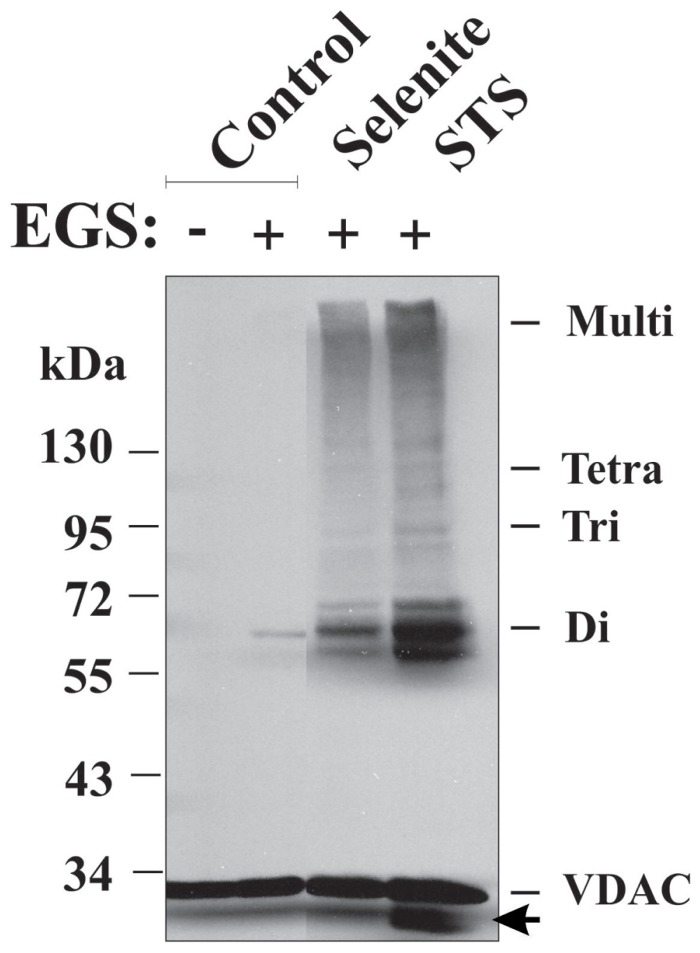
**Apoptosis induction and VDAC1 oligomerization.** VDAC1 oligomerization as induced by apoptosis inducers and revealed by EGS-based cross-linking. HeLa cells were incubated for 16 h with STS (0.2 μM) or selenite (8 μM), as described previously (Keinan et al., 2010), washed with PBS and incubated with EGS (300 μM) at 30^°^C for 10 min, followed by SDS-PAGE (10% acrylamide) and Western blotting using anti-VDAC1 antibodies. VDAC1 monomers, dimers, trimers, tetramers, and multimers are indicated. The arrow indicates an anti-VDAC1 antibody-labeled protein band migrating below the position of monomeric VDAC1. The positions of molecular weight protein standards are also provided.

Recently, it has been demonstrated that VDAC1 undergoes oligomerization in response to apoptotic stimuli, with VDAC1 oligomerization being enhanced up to 20-fold, as revealed by chemical cross-linking, or directly monitored in living cells using bioluminescence resonance energy transfer (BRET; Shoshan-Barmatz et al., 2008a; Keinan et al., 2010). Enhancement of VDAC oligomerization was obtained regardless of the cell type or apoptosis inducer used, including STS, curcumin, As_2_O_3_, etoposide, cisplatin, selenite, TNF-α, H_2_O_2_, or UV, all affecting mitochondria yet acting through different mechanisms (Keinan et al., 2010; **Figure 5**). Conversely, the apoptosis inhibitor, DIDS, prevented STS-induced VDAC1 oligomerization and apoptosis (Shoshan-Barmatz et al., 2008a; Keinan et al., 2010). Moreover, VDAC1 over-expression resulted in VDAC1 oligomerization and apoptosis in the absence of any apoptosis stimuli (Shoshan-Barmatz et al., 2008a). Furthermore, it was demonstrated that in cells expressing a VDAC1 dimeric fusion protein comprising wild type and the RuR-insensitive E72Q-mutated VDAC1 showed no protection against STS-induced apoptosis. This dominant-negative VDAC1 mutant reveals oligomeric VDAC1 to be the active unit in mitochondria-mediated apoptosis (Mader et al., 2010).

The molecular mechanisms triggering VDAC1 oligomerization upon apoptosis induction remain unknown. Post-translational modification of VDAC1, such as phosphorylation (Kerner et al., 2012) or oxidation, may modulate its oligomeric state. The involvement of lipids has been suggested recently (Betaneli et al., 2012). The anionic lipid, phosphatidylglycerol (PG), was found to significantly enhance VDAC1 oligomerization in the membrane, whereas CL disrupts VDAC1 supra-molecular assemblies. Interestingly, during apoptosis, the level of PG in mitochondria increases, whereas that of CL decreases (Betaneli et al., 2012). Another proposed mechanism involves Cyto *c* detachment from its binding site, namely CL at the IMM. Apoptotic signals leading to the oxidation of CL and dissociation of Cyto *c* from the lipid (Ott et al., 2002), together with our finding that VDAC1 oligomerization is highly encouraged by Cyto *c* (Zalk et al., 2005), support a scenario whereby unbound Cyto *c* promotes VDAC oligomerization, Cyto *c* release and subsequent apoptosis.

Clearly, a more complete understanding of the mechanisms underlying VDAC1 oligomer assembly and its role in Cyto *c *release will require additional study. Indeed, the multiple pathways and mechanisms of Cyto *c* release presented here may co-exist within a single model of cell death, depending on the cell type and the nature of the stimulus (Gogvadze et al., 2006; Galluzzi and Kroemer, 2007).

## CANCER METABOLISM, HEXOKINASE, AND VDAC

In recent years, a substantial body of evidence has accumulated indicating a correlation between alterations in cell metabolism and cancer formation. Cancer cells undergo significant metabolic adaptation to fuel cell growth and division (Gatenby and Gillies, 2004; Hanahan and Weinberg, 2011; Koppenol et al., 2011). Malignant cancer cells typically display high rates of glycolysis even when fully oxygenated and are subject to suppressed mitochondrial respiration, despite the fact that glycolysis is a less energy-efficient pathway, a phenomenon known as the “Warburg effect” (Gatenby and Gillies, 2004; Hanahan and Weinberg, 2011; Koppenol et al., 2011). The Warburg effect likely provides the vast majority of cancerous tumors with a number of benefits in the form of precursors for the biosynthesis of nucleic acids, phospholipids, fatty acids, cholesterol, and porphyrins. A second advantage of the Warburg effect is its likely involvement in both tumor protection and invasion. Tumor cells produce lactic acid via glycolysis and transport it out of the cell, leading to increased acidity of the closed microenvironment, generating a low pH “coat.” This is proposed to protect tumors against attack by the immune system while inducing negative effects on normal surrounding cells, aiding in preparing the surrounding tissues for invasion. Additionally, the Warburg effect also assures longer tumor survival time if oxygen becomes limiting (Hanahan and Weinberg, 2011). Moreover, cancer-associated abnormalities in glucose metabolism enhance cellular resistance to apoptosis, with mitochondria playing a key role in this process (Fadeel et al., 2008; Kroemer and Pouyssegur, 2008; Mayevsky, 2009; Fulda et al., 2010; Gogvadze et al., 2010). Finally, mitochondria have been found to playa role in cellular re-programming from the catabolic to the anabolic modes (Fadeel et al., 2008; Kroemer and Pouyssegur, 2008; Mayevsky, 2009; Gogvadze et al., 2010), with such metabolic flexibility and cellular hierarchy being crucial in metastatic cancer (Berridge et al., 2010).

Such metabolic re-programming of cancer cells includes marked over-expression of mitochondrial-bound HK isoforms, considered as the rate-limiting enzyme of glycolysis and serving as the biochemical gate of this pathway (Pedersen et al., 2002; Mathupala et al., 2006; Pedersen, 2007; Shoshan-Barmatz et al., 2010). HK interacts with VDAC1 at the OMM and forms the main interface between mitochondria and the cytosol. VDAC1 contributes to cancer metabolism via transport of various metabolites, mediating ATP/ADP exchange across the OMM and is, therefore, defined as the “food channel.” HK, by association with VDAC1, gains direct access to mitochondrial ATP, reaching VDAC1 via the ANT in the IMM, allowing it to phosphorylate and “trap” any incoming glucose (Pedersen, 2008). With this direct coupling of mitochondrially generated ATP to incoming glucose via VDAC1-bound HK, mitochondria regulate glycolytic flux with that of the tricarboxylic acid (TCA) cycle and ATP synthase to balance the energy requirements of the tumor cell with biochemical requirements for metabolites (i.e., the anaplerotic and cataplerotic pathways, respectively) or metabolite precursors that are required by the tumor (Pedersen et al., 2002; Mathupala et al., 2006; Pedersen, 2007; Shoshan-Barmatz et al., 2010). Thus, both the glycolytic pathway and other seminal metabolic pathways, like the pentose phosphate shunt, are regulated via the energy-coupling resulting from the formation of the VDAC1-bound HK complex. As part of a system that impacts cell growth, the VDAC1–HK complex represents a remarkable target for cancer therapy (see The Interaction of VDAC1 with Hexokinase Regulates Cell Bioenergetics and Apoptosis).

## VDAC1 ASSOCIATION WITH PROTEINS AND CANCER

The localization of VDAC1 at the OMM provides structural and functional anchoring sites for a diverse set of cytosolic proteins that together with VDAC1 mediate metabolic and signaling cross-talk between cytosol and mitochondria (**Table 1**). VDAC1 displays binding sites for glycerol kinase (Adams et al., 1991), HK (Azoulay-Zohar et al., 2004; Pastorino et al., 2005; Abu-Hamad et al., 2008; Goldin et al., 2008; Shoshan-Barmatz et al., 2008b; Arzoine et al., 2009), creatine kinase (Schlattner et al., 2001), C-Raf kinase (Le Mellay et al., 2002), ANT (Vyssokikh and Brdiczka, 2003), the peripheral benzodiazepine receptor [also known as translocator protein (TSPO); Veenman et al., 2007], tubulin (Rostovtseva et al., 2008b), the dynein light chain, mtHSP70, the ORDIC channel, GAPDH (Shoshan-Barmatz and Israelson, 2005), actin (Xu et al., 2001), and gelsolin (Kusano et al., 2000), as well as Bcl-2 family members (Shoshan-Barmatz et al., 2006; Adams and Cory, 2007; Llambi and Green, 2011). Interaction with apoptosis-regulating proteins, such as HK and Bcl-2 family members, make VDAC1 a key protein in apoptosis regulation (Tsujimoto and Shimizu, 2002). Here, we focus on those VDAC1-interacting proteins showing modified levels of expression in cancer cells.

**Table 1:** Modulation of VDAC conductance and mitochondrial-mediated apoptosis by VDAC-associated proteins.

Protein	VDAC conductance	PTP opening	Cyto *c* release	Cell death	Interaction demonstrated by
GAPDH	ND	Activation (Berry and Boulton, 2000)	Activation (Tarze et al., 2007)	Activation (Berry and Boulton, 2000)	Affinity-chromatography (Shoshan-Barmatz and Israelson, 2005)
Bcl2	Reduction (Arbel and Shoshan-Barmatz, 2010)	ND	Inhibition (Hou et al., 2003)	Protection (Cory and Adams, 2002; Cory et al., 2003)	ND
Bax^a^	Increase (Shimizu et al., 1999,2000a; Banerjee and Ghosh, 2004)	Activation (Priault et al., 1999)	Increase (Shimizu et al., 1999,^2001^; Antonsson, 2001)	Induction (Shimizu et al., 2001; Pastorino et al., 2002)	Liposome (Shimizu et al., 2000c); immunoprecipitation (Shimizu et al., 1999)
HK-I	Reduction (Azoulay-Zohar et al., 2004; Zaid et al., 2005)	Inhibition (Azoulay-Zohar et al., 2004)	Inhibition (Azoulay-Zohar et al., 2004; Abu-Hamad et al., 2008)	Protection (Azoulay-Zohar et al., 2004; Zaid et al., 2005; Abu-Hamad et al., 2008)	SPR with VDAC1 peptides (Arzoine et al., 2009); site directed mutagenesis (Zaid et al., 2005; Abu-Hamad et al., 2008; Arzoine et al., 2009)
HK-II	ND	ND	Inhibition (Pastorino et al., 2002)	Protection (Pastorino et al., 2002)	Site directed mutagenesis (Arzoine et al., 2009)
hGelsolin^b^	Inhibition (Kusano et al., 2000)	Activation (Kusano et al., 2000)	Inhibition (Kusano et al., 2000; Qiao and McMillan, 2007)	Inhibition (Kusano et al., 2000)	Immunoprecipitation (Kusano et al., 2000)
Bcl-Xl	Reduction (Shimizu et al., 1999,2000c; Arbel et al., 2012)	ND	Inhibition (Shimizu et al., 1999)	Protection (Cory and Adams, 2002; Cory et al., 2003)	Immunoprecipitation (Shimizu et al., 1999); reconstituted membranes (Shimizu et al., 2000c); NMR (Malia and Wagner, 2007)
Bak	ND	ND	Increase (Shimizu et al., 1999)	Induction (Tajeddine et al., 2008)	Reconstituted membranes (Shimizu et al., 2000c)
ANT	ND	Activation (Vieira et al., 2000)	Increase (Brdiczka et al., 2006)	Induction (Chittenden et al., 1995)	Co-purification (Beutner et al., 1996,^1998^); co-immunoprecipitation (Capano and Crompton, 2002); purified proteins interaction (Bühler et al., 1998)
CK	ND	ND	ND	ND	SPR, co-purification (Beutner et al., 1998)
Actin	Modulates gating (Xu et al., 2001)			Induction (Tang et al., 2006)	SPR (Roman et al., 2006); reconstituted membranes (Xu et al., 2001)
Tubulin	Reduction (Rostovtseva et al., 2008b)	ND	ND	Inhibition (Romagnoli et al., 2011)	Immunoprecipitation (Carre et al., 2002)
tBid	Reduction (Rostovtseva et al., 2004)	Activation (Csordas et al., 2002)	Induction (Csordas et al., 2002; Brustovetsky et al., 2003)	Induction (Zamzami et al., 2000)	Reconstituted membranes (Rostovtseva et al., 2004)
TSPO	ND	Activation (Pastorino et al., 1994)	ND	ND	Co-purification (McEnery, 1992)
eNOS	ND	ND	ND	ND	Co-immunoprecipitation (Sun and Liao, 2002)
mtHSP70	Reduction (Schwarzer et al., 2002)	Inhibition (He and Lemasters, 2003)	ND	Inhibition	Two-hybrid system (Schwarzer et al., 2002)
Dynein light chain	Increase (Schwarzer et al., 2002)	ND	ND	ND	Two-hybrid system (Schwarzer et al., 2002)

a *Bax oligomerizes with VDAC (Adachi et al., 2004)*.

b Ca^2+^-dependent binding to VDAC.

### THE INTERACTION OF VDAC1 WITH HEXOKINASE REGULATES CELL BIOENERGETICS AND APOPTOSIS

#### HK expression and bioenergetics regulation in cancer cells

One of the signature phenotypes of highly malignant, poorly differentiated tumors is their high rate of glycolysis, leading to enhanced lactate generation (Brahimi-Horn et al., 2007). This property is frequently dependent on the marked over-expression of VDAC1-bound HK (Rose et al., 1974; Gottlob et al., 2001; Bryson et al., 2002; Pedersen et al., 2002; see Cancer Metabolism, Hexokinase and VDAC). HK thus lies at the apex of the glycolytic pathway that provides the metabolic intermediates required by the biosynthetic pathways on which a transformed cell places such heavy demand (Pedersen, 2007; Mathupala et al., 2010).

Hexokinase catalyzes the rate-limiting step in glycolysis, the ATP-dependent phosphorylation of glucose to G-6-P. The mitochondria bound-isoforms, HK-I and HK-II, were found to be over-expressed in many cancers, including colon, prostate, lymphoma, glioma, gastric adenomas, carcinomas, and breast cancers (Gottlob et al., 2001; Bryson et al., 2002; Mathupala et al., 2010). The elevated levels of mitochondria-bound HK in cancer cells is thus suggested to play a pivotal role in promoting cell growth and survival in rapidly growing, highly glycolytic tumors and in protecting against mitochondria-mediated cell death (Mathupala et al., 2006). The association/dissociation of HK with/from VDAC1 and the switching of VDAC1 between an “open” and a “closed” state regulate cross-talk between mitochondria and the cytosol (Shoshan-Barmatz et al., 2006, 2010). This control is important in maintaining the mitochondria respiration and glycolysis equilibrium at the heart of the energetic and metabolic homeostasis of the cancer cell. Cancer cell, however, possess an escape mechanism that intervenes when G-6-P accumulates and dissociates HK from VDAC (Azoulay-Zohar et al., 2004).

#### VDAC1 is the mitochondrial binding site of HK

The interaction of HK with the mitochondria, and specifically with VDAC1, has shifted our view of HK as predominantly fulfilling a metabolic role to one of regulation of apoptotic responsiveness of the cell, making the VDAC1–HK complex a target for therapeutic purposes (Pastorino et al., 2002; Azoulay-Zohar et al., 2004; Pastorino et al., 2005; Shoshan-Barmatz et al., 2006, 2008b, 2009; Abu-Hamad et al., 2008; Goldin et al., 2008; Pastorino and Hoek, 2008; Arzoine et al., 2009).

The interaction between HK-I and VDAC1 was first demonstrated in a reconstituted system where HK-I decreased the channel conductance of VDAC1, an effect that was reversed by G-6-P, shown to detach HK from isolated mitochondria (Azoulay-Zohar et al., 2004). The interaction between HK-I and VDAC1 was again demonstrated by co-immunoprecipitation (Shoshan-Barmatz et al., 2008a). The co-localization of HK-I with each of the three isoforms of VDAC1 was demonstrated using two-color stimulated emission depletion (STED) microscopy (Neumann et al., 2010). The HK-I–VDAC1 interaction can be abrogated upon mutagenesis of a single VDAC1 residue (Glu73, Glu202, or Glu65), resulting in an elimination of HK-mediated protection against apoptosis and channel closure (Zaid et al., 2005; Abu-Hamad et al., 2008). N-terminally truncated VDAC1 is incapable of binding HK (Abu-Hamad et al., 2009) or Bcl-xL (Arbel et al., 2012). Moreover, when the N-terminal region α-helix structure in VDAC1 was perturbed, the binding of HK was reduced (Geula et al., 2012a). It was proposed that the N-terminal region of HK-I is inserted into the channel pore, where it interacts with the N-terminal region of VDAC1 (Rosano, 2011). It should be noted that there is strong residue conservation between the HK-I and HK-II N-termini, and that such conservation is not shared by the other two non-bound mitochondrial isoforms, HK-III and HK-IV.

The VDAC1–HK interaction was shown to be negatively regulated by phosphorylation, when GSK3β is up-regulated (Pastorino et al., 2005). Finally, VDAC1-based peptides were found to interact with purified HK and when expressed in cells over-expressing HK, prevented the anti-apoptotic activity of HK (Arzoine et al., 2009).

#### HK-linked protection against cell death is mediated via interaction with VDAC1

*In vitro* and *in vivo* studies have shown that elevated levels of mitochondria-bound HK in cancer cells also protect against mitochondria-mediated apoptosis via direct interaction with VDAC1.

Mitochondrially associated HK has been shown to protect HeLa and HEK cells from entering apoptosis (Bryson et al., 2002). This protection was related to a blockade of the interaction of the pro-apoptotic protein, Bax, with VDAC1 (Bryson et al., 2002). Moreover, over-expression of HK-I or HK-II in tumor-derived cell lines suppressed STS-induced Cyto *c* release and apoptosis (Azoulay-Zohar et al., 2004; Zaid et al., 2005; Arzoine et al., 2009). A decrease in apoptosis and an increase in cell proliferation have also been reported to be induced by HK-II expression in the NIH-3T3 (Fanciulli et al., 1994) and rat 1a cell lines (Gottlob et al., 2001). Importantly, mutagenesis studies revealed that single mutations or N-terminal truncation in VDAC1 prevented HK-I-mediated protection against apoptosis and channel closure in a reconstituted membrane system (Zaid et al., 2005; Abu-Hamad et al., 2008, 2009; Shoshan-Barmatz et al., 2009). In addition, binding of HK-II to mitochondria inhibits Bax-induced Cyto *c* release and apoptosis (Pastorino et al., 2002). HK binding to VDAC1 is regulated by protein kinases, notably GSK-3β (Pastorino et al., 2005) and protein kinase C (PKC; Pastorino and Hoek, 2008), as well as by the cholesterol content of the OMM (Pastorino and Hoek, 2008).

#### HK interaction with VDAC1, advantages to cancer cells

Several mechanisms by which HK binding to VDAC1 protects against apoptosis and promotes cell survival can be considered. These include controlling energy and metabolic homeostasis, as well as preventing VDAC1-mediated Cyto *c* release and shielding VDAC1 from pro-apoptotic factor binding, thus offering the tumor cell protection from cell death in a synergic manner. The advantages to cancer cells of HK binding to VDAC1 include:

***Energy and metabolite production and access*.** Hexokinase bound to VDAC1 provides cells with metabolic advantages, allowing enhanced cell growth (Pedersen, 2007). Anchoring of HK to VDAC1 offers the enzyme direct access to mitochondrial sources of ATP and greater affinity for Mg^2+^-ATP (Bustamante and Pedersen, 1980; Pedersen, 2008). HK bound to the cytosolic face of VDAC1 acts as a gate, regulating the traffic of various metabolites through the VDAC1 (Azoulay-Zohar et al., 2004). In addition, VDAC1-bound HK is less sensitive to inhibition by its product, G-6-P (Azoulay-Zohar et al., 2004), thus avoiding product inhibition. The HK–VDAC1 interaction increases energy and metabolite production of the high energy-demanding cancer cells, allowing for maintenance of a high glycolytic flux rate in tumors (Azoulay-Zohar et al., 2004).

***VDAC1-bound HK acts as an anti-apoptotic protein*.** Accumulated evidence demonstrated that HK-I and HK-II also function as anti-apoptotic proteins when bound to VDAC1, while their detachment enabled activation of apoptosis (Pastorino et al., 2002, 2005; Azoulay-Zohar et al., 2004; Shoshan-Barmatz et al., 2006, 2008b, 2009; Abu-Hamad et al., 2008; Goldin et al., 2008; Pastorino and Hoek, 2008; Arzoine et al., 2009). Disruption of HK binding to VDAC1 by mutation in VDAC1 or by addition of VDAC1-based peptides decreased the survival of cancer cells (Arzoine et al., 2009).

Hexokinase interaction with VDAC1 protects against activation of apoptosis by Bax or Bak (Pastorino et al., 2002; Majewski et al., 2004b; Pastorino and Hoek, 2008). The detachment of HK-II from the mitochondria was found to markedly potentiate the onset of caspase-2-induced mitochondrial damage (Shulga et al., 2009).

***Regulation of ROS production by HK*.** Reactive oxygen species act as second messengers in cell signaling and are essential for multiple biological processes in normal cells. However, ROS can also provoke damage to multiple cellular organelles and processes (Auten and Davis, 2009). ROS production is usually increased in cancer cells due to oncogene activation (Zhang et al., 2011; see VDAC1 Function in ROS Release, ROS-mediated Apoptosis and Interaction with NO). Mitochondria-associated HK was shown to reduce mitochondrial ROS generation (da-Silva et al., 2004), with HK-I and HK-II reducing intracellular levels of ROS (Sun et al., 2008). Moreover, expression of both HK-I and HK-II was found to protect against oxidant-induced cell death (Ahmad et al., 2002; Bryson et al., 2002). Thus, detachment of HK from VDAC1 could lead to increased ROS generation and release to the cytoplasm, thereby activating cell death.

***Stabilization of both HK and VDAC1*.** VDAC1, containing an odd number of β-strands, presents conformational intrinsic instability within the first four β-strands (β1–4), relative to other VDAC1 regions (Bayrhuber et al., 2008).

*In silico* studies of the VDAC1–HK-I interaction predicates that both proteins attain a more stable state through protein–protein interaction (Rosano, 2011). It is proposed that upon binding with the N-terminal helix of HK-I, VDAC1 acquires higher stability via the formation of a network of chemical bonds both due to direct protein–protein contacts and to hydrogen bonds mediated by an ATP molecule and an Mg^2+^ ion (Rosano, 2011).

***Increased synthesis and uptake of cholesterol*.** Cancer cells have been shown to exhibit a 2- to 10-fold increase in mitochondrial cholesterol content, in comparison to liver mitochondria (Baggetto et al., 1992). It has been proposed that the increased binding of HK to the mitochondria of cancer cells may play a role in mediating increased synthesis and uptake of cholesterol into the mitochondria of cancer cells (Pastorino and Hoek, 2008).

#### Disruption of the HK–VDAC interaction as an approach to cancer therapy

**FIGURE 6:**
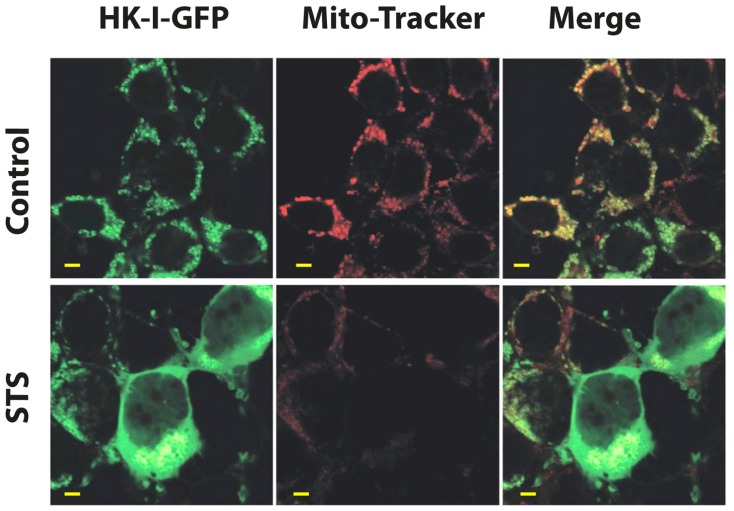
**Detachment of mitochondrial-bound HK-I-GFP induced by STS.** To demonstrate HK-I binding to mitochondria (i.e., VDAC) as well as detachment, a HK-I–GFP fusion protein was expressed in HEK–T cells. Confocal fluorescence microscopy showed that in control cells expressing HK-I–GFP, the fluorescence is punctuated, as expected for mitochondrial distribution. On the other hand, induction of apoptosis by STS detaches mitochondrial-bound HK-I–GFP. HEK–T cells were transfected to express HK-I–GFP and after 48 h were exposed to STS (0.8 μM), stained with 25 nM of the mitochondrial marker MitoTracker Red and visualized with confocal microscopy. The punctuated HK-I–GFP fluorescence, originally co-localized with mitochondria (control), as also reflected in the co-localization with MitoTracker Red (Merge panel), was converted to diffuse labeling of the cytosol after exposure to STS. Images are representative microscopic fields from one of three similar experiments (scale bar = 5 μm).

If one considers the metabolic importance of both VDAC1 and HK, the role that HK fulfills in promoting tumor cell survival and the function of the HK–VDAC1 interaction in regulating apoptosis, as well as other functions as presented above, then disruption of the HK–VDAC1 complex represents an attractive target for cancer therapy and may form the basis for novel anti-cancer drugs. Indeed, several different compounds have been employed to disrupt the HK–VDAC1 association, resulting in apoptotic cell death. These include peptides corresponding to the amino terminus of both HK-I (Gelb et al., 1992) and HK-II (Pastorino et al., 2002), clotrimazole (Penso and Beitner, 1998; Pastorino et al., 2002; Shoshan-Barmatz et al., 2008b) and a cell-permeable HK-II-based peptide (Majewski et al., 2004a). Methyl jasmonate (a plant-derived stress hormone) binds to and detaches mitochondria-bound HK from several cancer cell types (Goldin et al., 2008). HK detachment can be visualized using HK–GFP. Cell expression of HK-I–GFP showed punctuated distribution that co-localized with MitoTracker Red (**Figure 6**), suggesting a mitochondrial localization. The induction of apoptosis by STS resulted in HK–I-GFP detachment, as reflected by the diffuse fluorescence seen (**Figure 6**).

The small molecule alkylating agent, 3-bromopyruvic acid (3BP) was found to be phosphorylated by HK and to inhibit glycolytic rate in VX2 tumors (Ko et al., 2001). 3BP covalently modifies protein cysteine residues, resulting in rapid activity loss (Meloche and Monti, 1975; Ko and McFadden, 1990; Pereira da Silva et al., 2009). In animal models, 3BP showed high efficacy against advanced stage malignant tumors by inhibiting both glycolysis and mitochondrial energy generation, possibly by interfering with the HK–VDAC1 complex (Berridge et al., 2010). Thus, 3BP is proposed to target metabolism and blocks energy supplies. Other studies demonstrated the potential of 3BP as a potent anti-cancer agent in humans (Ko et al., 2012). Current knowledge related to 3BP and its promise as a future cancer therapeutic are the focus of a special 2012 issue of the Journal of Bioenergetics and Biomembranes (JOBB 44-1, 2012).

In conclusion, since VDAC1-bound HK is essential for tumor cells, conferring several advantages (see HK-linked Protection Against Cell Death is Mediated via Interaction with VDAC1), including protection against apoptotic events and promoting aerobic glycolysis, the detachment of HK from VDAC1 offers a novel therapeutic strategy to augment apoptosis, revert the hyper-glycolytic state and enhance the therapeutic efficacy of conventional chemotherapeutic agents.

### MEMBERS OF THE Bcl-2 FAMILY OF PROTEINS INTERACT WITH VDAC

The B cell lymphoma/leukemia-2 (Bcl-2) family of proteins plays an essential role in the control of apoptosis (Brown, 1997) at the interface with mitochondria. The Bcl-2 family consists of more than 20 pro-apoptotic (e.g., Bid, Bax, and Bak) and anti-apoptotic (e.g., Bcl-2 and Bcl-xL) members, all characterized by the presence of Bcl2 homology (BH) domains (Adams and Cory, 2007; Youle and Strasser, 2008). Accordingly, these proteins can be sub-divided into three main groups, based on the regions of the BH domain they contain and their function, namely the multi-domain anti-apoptotic (Bcl-2, Bcl-xL, Bcl-w, Mcl-1, and Bfl-1/A1), the multi-domain pro-apoptotic [Bcl-2-associated X protein (Bax) and Bak], and the BH3-only pro-apoptotic (Bid, Bim, Bad, Bik, Noxa, PUMA, Bmf, and Hrk) proteins (Certo et al., 2006). The anti-apoptotic members of the Bcl-2-family contribute to tumor initiation, disease progression, and drug resistance (Miyashita and Reed, 1993; Adams and Cory, 2007). Indeed, enhanced expression of anti-apoptotic Bcl-2 family members was shown to be associated with the resistance of many tumors to chemotherapy (Sentman et al., 1991; Adams and Cory, 2007). For example, in multiple studies, increased levels of Bcl-2, Bcl-xL, and Mcl-1 proteins were linked to survival of multiple myeloma cells and resistance to chemotherapy (Oancea et al., 2004), while in most cases of chronic lymphocytic leukemia (CLL), elevated Bcl-2 mRNA and protein levels are noted (Tsujimoto et al., 1984).

The mechanism whereby Bcl2 family members control apoptosis is still not fully understood. However, it is well established that their activities involve controlling OMM permeability (Scorrano et al., 2003; Adams and Cory, 2007; Youle and Strasser, 2008). Accumulated findings linked the activity of both anti-apoptotic and pro-apoptotic proteins to their association with VDAC (Shimizu and Tsujimoto, 2000; Shimizu et al., 2000b; Sugiyama et al., 2002; Shi et al., 2003a; Tsujimoto, 2003; Malia and Wagner, 2007; Tajeddine et al., 2008; Arbel and Shoshan-Barmatz, 2010). VDAC1 interacts with Bax, Bim, Bcl2, and Bcl-xL in isolated mitochondria and in reconstituted membrane systems (Shimizu et al., 2000b; Rostovtseva et al., 2004; Malia and Wagner, 2007; Arbel et al., 2012). Purified C-terminally truncated Bcl2 and Bcl-xL interact with VDAC1 and reduced the channel conductance of wild type but not of certain mutated forms of VDAC1 (Arbel and Shoshan-Barmatz, 2010; Arbel et al., 2012) or N-terminally truncated VDAC (Abu-Hamad et al., 2009; Arbel et al., 2012). Similarly, both Bcl2 and Bcl-xL prevented apoptosis as induced by various stimuli in cells expressing wild type but not mutated VDAC1 (Abu-Hamad et al., 2009; Arbel and Shoshan-Barmatz, 2010; Arbel et al., 2012). It was proposed that VDAC1 interacts with both Bax and Bcl-xL to form a tertiary complex (Shi et al., 2003a). A direct interaction between Bcl-xL and VDAC1 was demonstrated by NMR (Malia and Wagner, 2007). Interaction of the putative loop region of VDAC1 with Bcl-xL was proposed (Shi et al., 2003a). Bcl-xL was also shown to affect VDAC oligomerization, as revealed by chemical cross-linking of micelle-bound VDAC, shifting the equilibrium of VDAC from the trimeric to the dimeric state (Malia and Wagner, 2007). Furthermore, synthetic peptides corresponding to the VDAC1 N-terminal region and other cytosol-facing VDAC1 sequences bound Bcl2 and Bcl-xL (Shi et al., 2003a; Abu-Hamad et al., 2009; Shoshan-Barmatz et al., 2009; Arbel and Shoshan-Barmatz, 2010; Arbel et al., 2012). Such peptides, in cell-penetrating form or expressed in the cell, were able to suppress the anti-apoptotic activities of Bcl2 and Bcl-xL in cells (Abu-Hamad et al., 2009; Arbel and Shoshan-Barmatz, 2010; Arbel et al., 2012). Thus, VDAC1-based peptides, targeting the anti-apoptotic activity of Bcl-xL and Bcl2, can serve as potential cancer therapeutics.

Bax and VDAC reconstituted into liposomes were shown to form a new channel, with a conductance 4–10 times larger than that of the individual proteins (Shimizu et al., 2000b), and with such increase being prevented by Bcl-xL. The interaction of Bax and Bim with VDAC leads to Cyto *c* release, with Bcl-xL and anti-VDAC antibodies preventing this release (Shimizu et al., 2001). It has also been shown that Bid but not Bax modulates VDAC channel conductance (Rostovtseva et al., 2004).

In VDAC1-depleted cells, cisplatin-induced activation of Bax was inhibited (Tajeddine et al., 2008), suggesting that VDAC1 is involved in Bax-mediated apoptosis. Furthermore, Bcl-2 and Bcl-xL blocked As_2_O_3_-induced VDAC dimerization (Zheng et al., 2004). These results indicate that Bcl-2 family proteins regulate VDAC-mediated apoptosis and hence, the release of apoptogenic proteins from mitochondria.

### VDAC AND THE TRANSLOCATOR PROTEIN

Translocator protein is an 18-kDa integral membrane protein, originally identified as the peripheral benzodiazepine receptor or isoquinoline-binding protein (Korkhov et al., 2010), that is over-expressed in breast, prostate, colon, and brain cancer, with protein expression linked to cancer progression and poor survival rates (Batarseh and Papadopoulos, 2010). Like VDAC1, TSPO is located in the OMM and was proposed to activate PTP opening, leading to apoptosis (Levin et al., 2005; Veenman et al., 2007; Sileikyte et al., 2011). Interactions between TSPO and VDAC are thought to play a role in apoptosis (Gavish et al., 1999; Levin et al., 2005; Veenman et al., 2007). High-affinity ligand occupancy of TSPO was shown to attenuate anti-neoplastic agent-induced apoptosis (Kugler et al., 2008). The observations that TSPO is involved in the generation of ROS and that TSPO association with VDAC may enhance ROS production prior to apoptotic induction, indirectly links both proteins (Golani et al., 2001; Veenman et al., 2002, 2007). Still, there is no direct evidence for a TSPO–VDAC1 interaction at the molecular level.

### INTERACTION OF VDAC1 WITH AND REGULATION BY CYTOSKELETAL PROTEINS

In cancer, morphogenic changes are required for high proliferative rates, the acquisition of inappropriate migratory patterns, and invasive characteristics (Hall, 2009). Alterations in the dynamics of the actin and tubulin components of the cytoskeleton are well-known consequences of signaling and the triggering of responses like apoptosis (Franklin-Tong and Gourlay, 2008; Rostovtseva and Bezrukov, 2012). Mitochondria are dynamic organelles that actively travel along the cytoskeleton within the cell. Within this framework, specific association of VDAC1 with tubulin was shown by co-immunoprecipitation (Carre et al., 2002) and by tubulin-induced VDAC1 closure (Rostovtseva et al., 2008a). A model was proposed whereby the negatively charged C-terminus of tubulin penetrates into the positively charged VDAC1 pore, leading to decreased channel conductance (Rostovtseva et al., 2008b). It was proposed that tubulin, VDAC1 and mitochondrial creatine kinase (MtCK) are members of a super complex that is structurally and functionally coupled to the ATP synthasome (Saks et al., 2010). Recent evidence indicates that the lipid composition of the membrane is capable of controlling tubulin–VDAC interactions, thus tuning VDAC sensitivity to blockage by tubulin (Rostovtseva et al., 2012). Similarly, it has been reported that tubulin–VDAC interactions can be regulated by varying the membrane surface charge (Gurnev et al., 2012). In a cellular model of cardiomyocyte physiology, the super complex presents a different composition, namely one in which tubulin is replaced by HK and MtCK is absent, infusing mitochondrially generated ATP into the glycolytic pathway and thereby contributing to the Warburg effect (Saks et al., 2010). Other proteins associated with cytoskeleton dynamics, such as gelsolin (Kusano et al., 2000), adseverin or scinderin (SCIN; Miura et al., 2012), have been reported to associate with VDAC, with concomitant anti-apoptotic effects.

All of these VDAC1–protein interactions establish a new interplay between kinase signaling pathways, mitochondrial respiration, and the highly dynamic microtubule network characteristic of cancerogenesis and cell proliferation. VDAC1, as the common denominator in all the interactions described above, thus presents a single unique target for efforts addressing cell physiological, metabolic, and morphological aspects of cancer.

### INTERACTION OF VDAC1 WITH VIRAL PROTEINS

Many viruses encode for proteins acting on mitochondria, in general, on VDAC1, in particular, or directly targeting proteins in the apoptotic pathway (Boya et al., 2001, 2003; Everett and McFadden, 2001; Irusta et al., 2003; Verrier et al., 2003). Other viruses influence apoptosis by mimicking Bcl-2 family proteins, changing their expression levels or directly interacting with Bcl-2 family proteins (Halestrap et al., 2002; Boya et al., 2004). HIV-1 offers an excellent example of neuronal and immunological-destructive apoptosis involving mitochondria (Sui et al., 2004; Perfettini et al., 2005). HIV-1 envelope proteins can trigger mitochondrial apoptosis, increasing cytosolic Ca^2+^, ROS, and transcriptional activation of p53, thus increasing the expression of pro-apoptotic proteins, such as Bax (Castedo et al., 2003). The PB1-F2 protein of the influenza A virus, when localized in mitochondria, induces alteration of mitochondrial morphology, dissipation of the mitochondrial membrane potential, and cell death and has been reported to interact directly with VDAC (Zamarin et al., 2005; Danishuddin et al., 2009). Hepatitis B virus (HBV) is another mitochondria-interacting virus (Boya et al., 2003, 2004; Shirakata and Koike, 2003). The HBx protein is highly homologous to VDAC1 and is proposed to “poison” the homo-oligomeric state of VDAC1, creating hetero-oligomeric structures that induce permeability of the mitochondria, allowing Cyto *c* release (Stanley et al., 1995; Rahmani et al., 2000; Shoshan-Barmatz et al., 2006; Goncalves et al., 2007; Hoogenboom et al., 2007). This action of HBx can result in the development of hepatocellular carcinomas (Rahmani et al., 2000). In infected liver cell lines, hepatic virus (HEV) not only induced up-regulation of VDAC expression in infected human hepatoma cell lines but also encouraged higher levels of VDAC oligomerization, leading to apoptosis (Moin et al., 2007).

## VDAC1 EXPRESSION LEVELS IN HEALTHY AND CANCER CELLS

In higher eukaryotes, VDAC1 is the most predominant isoform expressed. Several studies have shown that alterations in VDAC1 expression affect both the ability of cells to generate energy and their sensitivity to apoptosis-inducing reagents.

### VDAC EXPRESSION LEVELS IN CANCERS AND ENHANCEMENT BY PRO-APOPTOTIC DRUGS

Several studies have demonstrated remarkable differences in the expression levels of VDACs between tumor cells and normal tissues. VDAC1 expression in several cancer cell lines was higher than in the control fibroblast cell line (Simamura et al., 2006, 2008). NSCLC cells specifically exhibited high expression levels of VDAC1. This was shown to directly correlate with poor outcome in early stage NSCLC, thus establishing an association with aggressive tumor behavior (Grills et al., 2011). In addition, in several melanoma and prostate cancer cell lines, a correlation between levels of VDAC1 expression and induction of Cyto *c* release by G3139 was demonstrated (Lai et al., 2006). In a proteomic analysis of aging-related proteins in human normal colon epithelial tissues, significant up-regulation of 19 proteins was noted, including VDAC1 and VDAC2 (Li et al., 2007).

Several cancer treatments were found to enhance expression of VDAC. Up-regulation of VDAC1 expression was observed in three different acute lymphoblastic leukemia (ALL) cell lines (697, Sup-B15, and RS4;11) following prednisolone treatment, an observation that can be explored for predicting eventual outcome in childhood ALL (Jiang et al., 2011). VDAC1 over-expression was observed in a cisplatin-sensitive cervix squamous cell carcinoma cell line (A431) when exposed to cisplatin, while in a cisplatin-resistant cell line (A431/Pt), the treatment resulted in down-regulation of VDAC1 (Castagna et al., 2004). Up-regulation of VDAC has been reported upon UV irradiation of apoptosis-sensitive cells (Voehringer et al., 2000). When A375 human malignant melanoma cells were treated with the tyrosinase inhibitor, arbutin, a potential anti-tumor agent (Nawarak et al., 2009), VDAC1 expression level was found to be up-regulated (Cheng et al., 2007). In addition, ROS was found to induce up-regulation of VDAC that could be prevented by the ROS chelator, epigallocatechin (Jung et al., 2007).

The relationship between VDAC1 expression levels and sensitivity to various treatments was presented in several studies. The PC3 and DU145 prostate cancer cell lines are relatively resistance to apoptosis as induced by G3139 and were found to express less VDAC than did G3139-sensitive LNCaP cells (Lai et al., 2006). Similarly, reducing VDAC1 expression by siRNA efficiently prevented cisplatin-induced apoptosis and Bax activation in NSCLC (Tajeddine et al., 2008), attenuated endostatin-induced apoptosis (Yuan et al., 2008) and inhibited selenite-induced PTP opening (Tomasello et al., 2009). The anti-cancer activity of furanonaphthoquinones (FNQs) was increased upon VDAC1 over-expression and decreased upon silencing of VDAC1 expression by siRNA (Simamura et al., 2008).

Finally, it has also been shown that the CD45 expression is accompanied by elevated VDAC1 expression in myeloma cells sensitized to a diverse set of apoptotic stimuli via the mitochondrial pathways (Liu et al., 2006).

### SILENCING OF VDAC1 EXPRESSION

Among the complex array of genetic changes accompanying cancer development, all cancerous cells develop altered metabolism, with VDAC1 and HK playing important functions in this metabolic reprograming (see The Interaction of VDAC1 with Hexokinase Regulates Cell Bioenergetics and Apoptosis). VDAC1 contributes to cancer metabolism via transport of various metabolites, mediating ATP/ADP exchange across the OMM and by the binding and channeling of mitochondrial ATP directly to HK (see Cancer Metabolism, Hexokinase and VDAC). The importance of VDAC1 for cancer cells is further reflected in the findings that silencing VDAC1 expression reduced cellular ATP levels and cell growth (Abu-Hamad et al., 2006). Furthermore, when HeLa cervical cancer cells stably expressing shRNA directed against hVDAC1 were injected into nude mice, the development of a solid tumor was inhibited (Koren et al., 2010). It has also been shown that VDAC1 silencing potentiates H_2_O_2_-induced apoptosis and impairs mitochondrial Ca^2+^ loading, while silencing VDAC2 had the opposite effects (De Stefani et al., 2012). Together, these findings indicate that over-expression of VDAC1 in cancer cells could contribute to the high glycolytic phenotype seen.

### ENHANCEMENT OF VDAC1 EXPRESSION, OLIGOMERIZATION AND APOPTOSIS INDUCTION

As presented above (see VDAC Expression Levels in Cancers and Enhancement by Pro-apoptotic Drugs), the expression level of VDAC1 serves as a crucial factor in the process of mitochondria-mediated apoptosis as induced by various means. Indeed, over-expression of VDAC from different sources, such as yeast, rice, fish, mice, and humans, was found to induce apoptosis (Godbole et al., 2003; Zaid et al., 2005; Ghosh et al., 2007; Lü et al., 2007). Over-expression of murine (m) VDAC1 or rat (r) VDAC1 in U-937 cells resulted in apoptotic cell death (Zaid et al., 2005). Moreover, over-expression of rice VDAC induced apoptosis (Godbole et al., 2003) that was blocked by Bcl-2 and the VDAC channel inhibitor, DIDS. Similar results were obtained following transfection of fish cells to express *Paralichthys olivaceus* VDAC (Lü et al., 2007). Over-expression of VDAC1 causes depolarization of the IMM and activation of the PTP (De Pinto et al., 2007; Tomasello et al., 2009).

The mechanism underlying cell death induced by VDAC1 over-expression is not fully understood. However, recent studies demonstrated that VDAC1 over-expression is accompanying by its oligomerization (Zalk et al., 2005; Goncalves et al., 2007; Hoogenboom et al., 2007; Malia and Wagner, 2007; Shoshan-Barmatz et al., 2008a,b; Zeth et al., 2008; Keinan et al., 2010). VDAC1 has been shown to form different oligomeric states, namely monomers, dimers, trimers, tetramers, hexamers, and higher-order oligomers (see VDAC1 Oligomerization and Function). Moreover, the supra-molecular assembly of VDAC1 in cultured cells is highly enhanced upon apoptosis induction, supporting the involvement of VDAC1 oligomerization in Cyto *c* release and thus, in apoptosis (Shoshan-Barmatz et al., 2008b; Keinan et al., 2010). Moreover, VDAC1 over-expression resulted in its oligomerization in the absence of any apoptosis stimuli (Shoshan-Barmatz et al., 2008b). It should be noted that apoptosis induced by VDAC1 over-expression shares common features with apoptosis induced by other stimuli (e.g., STS, H_2_O_2_), as is the case for inhibition by RuR, Bcl2, HK-I over-expression, or DIDS, all agents that interact with VDAC1 (Shoshan-Barmatz et al., 2006). Finally, HEV-infected human hepatoma cells showed enhanced VDAC expression, leading to VDAC oligomerization and apoptosis (Moin et al., 2007).

Thus, the over-expression of VDAC1, as induced by drugs, such as arbutin, prednisolone, or cisplatin, or by viral proteins or UV irradiation, as well as the correlation between drug effectiveness and VDAC expression, suggest that the anti-cancer activities of these drugs is associated with VDAC levels. This is further supported by the finding that cisplatin-induced apoptosis is inhibited in cells silenced for VDAC1 expression (De Pinto et al., 2007; Tomasello et al., 2009). In addition, endostatin-induced apoptosis was decreased upon silencing VDAC1 expression, and enhanced by over-expression of VDAC1 (Yuan et al., 2008). We thus propose that the high levels of VDAC1 promote its oligomerization, leading to apoptosis (Shoshan-Barmatz et al., 2008b; Keinan et al., 2010). Accordingly, anti-cancer strategies that specifically up-regulate VDAC1 levels in cancer cells may activate the mitochondrial apoptotic pathway, with concomitant benefit to the patient.

## VDAC1 FUNCTION IN ROS RELEASE, ROS-MEDIATED APOPTOSIS AND INTERACTION WITH NO

### MITOCHONDRIA, ROS, AND OXIDATIVE STRESS

Reactive oxygen species serve important roles in diverse events, such as cellular proliferation, differentiation, and migration. It is now accepted that cellular “redox” signaling is involved in regulating normal processes and disease progression, including angio- genesis, oxidative stress, aging, and cancer. Cellular levels of ROS are linked to anti-tumor immunity, the oxidative tumor microenvironment, proliferation, and death of cancer cells (Manda et al., 2009).

Cancer cells exhibit high basal levels of oxidative stress due to the activation of oncogenes, loss of tumor suppressors, and the effects of the tumor microenvironment (Cairns et al., 2011). Hypoxia, a characteristic of most solid tumor microenvironments, causes a progressive elevation in mitochondrial ROS production (chronic ROS) which over time leads to the stabilization of cells via increased HIF-2α expression, which serve as signaling molecules that activate the transcription of genes involved in cellular hypoxic adaptation (Hamanaka and Chandel, 2009). It is becoming apparent that hypoxia enables cells to survive with sustained levels of elevated ROS. However, the increased oxidant concentrations associated with cellular transformation promote tumorigenicity through signaling but can also damage DNA, proteins, and lipids. Indeed, promoting oxidative stress in cancer cells selectively kills several cancer cell lines (Trachootham et al., 2006; Nogueira et al., 2008; Ralph et al., 2010).

Reactive oxygen species are mainly produced in the mitochondria as by-products of respiratory chain reactions, with 1–5% of the oxygen consumed by mitochondria in human cells being converted to ROS, such as superoxide anions (${\text{O}}_{2}^{-\cdot }$), H_2_O_2_, and hydroxyl radicals (Murphy, 2009). Oxidative stress results when production of ROS exceeds the capacity of mitochondrial and cellular anti-oxidant defenses to remove these toxic species. Indeed, to combat harmful ROS, cells possess several anti-oxidant defense mechanisms, including catalytic removal of reactive species by enzymes like superoxide dismutase (SOD), catalase, and peroxidase (Barber et al., 2006; **Figure 6**). However, about 1% of the ROS escapes elimination and cause oxidative cellular damage. The rules governing ROS transport via the OMM and control of this process, as well as the molecular mechanism of ROS activating VDAC to release Cyto *c*, are not clear.

### VDAC1 MEDIATES ROS RELEASE FROM THE MITOCHONDRIA

Since ROS are generated in the mitochondria, they can attack the mitochondria directly and, when released to the cytosol, can attack and modify DNA, lipids, and proteins, thus affecting cell survival (Ott et al., 2007). This requires ROS release from the mitochondria to the cytosol, namely, crossing the OMM. VDAC1 in the OMM may serve as the major gateway for molecules exiting the IMS. Indeed, VDAC1 has been proposed to mediate ROS release from the IMS to the cytosol (**Figure 6**). Several pieces of evidence support the involvement of VDAC1 in ROS release from the IMS to the cytosol, including:

(i) HK-I and HK-II bound to VDAC1 decrease ROS release from mitochondria when over-expressed in HEK cells (Sun et al., 2008), thereby reducing intracellular levels of ROS (Ahmad et al., 2002; da-Silva et al., 2004). Also, expression of HK-I or HK-II was found to protect against oxidant-induced cell death (Ahmad et al., 2002; Bryson et al., 2002). Thus, detachment of HK from VDAC1 could lead to increased ROS generation and release to the cytoplasm, thereby activating cell death.

(ii) Closure of VDAC appears to impede the efflux of superoxide anions from the IMS, resulting in an increased steady-state level of ${\text{O}}_{2}^{-\cdot }$, causing internal oxidative stress and sensitizing mitochondria toward the Ca^2+^-induced MPT (Tikunov et al., 2010).

(iii) The VDAC inhibitors, DIDS and dextran sulfate, inhibited ${\text{O}}_{2}^{\cdot }$ release from the mitochondria to the cytosol (Han et al., 2003).

### ROS-INDUCED APOPTOSIS IS MEDIATED VIA VDAC1

**FIGURE 7:**
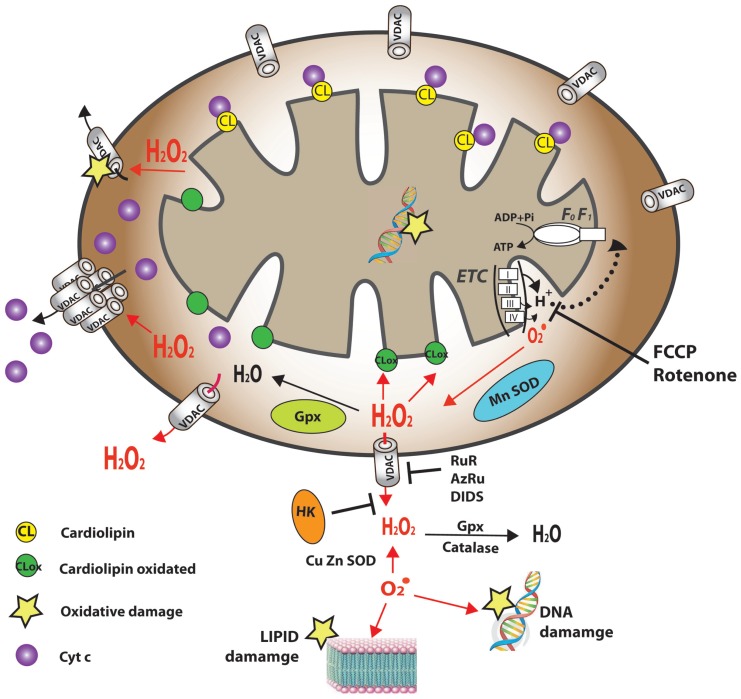
**Reactive oxygen species occlusion and degradation in mitochondria can prevent cell damage.** A schematic presentation summarizing current knowledge and proposed control mechanisms for ROS production, neutralization, and cellular effects (see VDAC1 Function in ROS Release, ROS-mediated Apoptosis and Interaction with NO). Free radicals generated by the electron transport chain are generally regarded as toxic metabolites and, as such, are degraded by specialized enzymes, namely catalases, peroxidases, superoxide dismutases, and glutathione peroxidase. Mitochondrial ROS cross the OMM via VDAC. The ROS that escapes catalytic removal can cause oxidative damage (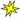) to mitochondria, and when released from the mitochondria, can damage cellular proteins, lipids, and DNA. In addition, ROS can induce apoptosis. ROS is proposed to induce Cyto *c* release by oxidizing the mitochondria-specific phospholipid, cardiolipin (CL). ROS-oxidized CL has a markedly lower affinity for Cyto *c*, thus rendering Cyto *c* free in the inter-membrane space. ROS can also induce VDAC oligomerization to yield a mega-channel mediating Cyto *c* release. VDAC inhibitors (e.g., RuR, AzRu, DIDS) and hexokinase (HK) can prevent ROS release to the cytosol. CuZnSOD, cytosolic (copper–zinc-containing) superoxide dismutases; MnSOD, mitochondrial (manganese-containing) superoxide dismutases; GPx, glutathione peroxidase.

Mitochondria are the major source of ROS that, when released to the cytosol, can damage proteins, lipids and DNA. ROS release is mediated via VDAC1, with its interactors (e.g., HK, DIDS, RuR, AzRu) inhibiting release (**Figure 7**). Mitochondria also contain several enzymes that can decompose ROS. ROS can promote Cyto *c* release from mitochondria (Petrosillo et al., 2001, 2003). In fact, it has been shown that apoptosis-inducing agents, such as inorganic arsenic compounds (Shi et al., 2004; Ding et al., 2005) and doxorubicin (Olson et al., 1981; Sokolove, 1994), cause oxidative damage to DNA and protein by inducing ROS generation.

Inhibition of ${\text{O}}_{2}^{-\cdot }$-induced apoptosis by DIDS, an inhibitor of VDAC channel activity, or anti-VDAC1 antibodies (Shoshan-Barmatz et al., 1996; Madesh and Hajnoczky, 2001; Shimizu et al., 2001; Simamura et al., 2006), suggests that ${\text{O}}_{2}^{-\cdot }$-induces Cyto *c* release via VDAC-dependent permeabilization of the OMM (Madesh and Hajnoczky, 2001). Moreover, ${\text{O}}_{2}^{-\cdot }$ was found to evoke Cyto *c* release in VDAC-reconstituted liposomes (Madesh and Hajnoczky, 2001). In other studies, it was found that ROS-induced alterations of VDAC1 and/or ANT can induce MMP selective for Cyto *c* release, without causing further mitochondrial damage (Madesh and Hajnoczky, 2001; Le Bras et al., 2005). In addition, ROS-induced up-regulation of VDAC1 can be prevented by the ROS chelator, epigallocatechin (Jung et al., 2007). It has been suggested that ROS-mediated Cyto *c* and SOD1 release from mitochondria involves VDAC, leading to increased susceptibility of mitochondria to oxidative stress and apoptosis (Li and Yuan, 2008).

Interactions between TSPO and VDAC1 are proposed to play a role in apoptosis (Gavish et al., 1999; Veenman et al., 2004; Levin et al., 2005; see VDAC and the Translocator Protein). TSPO appears to be involved in the generation of ROS, leading to apoptosis induction (Veenman et al., 2008). It was hypothesized that the close association of TSPO with VDAC may enhance the concentration of ROS generated by TSPO, leading to apoptosis induction (Golani et al., 2001; Veenman et al., 2002, 2007).

### NITRIC OXIDE, MITOCHONDRIA, VDAC AND CELL DEATH

Nitric oxide (NO) possesses both pro- and anti-apoptotic effects, depending on both cell type and NO concentration (Brune et al., 1999; Kim et al., 1999). Specific NO molecular targets include inhibition of Bcl-2 cleavage (Kim et al., 1998) and inactivation of caspases by *S*-nitrosylation (Li et al., 1999). At low physiological release rates, NO was found to inhibit PTP opening in a reversible manner*, *while at high physiological release rates, NO accelerated PTP opening (Brookes et al., 2000). VDAC1 was found to bind the endothelial NO synthase (eNOS; Sun and Liao, 2002). *In vitro* binding studies using glutathione *S*-transferase (GST)-tagged VDAC1 indicated that VDAC1 binds directly to eNOS and that this interaction amplified eNOS activity. Furthermore, the calcium ionophores, A23187 and bradykinin, both known to activate eNOS, increased VDAC1–eNOS complex formation, suggesting a potential role for intracellular Ca^2+^ in mediating this interaction (Sun and Liao, 2002). The above results indicate that the interaction between VDAC and eNOS may be important for regulating eNOS activity and modulation of VDAC by NO (Sun and Liao, 2002). Recently, it has been reported that exogenous NO directly interacts with purified VDAC from rat heart and inhibited its channel activity in a biphasic manner. This inhibition was prevented by a NO scavenger. Channel activity inhibition paralleled the delay in opening of the cardiac PTP by NO (Cheng et al., 2011).

## VDAC1 TRANSPORT OF Ca^2+^ AND FUNCTION IN ER–MITOCHONDRIA CROSS-TALK

### MITOCHONDRIAL Ca^2+^ TRANSPORT AND VDAC1

Ca^2+^ is a ubiquitous cellular signal, with changes in intracellular Ca^2+^ concentration not only stimulating a number of intercellular events but also triggering cell death pathways, including apoptosis. Ca^2+^ signals result in the regulation of Ca^2+^-dependent enzymes, such as phospholipases, proteases, and nucleases, important during injury or cell death (Berridge et al., 2000). Apart from their metabolic and apoptotic roles, mitochondria are also a major hub of cellular Ca^2+^ homeostasis. Indeed, mitochondrial Ca^2+^ homeostasis is fundamental for a wide range of cellular activities, such as control of oxidative phosphorylation (Cox and Matlib, 1993; Maack et al., 2006), modulation of cytosolic calcium signals (Gunter et al., 2000), cell death (Giacomello et al., 2007), and secretion (Maechler et al., 1997; Lee et al., 2003). Intra-mitochondrial Ca^2+^ controls energy metabolism by enhancing the rate of NADH production by modulating critical enzymes, such as those of the TCA cycle and fatty acid oxidation (Nichols and Denton, 1995; Denton, 2009), linking glycolysis to the TCA cycle (Cárdenas et al., 2010).

Ca^2+^ transport across the IMM is mediated by several recently identified proteins. The mitochondrial Ca^2+^ uniporter (MCU; Baughman et al., 2011; De Stefani et al., 2011), as well as its regulatory protein, the EF hand-containing protein termed MICU1 (for mitochondrial calcium uptake 1), acting as a Ca^2+^ sensor that controls mitochondrial Ca^2+^ entry mediated by the MCU, were identified (Santo-Domingo and Demaurex, 2010). The high-affinity mitochondrial Ca^2+^/H^+^ exchanger, Letm1, is able to import Ca^2+^ at low (i.e., sub-micromolar) cytosolic concentrations into energized mitochondria (Jiang et al., 2009), while the Na^+^/Ca^2+^ exchanger superfamily, NCLX, is the major Ca^2+^ efflux mechanism (Palty et al., 2010). All the Ca^2+^ transport systems described above mediate the transport of Ca^2+^ across the IMM (**Figure 1**). However, incoming Ca^2+^ must first traverse the OMM. The only identified protein mediating Ca^2+^ transport in the OMM is VDAC1. Indeed, VDAC1 is permeable to Ca^2+^ and possesses Ca^2+^-binding sites (Gincel et al., 2001; Rapizzi et al., 2002; Tan and Colombini, 2007) and it has been shown that control of OMM Ca^2+^ permeability is mediated by VDAC (Bathori et al., 2006; Tan and Colombini, 2007).

### Ca^2+^, MITOCHONDRIA, APOPTOSIS, AND VDAC

Although changes in mitochondrial Ca^2+^ concentration are known to trigger apoptosis, the precise mechanism is not known.

VDAC1 is highly Ca^2+^-permeable and modulates Ca^2+^ access to the mitochondrial inter-membrane space (Gincel et al., 2001; Tan and Colombini, 2007). Non-physiological Ca^2+^ overload depolarizes mitochondria by opening the PTP, with concomitant release of Cyto *c* and other IMS proteins (Crompton, 1999; Baumgartner et al., 2009) that can lead to both apoptotic and necrotic cell death, associated with disease pathogenesis (Rasola and Bernardi, 2011). Thus, Ca^2+^ overload disrupts energy production by mitochondria and enables apoptosis and/or necrosis (Shoshan-Barmatz and Gincel, 2003; Kinnally et al., 2011; Rasola and Bernardi, 2011). Ca^2+^ overload also results in production of ROS. To date, the mitochondrial target for Ca^2+^ activation of Cyto *c* release has not been identified. The inhibition of PTP opening by RuR (Gincel et al., 2001), while protection against cell death induced by several apoptotic stimuli (Baek et al., 1997; Ding et al., 2001; Bae et al., 2003) may arise from RuR interaction with VDAC1, inducing channel closure (Gincel et al., 2001). This is supported by the findings that E72Q-VDAC1 did not interact with RuR and cells expressing this mutant were not protected against apoptosis by RuR (Zaid et al., 2005). These observations suggest that Ca^2+^-binding sites in VDAC1 (Israelson et al., 2008) serve regulatory functions, including those involved in apoptosis induction.

### ER–MITOCHONDRIA Ca^2+^ TRANSPORT, VDAC, AND APOPTOSIS

The ER is a dynamic reservoir of Ca^2+^ ions with electrical or chemical cell stimulation resulting in its activation (Bootman et al., 2002; Verkhratsky and Petersen, 2002), highlighting the role of this organelle as an indispensable component of Ca^2+^ signaling processes. Ca^2+^ originating from the ER has been shown to be a potent death-inducing signal (Pinton et al., 2008).

The interplay between ER- and mitochondria-mediated Ca^2+^ signaling is a determinant of cellular fate through the control of apoptosis and autophagy (Decuypere et al., 2011). Release of Ca^2+^ from the ER via inositol-1,4,5-trisphosphate receptors (IP_3_Rs) has been observed in models of apoptosis and has been implicated directly in mitochondrial Ca^2+^ overload (Hajnoczky et al., 2003). The specific sites of physical association between the ER and mitochondria, known as mitochondria-associated membranes (MAMs), include VDAC among the proteins present at these junctions (Bononi et al., 2012). The mitochondrial chaperone, grp75 (glucose-regulated protein 75), has been proposed to mediate the molecular interaction of VDAC1 with the ER Ca^2+^-release channel, IP_3_R, and that chaperone-mediated conformational coupling of these proteins enables better transfer of the Ca^2+^ ions from the ER to the mitochondria (Szabadkai et al., 2006). In a cell-free system containing purified mitochondria, ER vesicles, IP_3_R, and VDAC1, PTP activity was identified as being involved in the ER-triggered pro-apoptotic mitochondrial membrane permeabilization process (Deniaud et al., 2008). It has been recently established that VDAC1 (but not isoforms 2 or 3) selectively interacts with IP_3_Rs and is preferentially involved in the transmission of low-amplitude apoptotic Ca^2+^ signals to mitochondria (De Stefani et al., 2012). The involvement of VDAC1 in ER–mitochondria Ca^2+^ cross-talk places VDAC1 at a crucial position on the route transferring Ca^2+^ signals from the ER to mitochondria, and thus couples ER and mitochondrial functions (De Stefani et al., 2012).

## VDAC AS A PHARMACOLOGICAL TARGET FOR COMPOUNDS AFFECTING CELL PROLIFERATION OR APOPTOSIS

VDAC1, as a multi-functional channel involved both in metabolic homeostasis and apoptosis, can be considered as a prime target for therapeutic agents designed to act via metabolic interference or modulating apoptosis (Shoshan-Barmatz and Ben-Hail, 2011; Shoshan-Barmatz and Golan, 2012). Indeed, several studies identified pharmacological agents that target VDAC as inducing cancer cell death. These agents can be categorized into the following groups:

### ROS-PRODUCING VDAC-DEPENDENT AGENTS

Reactive oxygen species has been shown to promote Cyto *c* release from mitochondria, thereby inducing apoptotic cell death (Petrosillo et al., 2001; Petrosillo et al., 2003) (see VDAC1 Function in ROS Release, ROS-mediated Apoptosis and Interaction with NO). Currently known drugs acting via enhancing ROS production include FNQs, erastin, and As_2_O_3_.

Furanonaphthoquinones were proposed to induce caspase-dependent apoptosis via the production of NADH-dependent ROS. In HeLa cells, FNQs induce mitochondrial swelling, with subsequent apoptosis, while radical scavengers and anti-VDAC1 antibodies reduced this effect (Simamura et al., 2008), suggesting that FNQ-mediated apoptosis is VDAC-dependent. This claim is further supported by the findings that the ROS production and the anti-cancer activity of FNQs were increased upon VDAC1 over-expression and decreased upon silencing of VDAC1 expression by siRNA (Simamura et al., 2008). FQNs were found to also up-regulate the pro-apoptotic proteins, Bad, Bax, and Cyto *c*, while down-regulating the anti-apoptotic proteins, Bcl-2, survivin, and XIAP in A549 cells (Su et al., 2010).

Erastin is a cell-permeable piperazinyl–quinazolinone compound that exhibits oncogene-selective lethality toward cells harboring activating mutations in the RAS–RAF–MEK signaling pathway. Erastin treatment in these cells rapidly induces ROS production and a non-apoptotic form of cell death (Yagoda et al., 2007). Knockdown of *vdac2 *or *vdac3* caused resistance to erastin, suggesting their involvement in the mechanism of action of erastin (Yagoda et al., 2007). It was also reported that RSL5, a compound that has increased lethality in the presence of oncogenic RAS, and erastin induced VDAC3-dependent cell death (Yang and Stockwell, 2008). Finally, the erastin analog, PRLX 93936, was found to bind VDAC2 and VDAC3 using a proteomics approach (Sahasrabudhe et al., 2008). PRLX 93936 is currently in clinical Phase I for treatment of solid tumors.

As_2_O_3_, a clinically active anti-leukemia agent reported to inhibit mitochondrial respiratory function, increases ROS generation, and enhances the activity of other O_2_^.-^ producing agents against cultured leukemia cells and primary leukemia cells isolated from patients (Pelicano et al., 2003).

### CHEMICALS THAT DIRECTLY INTERACT WITH AND MODIFY VDAC ACTIVITY

Various compounds interact with VDAC to modify its activity (**Table 2**). *Acrolein* (2-propen-1-al), the most reactive of the α,β-unsaturated aldehydes and a toxic compound (Adams and Klaidman, 1993) that was proposed to react with DNA and proteins, preferential binds at p53 mutational hotspots to inhibit DNA repair (Feng et al., 2006). Acrolein is a respiratory irritant that is generated during cooking and is abundant in the environment (Izard and Libermann, 1978). The compound is also endogenously generated by lipid peroxidation at sites of injury (Adams and Klaidman, 1993). In pathological conditions associated with oxidative stress (Voulgaridou et al., 2011), like spinal cord injury (Luo et al., 2005) and Alzheimer’s disease (Dang et al., 2010), levels of acrolein were found to be elevated. Acrolein is reported to enhance carbonylation of VDAC, an event that was found to be significantly increased in the brains of Alzheimer’s disease patients (Mello et al., 2007).

**Table 2:** Reagents proposed to act via VDAC (for details, see VDAC as a Pharmacological Target for Compounds Affecting Cell Proliferation or Apoptosis).

Agents	VDAC-mediated cytotoxic	Reference
Avicins	Pro-apoptotic plant stress metabolites, target and close VDAC	Haridas et al. (2007)
Fluoxetine (Prozac)	Anti-depressant induces cell proliferation and cell-line dependent apoptotic modulator. Interacts directly with VDAC, inhibits PTP opening and apoptosis	Nahon et al. (2005)
Cisplatin	Interacts directly with VDAC1 and inhibits PTP opening	Castagna et al. (2004), Yang et al. (2006)
Acrolein	α,β-Unsaturated aldehydes, carbonylates VDAC in Alzheimer’s disease	Mello et al. (2007)
Erastin	Anti-cancer agent that binds directly to VDAC2 to induce cell death	Yagoda et al. (2007)
Endostatin	Small collagen type XVIII fragment, angiogenesis inhibitor. Interacts with VDAC1, inducing PTP opening	Yuan et al. (2008)
Oblimersen	18-mer phosphorothioate anti-sense oligonucleotide. Binds directly to VDAC, blocks channel activity, pro-apoptotic	Tan et al. (2007)
Furanonaphthoquinones	Induce caspase-dependent apoptosis. FNQ-mediated apoptosis is VDAC-dependent	Simamura et al. (2008)
Geldanamycin 17AAG	Binds to VDAC and inhibits cell invasion	Xie et al. (2011)

*Avicins* represents a family of triterpenoid saponins, plant stress metabolites that exhibit cytotoxic activity in tumor cells. These compounds possess anti-inflammatory and anti-oxidant properties capable of perturbing mitochondrial function and initiating apoptosis in tumor cells (Gaikwad et al., 2005; Haridas et al., 2005). Avicin G reduces VDAC1 channel conductance, ATP levels, and respiration rates in Jurkat cells (Lemeshko et al., 2006; Haridas et al., 2007).

*Cisplatin* (*cis*-diamminedichloro platinum(II)) is a widely used anti-cancer drug that trigger apoptosis (Santin et al., 2011). Cisplatin acts by forming inter- and intra-strand nuclear DNA cross-links upon binding (Zamarin et al., 2005). Mitochondria, however, have been implicated as a cisplatin target, leading to apoptosis induction (Yang et al., 2006; Cullen et al., 2007). Cisplatin binds to mitochondrial DNA and VDAC (Yang et al., 2006) to regulate VDAC activity (Castagna et al., 2004). Thus, it was proposed that VDAC acts as a cisplatin receptor during apoptosis (Thinnes, 2009). Recently it was suggested that over-expression of SCIN, a calcium-dependent actin-binding protein, is a novel cisplatin-resistant marker in the human bladder cancer cell line, HT1376 (Miura et al., 2012). It was proposed that the high expression of SCIN and its interaction with VDAC mediates resistance to cisplatin (Miura et al., 2012).

*Endostatin* is a naturally occurring 20 kDa C-terminal fragment derived from type XVIII collagen that may interfere with the pro-angiogenic action of growth factors (Folkman, 2006). Endostatin has a wide range of anti-cancer spectrum targets (Karamouzis and Moschos, 2009). Decreasing VDAC1 levels by siRNA attenuates endostatin-induced apoptosis, while over-expression of VDAC1 enhances the sensitivity of endothelial cells to endostatin (Yuan et al., 2008). Endostatin was found to induce PTP opening through VDAC1 (Yuan et al., 2008). These findings point to VDAC1 as endostatin target.

*Fluoxetine* (also known as Prozac, Sarafem, Fontex) is an anti-depressant, acting as a selective serotonin re-uptake inhibitor and is clinically used to treat different major depressions, obsessive-compulsive disorder, bulimia nervosa, and panic disorders, among other psychiatric conditions (Wong et al., 1995). The connection between fluoxetine and apoptosis is clouded by a series of contradictory findings. Fluoxetine was reported to enhance apoptosis in different cancer cells and tumors (Cloonan et al., 2010; Lee et al., 2010; Frick et al., 2011), yet was suggested to prevent apoptosis in both cancerous and neuronal cells (Wright et al., 1994; Lee et al., 2001; Chiou et al., 2006; Wang et al., 2011). Fluoxetine was shown to interact with VDAC1 and to increase the voltage-dependence of bilayer-reconstituted VDAC1 (Nahon et al., 2005; Thinnes, 2005). Additionally, fluoxetine was shown to prevent PTP opening and inhibited the release of Cyto *c* and apoptosis (Nahon et al., 2005). Although its remains unclear as to whether fluoxetine has an anti- or pro-apoptotic effect, it does seem that the compound mediates its effect on apoptosis via interactions with VDAC1.

*Oblimersen* (G3139) is an 18-mer phosphorothioate anti-sense oligonucleotide being studied as a possible treatment for several types of cancer, including CLL (Advani et al., 2011), B cell lymphoma (Pro et al., 2008), breast cancer (Rom et al., 2009), and melanoma (Lai et al., 2006). Although oblimersen is designed to target the initiation codon region of Bcl-2 mRNA, it was found to directly interact with VDAC1 and reduce its channel activity (Lai et al., 2006; Tan et al., 2007). Oblimersen is reported to induce caspase-dependent apoptosis via the intrinsic mitochondrial pathway in a Bcl-2-independent mechanism (Lai et al., 2006).

*Geldanamycin* (17AAG) is a derivative of the antibiotic, ansamycin. Geldanamycin binds to a conserved pocket in the amino-terminal portion of the Hsp90 chaperone protein and inhibits its function (Isaacs et al., 2003). *In vitro*, 17AAG acted as a Ca^2+^ mitochondrial regulator, increasing intracellular Ca^2+^ and diminishing the plasma membrane cationic current (Xie et al., 2011). 17AAG was shown to interact with VDAC through a hydrophobic interaction that was independent of Hsp90. This interaction was proposed to mediate inhibition of glioblastoma cell invasion (Xie et al., 2011).

## FINAL PERSPECTIVE

In recent years, we have witnessed a switch in the focus of VDAC research from channel activity properties and regulation at the level of purified protein in yeast and *Neurospora* to studying its function in mammalian cell cultures and animal models. Indeed, a significant accumulation of knowledge is now available concerning VDAC1 function in cell life and death. Much has been learned about VDAC function in regulating metabolite cross-talk between the mitochondria and rest of the cell, its function in mitochondria–ER Ca^2+^ signaling, its function in apoptosis and its involvement in various maladies from neurodegenerative diseases to cancer. The studies reviewed above have demonstrated the pivotal roles of mitochondria and VDAC1 in cancer. The dual functions of VDAC1 in both metabolism and apoptosis, as mediated by the protein alone and regulated by associated polypeptides, point to VDAC1 as begin a critical target in addressing cancer therapy.

Although a high-resolution structure was determined for recombinant VDAC1, many questions concerning the architecture of the channel pore, the location of modulator-binding sites, the protein regions involved in the control mechanism of VDAC1 oligomerization and how VDAC is regulated to serve its multiple roles remain unanswered. Likewise, additional studies are required to investigate whether the increased dependence of cancer cells on glycolytic metabolism can be exploited therapeutically.

VDAC1 is integral to mitochondrial ATP production as a metabolite transporter, as the docking site for mitochondrial-bound HK, and is highly expressed in cancers. VDAC1 is associated with the mitochondrial pathway of apoptosis, interacting with over-expressed anti-apoptotic proteins present in cancer and mediating the actions of some anti-cancer drugs. In addition, VDAC1 mediates cholesterol transport and distribution in the mitochondrial membrane, with cancer cells exhibiting several fold higher cholesterol levels than do healthy cells. VDAC1 also functions in ROS production and transport to the cytosol, with elevated ROS generation being seen in cancer cells. These functions, together with VDAC1 being over-expressed in some cancer cells, point to VDAC1 as being a rational target for the development of a new generation of therapeutics.

Delineating the molecular mechanisms underlying the re-programmed metabolism seen in cancers and their cell survival strategies, together with increasing understanding of the difficulty in targeting signal transduction in tumors without significant side-effects, encourages prospects for the development of effective cancer therapies. Thus, the emergence of targeted therapies specifically targeting critical molecules important for cancer cells may provide promising means of selectively killing these malignant cells. VDAC1 is one such a target. Indeed, various strategies involving interference RNA to down-regulate VDAC1 expression levels, VDAC1-based peptides and small molecules, such as methyl jasmonate, are being considered in this capacity. Finally, the discovery that VDAC plays a role at critical control points during energy metabolism and in apoptosis points to VDAC as a novel pharmacologic target for anti-cancer drugs.

## Conflict of Interest Statement

The authors declare that the research was conducted in the absence of any commercial or financial relationships that could be construed as a potential conflict of interest.

## Abbreviations

AIF: apoptosis-inducing factor
ANT: adenine nucleotide translocase
APAF-1: apoptosis protease-activating factor 1
Bcl-2: B cell lymphoma 2
caspase: cysteinyl/aspartate-specific protease
CLL: chronic lymphocytic leukemia
CyP D: cyclophilin D
Cyto *c*: cytochrome *c*
DIDS: 4,4′-diisothiocyanatostilbene-2,2′-disulfonic acid
ER: endoplasmic reticulum
FNQs: furanonaphthoquinones
G-6-P: glucose-6-phosphate
HK: hexokinase
IMM: inner mitochondrial membrane
IP_3_Rs: inositol-1,4,5-trisphosphate receptors
LDH: lactate dehydrogenase
MAC: mitochondrial apoptosis-inducing channel
NSCLC: non-small cell lung cancer
OMM: outer mitochondrial membrane
PDH: pyruvate dehydrogenase
PLB: planar lipid bilayer
PT: permeability transition
PTP: permeability transition pore
ROS: reactive oxygen species
RuR: ruthenium red
SR: sarcoplasmic reticulum
STS: staurosporine
TNF-α: tumor necrosis factor alpha
TSPO: translocator protein
UCP: uncoupling protein
VDAC: voltage-dependent anion channel.

## References

[B1] Abu-Hamad S. Arbel N. Calo D. Arzoine L. Israelson A. Keinan N. Ben-Romano R. (2009). The VDAC1 N-terminus is essential both for apoptosis and the protective effect of anti-apoptotic proteins. *J. Cell Sci.* 122(Pt 11) 1906–1916 19461077 10.1242/jcs.040188

[B2] Abu-Hamad S. Sivan S. Shoshan-Barmatz V. (2006). The expression level of the voltage-dependent anion channel controls life and death of the cell. *Proc. Natl. Acad. Sci. U.S.A.* 103 5787–5792 16585511 10.1073/pnas.0600103103 PMC1458651

[B3] Abu-Hamad S. Zaid H. Israelson A. Nahon E. Shoshan-Barmatz V. (2008). Hexokinase-I protection against apoptotic cell death is mediated via interaction with the voltage-dependent anion channel-1: mapping the site of binding. *J. Biol. Chem.* 283 13482–13490 18308720 10.1074/jbc.M708216200

[B4] Adachi M. Higuchi H. Miura S. Azuma T. Inokuchi S. Saito H. (2004). Bax interacts with the voltage-dependent anion channel and mediates ethanol-induced apoptosis in rat hepatocytes. *Am. J. Physiol. Gastrointest. Liver Physiol.* 287 G695–G705 15044178 10.1152/ajpgi.00415.2003

[B5] Adams J. D. Jr. Klaidman L. K. (1993). Acrolein-induced oxygen radical formation. *Free Radic. Biol. Med.* 15 187–193 8397144 10.1016/0891-5849(93)90058-3

[B6] Adams J. M. Cory S. (2007). The Bcl-2 apoptotic switch in cancer development and therapy. *Oncogene* 26 1324–1337 17322918 10.1038/sj.onc.1210220 PMC2930981

[B7] Adams V. Griffin L. Towbin J. Gelb B. Worley K. McCabe E. R. (1991). Porin interaction with hexokinase and glycerol kinase: metabolic microcompartmentation at the outer mitochondrial membrane. *Biochem. Med. Metab. Biol.* 45 271–291 1710914 10.1016/0885-4505(91)90032-g

[B8] Advani P. P. Paulus A. Masood A. Sher T. Chanan-Khan A. (2011). Pharmacokinetic evaluation of oblimersen sodium for the treatment of chronic lymphocytic leukemia. *Expert Opin. Drug Metab. Toxicol.* 7 765–774 21521129 10.1517/17425255.2011.579105

[B9] Ahmad A. Ahmad S. Schneider B. K. Allen C. B. Chang L. Y. White C. W. (2002). Elevated expression of hexokinase II protects human lung epithelial-like A549 cells against oxidative injury. *Am. J. Physiol. Lung Cell Mol. Physiol.* 283 L573–L584 12169577 10.1152/ajplung.00410.2001

[B10] Al-Abdulla N. A. Portera-Cailliau C. Martin L. J. (1998). Occipital cortex ablation in adult rat causes retrograde neuronal death in the lateral geniculate nucleus that resembles apoptosis. *Neuroscience* 86 191–209 9692754 10.1016/s0306-4522(98)00014-1

[B11] Andre N. Braguer D. Brasseur G. Gonçalves A. Lemesle-Meunier D. Guise S. (2000). Paclitaxel induces release of cytochrome c from mitochondria isolated from human neuroblastoma cells’. *Cancer Res.* 60 5349–5353 11034069

[B12] Anflous-Pharayra K. Lee N. Armstrong D. L. Craigen W. J. (2010). VDAC3 has differing mitochondrial functions in two types of striated muscles. *Biochim. Biophys. Acta* 1807 150–156 20875390 10.1016/j.bbabio.2010.09.007 PMC2998388

[B13] Antignani A. Youle R. J. (2006). How do Bax and Bak lead to permeabilization of the outer mitochondrial membrane? *Curr. Opin. Cell Biol.* 18 685–689 17046225 10.1016/j.ceb.2006.10.004

[B14] Antonsson B. (2001). Bax and other pro-apoptotic Bcl-2 family “killer-proteins” and their victim the mitochondrion. *Cell Tissue Res.* 306 347–361 11735035 10.1007/s00441-001-0472-0

[B15] Antonsson B. Montessuit S. Lauper S. Eskes R. Martinou J. C. (2000). Bax oligomerization is required for channel-forming activity in liposomes and to trigger cytochrome *c* release from mitochondria. *Biochem. J.* 345(Pt 2) 271–278 10620504 PMC1220756

[B16] Antonsson B. Montessuit S. Sanchez B. Martinou J. C. (2001). Bax is present as a high molecular weight oligomer/complex in the mitochondrial membrane of apoptotic cells. *J. Biol. Chem.* 276 11615–11623 11136736 10.1074/jbc.M010810200

[B17] Arbel N. Ben-Hail D. Shoshan-Barmatz V. (2012). Mediation of the antiapoptotic activity of Bcl-xL protein upon interaction with VDAC1 protein. *J. Biol. Chem.* 287 23152–23161 22589539 10.1074/jbc.M112.345918 PMC3391160

[B18] Arbel N. Shoshan-Barmatz V. (2010). Voltage-dependent anion channel 1-based peptides interact with Bcl-2 to prevent antiapoptotic activity. *J. Biol. Chem.* 285 6053–6062 20037155 10.1074/jbc.M109.082990 PMC2825399

[B19] Arzoine L. Zilberberg N. Ben-Romano R. Shoshan-Barmatz V. (2009). Voltage-dependent anion channel 1-based peptides interact with hexokinase to prevent its anti-apoptotic activity. *J. Biol. Chem.* 284 3946–3955 19049977 10.1074/jbc.M803614200

[B20] Auten R. L. Davis J. M. (2009). Oxygen toxicity and reactive oxygen species: the devil is in the details. *Pediatr. Res.* 66 121–127 19390491 10.1203/PDR.0b013e3181a9eafb

[B21] Azoulay-Zohar H. Aflalo C. (1999). Binding of rat brain hexokinase to recombinant yeast mitochondria: identification of necessary molecular determinants. *J. Bioenerg. Biomembr.* 31 569–579 10682915 10.1023/a:1005469028274

[B22] Azoulay-Zohar H. Israelson A. Abu-Hamad S. Shoshan-Barmatz V. (2004). In self-defence: hexokinase promotes voltage-dependent anion channel closure and prevents mitochondria-mediated apoptotic cell death. *Biochem. J.* 377(Pt 2) 347–355 14561215 10.1042/BJ20031465 PMC1223882

[B23] Bae J. H. Park J. W. Kwon T. K. (2003). Ruthenium red, inhibitor of mitochondrial Ca^2+^ uniporter, inhibits curcumin-induced apoptosis via the prevention of intracellular Ca^2+^ depletion and cytochrome c release. *Biochem. Biophys. Res. Commun.* 303 1073–1079 12684045 10.1016/s0006-291x(03)00479-0

[B24] Baek J. H. Lee Y. S. Kang C. M. Kim J. A. Kwon K. S. Son H. C. (1997). Intracellular Ca^2+^ release mediates ursolic acid-induced apoptosis in human leukemic HL-60 cells. *Int. J. Cancer* 73 725–728 9398053 10.1002/(sici)1097-0215(19971127)73:5<725::aid-ijc19>3.0.co;2-4

[B25] Baggetto L. G. Clottes E. Vial C. (1992). Low mitochondrial proton leak due to high membrane cholesterol content and cytosolic creatine kinase as two features of the deviant bioenergetics of Ehrlich and AS30-D tumor cells. *Cancer Res.* 52 4935–4941 1516050

[B26] Baines C. P. Kaiser R. A. Sheiko T. Craigen W. J. Molkentin J. D. (2007). Voltage-dependent anion channels are dispensable for mitochondrial-dependent cell death. *Nat. Cell Biol.* 9 550–555 17417626 10.1038/ncb1575 PMC2680246

[B27] Banerjee J. Ghosh S. (2004). Bax increases the pore size of rat brain mitochondrial voltage-dependent anion channel in the presence of tBid. *Biochem. Biophys. Res. Commun.* 323 310–314 15351738 10.1016/j.bbrc.2004.08.094

[B28] Barber S. C. Mead R. J. Shaw P. J. (2006). Oxidative stress in ALS: a mechanism of neurodegeneration and a therapeutic target. *Biochim. Biophys. Acta* 1762 1051–1067 16713195 10.1016/j.bbadis.2006.03.008

[B29] Batarseh A. Papadopoulos V. (2010). Regulation of translocator protein 18 kDa (TSPO) expression in health and disease states. *Mol. Cell. Endocrinol.* 327 1–12 20600583 10.1016/j.mce.2010.06.013 PMC2922062

[B30] Bathori G. Csordás G. Garcia-Perez C. Davies E Hajnóczky G. (2006). Ca^2+^-dependent control of the permeability properties of the mitochondrial outer membrane and voltage-dependent anion-selective channel (VDAC). *J. Biol. Chem.* 281 17347–17358 16597621 10.1074/jbc.M600906200

[B31] Bathori G. Parolini I. Szabó I. Tombola F. Messina A. Oliva M. (2000). Extramitochondrial porin: facts and hypotheses. *J. Bioenerg. Biomembr.* 32 79–89 11768765 10.1023/a:1005516513313

[B32] Bathori G. Parolini I. Tombola F. Szabò I. Messina A. Oliva M. (1999). Porin is present in the plasma membrane where it is concentrated in caveolae and caveolae-related domains. *J. Biol. Chem.* 274 29607–29612 10514428 10.1074/jbc.274.42.29607

[B33] Baughman J. M. Perocchi F. Girgis H. S. Plovanich M. Belcher-Timme C. A. Sancak Y. (2011). Integrative genomics identifies MCU as an essential component of the mitochondrial calcium uniporter. *Nature* 476 341–345 21685886 10.1038/nature10234 PMC3486726

[B34] Baumgartner H. K. Gerasimenko J. V. Thorne C. Ferdek P. Pozzan T. Tepikin A. V. (2009). Calcium elevation in mitochondria is the main Ca^2+^ requirement for mitochondrial permeability transition pore (mPTP) opening. *J. Biol. Chem.* 284 20796–20803 19515844 10.1074/jbc.M109.025353 PMC2742844

[B35] Bayrhuber M. Meins T. Habeck M. Becker S. Giller K. Villinger S. (2008). Structure of the human voltage-dependent anion channel. *Proc. Natl. Acad. Sci. U.S.A.* 105 15370–15375 18832158 10.1073/pnas.0808115105 PMC2557026

[B36] Belizario J. E. Alves J. Occhiucci J. M. Garay-Malpartida M. Sesso A. (2007). A mechanistic view of mitochondrial death decision pores. *Braz. J. Med. Biol. Res.* 40 1011–1024 17665037 10.1590/s0100-879x2006005000109

[B37] Belzacq A. S. Jacotot E. Vieira H. L. Mistro D. Granville D. J. Xie Z. Reed J. C. (2001). Apoptosis induction by the photosensitizer verteporfin: identification of mitochondrial adenine nucleotide translocator as a critical target. *Cancer Res.* 61 1260–1264 11245415

[B38] Benz R. (1994). Permeation of hydrophilic solutes through mitochondrial outer membranes: review on mitochondrial porins. *Biochim. Biophys. Acta* 1197 167–196 8031826 10.1016/0304-4157(94)90004-3

[B39] Bernardi P. (1999). Mitochondrial transport of cations: channels, exchangers, and permeability transition. *Physiol. Rev.* 79 1127–1155 10508231 10.1152/physrev.1999.79.4.1127

[B40] Berridge M. J. Lipp P. Bootman M. D. (2000). The versatility and universality of calcium signalling. *Nat. Rev. Mol. Cell Biol.* 1 11–21 11413485 10.1038/35036035

[B41] Berridge M. V. Herst P. M. Lawen A. (2009). Targeting mitochondrial permeability in cancer drug development. *Mol. Nutr. Food Res.* 53 76–86 19035550 10.1002/mnfr.200700493

[B42] Berridge M. V. Herst P. M. Tan A. S. (2010). Metabolic flexibility and cell hierarchy in metastatic cancer. *Mitochondrion* 10 584–588 20709626 10.1016/j.mito.2010.08.002

[B43] Berry M. D. Boulton A. A. (2000). Glyceraldehyde-3-phosphate dehydrogenase and apoptosis. *J. Neurosci. Res.* 60 150–154 10740219 10.1002/(SICI)1097-4547(20000415)60:2<150::AID-JNR3>3.0.CO;2-4

[B44] Betaneli V. Petrov E. P. Schwille P. (2012). The role of lipids in VDAC oligomerization. *Biophys. J.* 102 523–531 22325275 10.1016/j.bpj.2011.12.049 PMC3274789

[B45] Beutner G. Rück A. Riede B. Brdiczka D. (1998). Complexes between porin, hexokinase, mitochondrial creatine kinase and adenylate translocator display properties of the permeability transition pore. Implication for regulation of permeability transition by the kinases. *Biochim. Biophys. Acta* 1368 7–18 9459579 10.1016/s0005-2736(97)00175-2

[B46] Beutner G. Ruck A. Riede B. Welte W. Brdiczka D. (1996). Complexes between kinases, mitochondrial porin and adenylate translocator in rat brain resemble the permeability transition pore. *FEBS Lett.* 396 189–195 8914985 10.1016/0014-5793(96)01092-7

[B47] Birbes H. Luberto C. Hsu Y. T. El Bawab S. Hannun Y. A. Obeid L. M. (2005). A mitochondrial pool of sphingomyelin is involved in TNFalpha-induced Bax translocation to mitochondria. *Biochem. J.* 386(Pt 3) 445–451 15516208 10.1042/BJ20041627 PMC1134862

[B48] Blachly-Dyson E. Zambronicz E. B. Yu W. H. Adams V. McCabe E. R. Adelman J. (1993). Cloning and functional expression in yeast of two human isoforms of the outer mitochondrial membrane channel, the voltage-dependent anion channel. *J. Biol. Chem.* 268 1835–1841 8420959

[B49] Bononi A. Missiroli S. Poletti F. Suski J. M. Agnoletto C. Bonora M. (2012). Mitochondria-associated membranes (MAMs) as hotspot Ca(2+) signaling units. *Adv. Exp. Med. Biol.* 740 411–437 22453952 10.1007/978-94-007-2888-2_17

[B50] Bootman M. D. Petersen O. H. Verkhratsky A. (2002). The endoplasmic reticulum is a focal point for co-ordination of cellular activity. *Cell Calcium* 32 231–234 12543085 10.1016/s0143416002002002

[B51] Boya P. Pauleau A. L. Poncet D. Gonzalez-Polo R. A. Zamzami N. Kroemer G. (2004). Viral proteins targeting mitochondria: controlling cell death. *Biochim. Biophys. Acta* 1659 178–189 15576050 10.1016/j.bbabio.2004.08.007

[B52] Boya P. Roques B. Kroemer G. (2001). New EMBO members’ review: viral and bacterial proteins regulating apoptosis at the mitochondrial level. *EMBO J.* 20 4325–4331 11500358 10.1093/emboj/20.16.4325 PMC125565

[B53] Boya P. Roumier T. Andreau K. Gonzalez-Polo R. A. Zamzami N. Castedo M. (2003). Mitochondrion-targeted apoptosis regulators of viral origin. *Biochem. Biophys. Res. Commun.* 304 575–581 12729592 10.1016/s0006-291x(03)00630-2

[B54] Brahimi-Horn M. C. Ben-Hail D. Ilie M. Gounon P. Rouleau M. Hofman V. (2012). Expression of a truncated active form of VDAC1 in lung cancer associates with hypoxic cell survival and correlates with progression to chemotherapy resistance. *Cancer Res.* 72 2140–2150 22389449 10.1158/0008-5472.CAN-11-3940

[B55] Brahimi-Horn M. C. Chiche J. Pouyssegur J. (2007). Hypoxia signalling controls metabolic demand. *Curr. Opin. Cell Biol.* 19 223–229 17303407 10.1016/j.ceb.2007.02.003

[B56] Brdiczka D. Kaldis P. Wallimann T. (1994). In vitro complex formation between the octamer of mitochondrial creatine kinase and porin. *J. Biol. Chem.* 269 27640–27644 7525559

[B57] Brdiczka D. G. Zorov D. B. Sheu S. S. (2006). Mitochondrial contact sites: their role in energy metabolism and apoptosis. *Biochim. Biophys. Acta* 1762 148–163 16324828 10.1016/j.bbadis.2005.09.007

[B58] Brookes P. S. Salinas E. P. Darley-Usmar K. Eiserich J. P. Freeman B. A. Darley-Usmar V. M. (2000). Concentration-dependent effects of nitric oxide on mitochondrial permeability transition and cytochrome c release. *J. Biol. Chem.* 275 20474–20479 10791954 10.1074/jbc.M001077200

[B59] Brown R. (1997). The bcl-2 family of proteins. *Br. Med. Bull.* 53 466–477 9374031 10.1093/oxfordjournals.bmb.a011624

[B60] Brune B. von Knethen A. Sandau K. B. (1999). Nitric oxide (NO): an effector of apoptosis. *Cell Death Differ.* 6 969–975 10556974 10.1038/sj.cdd.4400582

[B61] Brustovetsky N. Dubinsky J. M. Antonsson B. Jemmerson R. (2003). Two pathways for tBID-induced cytochrome *c* release from rat brain mitochondria: BAK- versus BAX-dependence. *J. Neurochem.* 84 196–207 12485416 10.1046/j.1471-4159.2003.01545.x

[B62] Bryson J. M. Coy P. E. Gottlob K. Hay N. Robey R. B. (2002). Increased hexokinase activity, of either ectopic or endogenous origin, protects renal epithelial cells against acute oxidant-induced cell death. *J. Biol. Chem.* 277 11392–11400 11751868 10.1074/jbc.M110927200

[B63] Buettner R. Papoutsoglou G. Scemes E. Spray D. C. Dermietzel R. (2000). Evidence for secretory pathway localization of a voltage-dependent anion channel isoform. *Proc. Natl. Acad. Sci. U.S.A.* 97 3201–3206 10716730 10.1073/pnas.060242297 PMC16216

[B64] Bühler S. Michels J. Wendt S. Rück A. Brdiczka D. Welte W. (1998). Mass spectrometric mapping of ion channel proteins (porins) and identification of their supramolecular membrane assembly. *Proteins* 33(Suppl. 2) 63–73 10.1002/(sici)1097-0134(1998)33:2+<63::aid-prot8>3.3.co;2-9 9849911

[B65] Bustamante E. Pedersen P. L. (1980). Mitochondrial hexokinase of rat hepatoma cells in culture: solubilization and kinetic properties. *Biochemistry* 19 4972–4977 6779859 10.1021/bi00563a006

[B66] Cairns R. A. Harris I. S. Mak T. W. (2011). Regulation of cancer cell metabolism. *Nat. Rev. Cancer* 11 85–95 21258394 10.1038/nrc2981

[B67] Campbell A. M. Chan S. H. (2008). Mitochondrial membrane cholesterol, the voltage dependent anion channel (VDAC), and the Warburg effect. *J. Bioenerg. Biomembr.* 40 193–197 18677555 10.1007/s10863-008-9138-x

[B68] Capano M. Crompton M. (2002). Biphasic translocation of Bax to mitochondria. *Biochem. J.* 367(Pt 1) 169–178 12097139 10.1042/BJ20020805 PMC1222873

[B69] Cárdenas C. Miller R. A. Smith I. Bui T. Molgó J. Müller M. (2010). Essential regulation of cell bioenergetics by constitutive InsP3 receptor Ca^2+^ transfer to mitochondria. *Cell* 142 270–283 20655468 10.1016/j.cell.2010.06.007 PMC2911450

[B70] Carre M. André N. Carles G. Borghi H. Brichese L. Briand C. (2002). Tubulin is an inherent component of mitochondrial membranes that interacts with the voltage-dependent anion channel. *J. Biol. Chem.* 277 33664–33669 12087096 10.1074/jbc.M203834200

[B71] Casadio R. Jacoboni I. Messina A De Pinto V. (2002). A 3D model of the voltage-dependent anion channel (VDAC). *FEBS Lett.* 520 1–7 12044860 10.1016/s0014-5793(02)02758-8

[B72] Cassara M. C. Menzel V. A. Hinsch K. D. Wrenzycki C. Hinsch E. (2010). Voltage-dependent anion channels 1 and 2 are expressed in porcine oocytes. *Biosci. Rep.* 30 193–200 19630752 10.1042/BSR20090088

[B73] Castagna A. Antonioli P. Astner H. Hamdan M. Righetti S. C. Perego P. (2004). A proteomic approach to cisplatin resistance in the cervix squamous cell carcinoma cell line A431. *Proteomics* 4 3246–3267 15378690 10.1002/pmic.200400835

[B74] Castedo M. Perfettini J. L. Andreau K. Roumier T. Piacentini M. Kroemer G. (2003). Mitochondrial apoptosis induced by the HIV-1 envelope. *Ann. N. Y. Acad. Sci.* 1010 19–28 15033690 10.1196/annals.1299.004

[B75] Certo M. Del Gaizo Moore V. Nishino M. Wei G. Korsmeyer S. Armstrong S. A. (2006). Mitochondria primed by death signals determine cellular addiction to antiapoptotic BCL-2 family members. *Cancer Cell* 9 351–365 16697956 10.1016/j.ccr.2006.03.027

[B76] Cesar Mde C. Wilson J. E. (2004). All three isoforms of the voltage-dependent anion channel (VDAC1, VDAC2, and VDAC3) are present in mitochondria from bovine, rabbit, and rat brain. *Arch. Biochem. Biophys.* 422 191–196 14759607 10.1016/j.abb.2003.12.030

[B77] Chacko A. D. Liberante F. Paul I. Longley D. B. Fennell D. A. (2010). Voltage dependent anion channel-1 regulates death receptor mediated apoptosis by enabling cleavage of caspase-8. *BMC Cancer* 10 380 10.1186/1471-2407-10-380 PMC2913963 20646307

[B78] Cheng E. H. Sheiko T. V. Fisher J. K. Craigen W. J. Korsmeyer S. J. (2003). VDAC2 inhibits BAK activation and mitochondrial apoptosis. *Science* 301 513–517 12881569 10.1126/science.1083995

[B79] Cheng Q. Sedlic F. Pravdic D. Bosnjak Z. J. Kwok W. M. (2011). Biphasic effect of nitric oxide on the cardiac voltage-dependent anion channel. *FEBS Lett.* 585 328–334 21156174 10.1016/j.febslet.2010.12.008 PMC3035949

[B80] Cheng S. L. Liu R. H. Sheu J. N. Chen S. T. Sinchaikul S. Tsay G. J. (2007). Toxicogenomics of A375 human malignant melanoma cells treated with arbutin. *J. Biomed. Sci.* 14 87–105 17103032 10.1007/s11373-006-9130-6

[B81] Chiou S. H. Chen S. J. Peng C. H. Chang Y. L. Ku H. H. Hsu W. M. (2006). Fluoxetine up-regulates expression of cellular FLICE-inhibitory protein and inhibits LPS-induced apoptosis in hippocampus-derived neural stem cell. *Biochem. Biophys. Res. Commun.* 343 391–400 16545775 10.1016/j.bbrc.2006.02.180

[B82] Chittenden T. Harrington E. A. O’Connor R. Flemington C. Lutz R. J. Evan G. I. (1995). Induction of apoptosis by the Bcl-2 homologue Bak. *Nature* 374 733–736 7715730 10.1038/374733a0

[B83] Choudhary C. Kumar C. Gnad F. Nielsen M. L. Rehman M. Walther T. C. (2009). Lysine acetylation targets protein complexes and co-regulates major cellular functions. *Science* 325 834–840 19608861 10.1126/science.1175371

[B84] Choudhary O. P. Ujwal R. Kowallis W. Coalson R. Abramson J. Grabe M. (2010). The electrostatics of VDAC: implications for selectivity and gating. *J. Mol. Biol.* 396 580–592 20005234 10.1016/j.jmb.2009.12.006 PMC3736979

[B85] Chowdhury I. Tharakan B. Bhat G. K. (2006). Current concepts in apoptosis: the physiological suicide program revisited. *Cell. Mol. Biol. Lett.* 11 506–525 16977376 10.2478/s11658-006-0041-3 PMC6275981

[B86] Cloonan S. M. Drozgowska A. Fayne D. Williams D. C. (2010). The antidepressants maprotiline and fluoxetine have potent selective antiproliferative effects against Burkitt lymphoma independently of the norepinephrine and serotonin transporters. *Leuk. Lymphoma* 51 523–539 20141432 10.3109/10428190903552112

[B87] Colombini M. (1980). Structure and mode of action of a voltage dependent anion-selective channel (VDAC) located in the outer mitochondrial membrane. *Ann. N. Y. Acad. Sci.* 341 552–563 6249159 10.1111/j.1749-6632.1980.tb47198.x

[B88] Colombini M. (1989). Voltage gating in the mitochondrial channel, VDAC. *J. Membr. Biol.* 111 103–111 2482359 10.1007/BF01871775

[B89] Colombini M. (2004). VDAC: the channel at the interface between mitochondria and the cytosol. *Mol. Cell. Biochem.* 256–257 107–115 10.1023/b:mcbi.0000009862.17396.8d 14977174

[B90] Colombini M. (2009). The published 3D structure of the VDAC channel: native or not? *Trends Biochem. Sci.* 34 382–389 19647437 10.1016/j.tibs.2009.05.001

[B91] Cory S. Adams J. M. (2002). The Bcl2 family: regulators of the cellular life-or-death switch. *Nat. Rev. Cancer* 2 647–656 12209154 10.1038/nrc883

[B92] Cory S. Huang D. C. Adams J. M. (2003). The Bcl-2 family: roles in cell survival and oncogenesis. *Oncogene* 22 8590–8607 14634621 10.1038/sj.onc.1207102

[B93] Costantini P. Jacotot E. Decaudin D. Kroemer G. (2000). Mitochondrion as a novel target of anticancer chemotherapy. *J. Natl. Cancer Inst.* 92 1042–1053 10880547 10.1093/jnci/92.13.1042

[B94] Cox D. A. Matlib M. A. (1993). A role for the mitochondrial Na(+)-Ca^2+^ exchanger in the regulation of oxidative phosphorylation in isolated heart mitochondria. *J. Biol. Chem.* 268 938–947 8419373

[B95] Crompton M. (1999). The mitochondrial permeability transition pore and its role in cell death. *Biochem. J.* 341(Pt 2) 233–249 10393078 PMC1220352

[B96] Csordas G. Madesh M. Antonsson B Hajnóczky G. (2002). tcBid promotes Ca(2+) signal propagation to the mitochondria: control of Ca(2+) permeation through the outer mitochondrial membrane. *EMBO J.* 21 2198–2206 11980717 10.1093/emboj/21.9.2198 PMC125984

[B97] Cullen K. J. Yang Z. Schumaker L. Guo Z. (2007). Mitochondria as a critical target of the chemotheraputic agent cisplatin in head and neck cancer. *J. Bioenerg. Biomembr.* 39 43–50 17318397 10.1007/s10863-006-9059-5

[B98] Dang T. N. Arseneault M. Murthy V. Ramassamy C. (2010). Potential role of acrolein in neurodegeneration and in Alzheimer’s disease. *Curr. Mol. Pharmacol.* 3 66–78 20302565

[B99] Danishuddin M. Khan S. N. Khan A. U. (2009). Molecular interactions between mitochondrial membrane proteins and the C-terminal domain of PB1-F2: an in silico approach. *J. Mol. Model.* 16 535–541 19669810 10.1007/s00894-009-0555-5

[B100] da-Silva W. S. Gómez-Puyou A. de Gómez-Puyou M. T. Moreno-Sanchez R. De Felice F. G. de Meis L. (2004). Mitochondrial bound hexokinase activity as a preventive antioxidant defense: steady-state ADP formation as a regulatory mechanism of membrane potential and reactive oxygen species generation in mitochondria. *J. Biol. Chem.* 279 39846–39855 15247300 10.1074/jbc.M403835200

[B101] Debatin K. M. Poncet D. Kroemer G. (2002). Chemotherapy: targeting the mitochondrial cell death pathway. *Oncogene* 21 8786–8803 12483532 10.1038/sj.onc.1206039

[B102] Decuypere J. P. Monaco G. Missiaen L. De Smedt H. Parys J. B. Bultynck G. (2011). IP(3) receptors, mitochondria, and Ca signaling: implications for aging. *J. Aging Res.* 2011 920178 10.4061/2011/920178 PMC3056293 21423550

[B103] Dejean L. M. Martinez-Caballero S. Guo L. Hughes C. Teijido O. Ducret T. (2005). Oligomeric Bax is a component of the putative cytochrome c release channel MAC, mitochondrial apoptosis-induced channel. *Mol. Biol. Cell* 16 2424–2432 15772159 10.1091/mbc.E04-12-1111 PMC1087246

[B104] Dejean L. M. Martinez-Caballero S. Kinnally K. W. (2006a). Is MAC the knife that cuts cytochrome *c* from mitochondria during apoptosis? *Cell Death Differ.* 13 1387–1395 16676005 10.1038/sj.cdd.4401949

[B105] Dejean L. M. Martinez-Caballero S. Manon S. Kinnally K. W. (2006b). Regulation of the mitochondrial apoptosis-induced channel, MAC, by BCL-2 family proteins. *Biochim. Biophys. Acta* 1762 191–201 16055309 10.1016/j.bbadis.2005.07.002

[B106] Deniaud A. Sharaf el dein O. Maillier E. Poncet D. Kroemer G. Lemaire C. (2008). Endoplasmic reticulum stress induces calcium-dependent permeability transition, mitochondrial outer membrane permeabilization and apoptosis. *Oncogene* 27 285–299 17700538 10.1038/sj.onc.1210638

[B107] Denning M. F. Wang Y. Tibudan S. Alkan S. Nickoloff B. J. Qin J. Z. (2002). Caspase activation and disruption of mitochondrial membrane potential during UV radiation-induced apoptosis of human keratinocytes requires activation of protein kinase C. *Cell Death Differ*. 9 40–52 11803373 10.1038/sj.cdd.4400929

[B108] Denton R. M. (2009). Regulation of mitochondrial dehydrogenases by calcium ions. *Biochim. Biophys. Acta* 1787 1309–1316 19413950 10.1016/j.bbabio.2009.01.005

[B109] De Pinto V. Benz R. Palmieri F. (1989). Interaction of non-classical detergents with the mitochondrial porin. A new purification procedure and characterization of the pore-forming unit. *Eur. J. Biochem.* 183 179–187 2546771 10.1111/j.1432-1033.1989.tb14911.x

[B110] De Pinto V. Guarino F. Guarnera A. Messina A. Reina S. Tomasello F. M. (2010a). Characterization of human VDAC isoforms: a peculiar function for VDAC3? *Biochim. Biophys. Acta* 1797 1268–1275 20138821 10.1016/j.bbabio.2010.01.031

[B111] De Pinto V. Messina A. Lane D. J. Lawen A. (2010b). Voltage-dependent anion-selective channel (VDAC) in the plasma membrane. *FEBS Lett.* 584 1793–1799 20184885 10.1016/j.febslet.2010.02.049

[B112] De Pinto V. Messina A. Accardi R. Aiello R. Guarino F. Tomasello M. F. (2003). New functions of an old protein: the eukaryotic porin or voltage dependent anion selective channel (VDAC). *Ital. J. Biochem.* 52 17–24 12833633

[B113] De Pinto V. Prezioso G. Thinnes F. Link T. A. Palmieri F. (1991). Peptide-specific antibodies and proteases as probes of the transmembrane topology of the bovine heart mitochondrial porin. *Biochemistry* 30 10191–10200 1718414 10.1021/bi00106a017

[B114] De Pinto V. Reina S. Guarino F. Messina A. (2008). Structure of the voltage dependent anion channel: state of the art. *J. Bioenerg. Biomembr.* 40 139–147 18668358 10.1007/s10863-008-9140-3

[B115] De Pinto V. Tomasello F. Messina A. Guarino F. Benz R. La Mendola D. (2007). Determination of the conformation of the human VDAC1 N-terminal peptide, a protein moiety essential for the functional properties of the pore. *Chembiochem* 8 744–756 17387661 10.1002/cbic.200700009

[B116] Desagher S. Osen-Sand A. Nichols A. Eskes R. Montessuit S. Lauper S. (1999). Bid-induced conformational change of Bax is responsible for mitochondrial cytochrome c release during apoptosis. *J. Cell Biol.* 144 891–901 10085289 10.1083/jcb.144.5.891 PMC2148190

[B117] De Stefani D. Bononi A. Romagnoli A. Messina A. De Pinto V. Pinton P. (2012). VDAC1 selectively transfers apoptotic Ca^2+^ signals to mitochondria. *Cell Death Differ.* 19 267–273 21720385 10.1038/cdd.2011.92 PMC3263501

[B118] De Stefani D. Raffaello A. Teardo E. Szabò I. Rizzuto R. (2011). A forty-kilodalton protein of the inner membrane is the mitochondrial calcium uniporter. *Nature* 476 336–340 21685888 10.1038/nature10230 PMC4141877

[B119] Ding W. Hudson L. G. Liu K. J. (2005). Inorganic arsenic compounds cause oxidative damage to DNA and protein by inducing ROS and RNS generation in human keratinocytes. *Mol. Cell. Biochem.* 279 105–112 16283519 10.1007/s11010-005-8227-y

[B120] Ding W. X. Shen H. M. Ong C. N. (2001). Pivotal role of mitochondrial Ca(2+) in microcystin-induced mitochondrial permeability transition in rat hepatocytes. *Biochem. Biophys. Res. Commun.* 285 1155–1161 11478775 10.1006/bbrc.2001.5309

[B121] Distler A. M. Kerner J. Hoppel C. L. (2007). Post-translational modifications of rat liver mitochondrial outer membrane proteins identified by mass spectrometry. *Biochim. Biophys. Acta* 1774 628–636 17478130 10.1016/j.bbapap.2007.03.012 PMC1950290

[B122] Dlugosz P. J. Billen L. P. Annis M. G. Zhu W. Zhang Z. Lin J. (2006). Bcl-2 changes conformation to inhibit Bax oligomerization. *EMBO J.* 25 2287–2296 16642033 10.1038/sj.emboj.7601126 PMC1478188

[B123] Dolder M. Zeth K. Tittmann P. Gross H. Welte W. Wallimann T. (1999). Crystallization of the human, mitochondrial voltage-dependent anion-selective channel in the presence of phospholipids. *J. Struct. Biol.* 127 64–71 10479618 10.1006/jsbi.1999.4141

[B124] Eskes R. Desagher S. Antonsson B. Martinou J. C. (2000). Bid induces the oligomerization and insertion of Bax into the outer mitochondrial membrane. *Mol. Cell. Biol.* 20 929–935 10629050 10.1128/mcb.20.3.929-935.2000 PMC85210

[B125] Everett H. McFadden G. (2001). Viruses and apoptosis: meddling with mitochondria. *Virology* 288 1–7 11543652 10.1006/viro.2001.1081

[B126] Fadeel B. Ottosson A. Pervaiz S. (2008). Big wheel keeps on turning: apoptosome regulation and its role in chemoresistance. *Cell Death Differ.* 15 443–452 17975549 10.1038/sj.cdd.4402265

[B127] Fanciulli M. Paggi M. G. Bruno T. Del Carlo C. Bonetto F. Gentile F. P. (1994). Glycolysis and growth rate in normal and in hexokinase-transfected NIH-3T3 cells. *Oncol. Res.* 6 405–409 7703526

[B128] Feldmann G. Haouzi D. Moreau A. Durand-Schneider A. M. Bringuier A. Berson A. (2000). Opening of the mitochondrial permeability transition pore causes matrix expansion and outer membrane rupture in Fas-mediated hepatic apoptosis in mice. *Hepatology* 31 674–683 10706558 10.1002/hep.510310318

[B129] Feng Z. Hu W. Hu Y. Tang M. S. (2006). Acrolein is a major cigarette-related lung cancer agent: preferential binding at p53 mutational hotspots and inhibition of DNA repair. *Proc. Natl. Acad. Sci. U.S.A.* 103 15404–15409 17030796 10.1073/pnas.0607031103 PMC1592536

[B130] Folkman J. (2006). Antiangiogenesis in cancer therapy – endostatin and its mechanisms of action. *Exp. Cell Res.* 312 594–607 16376330 10.1016/j.yexcr.2005.11.015

[B131] Franklin-Tong V. E. Gourlay C. W. (2008). A role for actin in regulating apoptosis/programmed cell death: evidence spanning yeast, plants and animals. *Biochem. J.* 413 389–404 18613816 10.1042/BJ20080320

[B132] Frick L. R. Rapanelli M. Arcos M. L. Cremaschi G. A. Genaro A. M. (2011). Oral administration of fluoxetine alters the proliferation/apoptosis balance of lymphoma cells and up-regulates T cell immunity in tumor-bearing mice. *Eur. J. Pharmacol.* 659 265–272 21497159 10.1016/j.ejphar.2011.03.037

[B133] Fulda S. Galluzzi L. Kroemer G. (2010). Targeting mitochondria for cancer therapy. *Nat. Rev. Drug Discov.* 9 447–464 20467424 10.1038/nrd3137

[B134] Fulda S. Scaffidi C. Susin S. A. Krammer P. H. Kroemer G. Peter M. E. (1998). Activation of mitochondria and release of mitochondrial apoptogenic factors by betulinic acid. *J. Biol. Chem.* 273 33942–33948 9852046 10.1074/jbc.273.51.33942

[B135] Gaikwad A. Poblenz A. Haridas V. Zhang C. Duvic M. Gutterman J. (2005). Triterpenoid electrophiles (avicins) suppress heat shock protein-70 and x-linked inhibitor of apoptosis proteins in malignant cells by activation of ubiquitin machinery: implications for proapoptotic activity. *Clin. Cancer Res.* 11 1953–1962 15756021 10.1158/1078-0432.CCR-04-1704

[B136] Galluzzi L. Kroemer G. (2007). Mitochondrial apoptosis without VDAC. *Nat. Cell Biol.* 9 487–489 17473857 10.1038/ncb0507-487

[B137] Gatenby R. A. Gillies R. J. (2004). Why do cancers have high aerobic glycolysis? *Nat. Rev. Cancer* 4 891–899 15516961 10.1038/nrc1478

[B138] Gauci S. Helbig A. O. Slijper M. Krijgsveld J. Heck A. J. Mohammed S. (2009). Lys-N and trypsin cover complementary parts of the phosphoproteome in a refined SCX-based approach. *Anal. Chem.* 81 4493–4501 19413330 10.1021/ac9004309

[B139] Gavish M. Bachman I. Shoukrun R. Katz Y. Veenman L. Weisinger G. (1999). Enigma of the peripheral benzodiazepine receptor. *Pharmacol. Rev.* 51 629–650 10581326

[B140] Gelb B. D. Adams V. Jones S. N. Griffin L. D. MacGregor G. R. McCabe E. R. (1992). Targeting of hexokinase 1 to liver and hepatoma mitochondria. *Proc. Natl. Acad. Sci. U.S.A.* 89 202–206 1309605 10.1073/pnas.89.1.202 PMC48204

[B141] Genini D. Adachi S. Chao Q. Rose D. W. Carrera C. J. Cottam H. B. (2000). Deoxyadenosine analogs induce programmed cell death in chronic lymphocytic leukemia cells by damaging the DNA and by directly affecting the mitochondria. *Blood* 96 3537–3543 11071652

[B142] Gerber D. Shai Y. (2001). In vivo detection of hetero-association of glycophorin-A and its mutants within the membrane. *J. Biol. Chem.* 276 31229–31232 11402026 10.1074/jbc.M101889200

[B143] Geula S. Ben-Hail D. Shoshan-Barmatz V. (2012a). Structure-based analysis of VDAC1: N-terminus location, translocation, channel gating and association with anti-apoptotic proteins. *Biochem. J.* 444 475–485 22397371 10.1042/BJ20112079

[B144] Geula S. Naveed H. Liang J. Shoshan-Barmatz V. (2012b). Structure-based analysis of VDAC1 protein: defining oligomer contact sites. *J. Biol. Chem.* 287 2179–2190 22117062 10.1074/jbc.M111.268920 PMC3265896

[B145] Ghosh T. Pandey N. Maitra A. Brahmachari S. K. Pillai B. (2007). A role for voltage-dependent anion channel Vdac1 in polyglutamine-mediated neuronal cell death. *PLoS ONE* 2 e1170 10.1371/journal.pone.0001170 PMC2064964 18000542

[B146] Giacomello M. Drago I. Pizzo P. Pozzan T. (2007). Mitochondrial Ca^2+^ as a key regulator of cell life and death. *Cell Death Differ.* 14 1267–1274 17431419 10.1038/sj.cdd.4402147

[B147] Gincel D. Shoshan-Barmatz V. (2004). Glutamate interacts with VDAC and modulates opening of the mitochondrial permeability transition pore. *J. Bioenerg. Biomembr.* 36 179–186 15224967 10.1023/b:jobb.0000023621.72873.9e

[B148] Gincel D. Silberberg S. D. Shoshan-Barmatz V. (2000). Modulation of the voltage-dependent anion channel (VDAC) by glutamate. *J. Bioenerg. Biomembr.* 32 571–583 15254371 10.1023/a:1005670527340

[B149] Gincel D. Zaid H. Shoshan-Barmatz V. (2001). Calcium binding and translocation by the voltage-dependent anion channel: a possible regulatory mechanism in mitochondrial function. *Biochem. J.* 358(Pt 1) 147–155 11485562 10.1042/0264-6021:3580147 PMC1222042

[B150] Godbole A. Varghese J. Sarin A. Mathew M. K. (2003). VDAC is a conserved element of death pathways in plant and animal systems. *Biochim. Biophys. Acta* 1642 87–96 12972297 10.1016/s0167-4889(03)00102-2

[B151] Gogvadze V. Orrenius S. Zhivotovsky B. (2006). Multiple pathways of cytochrome *c* release from mitochondria in apoptosis. *Biochim. Biophys. Acta* 1757 639–647 16678785 10.1016/j.bbabio.2006.03.016

[B152] Gogvadze V. Zhivotovsky B. Orrenius S. (2010). The Warburg effect and mitochondrial stability in cancer cells. *Mol. Aspects Med.* 31 60–74 19995572 10.1016/j.mam.2009.12.004

[B153] Golani I. Weizman A. Leschiner S. Spanier I. Eckstein N. Limor R. (2001). Hormonal regulation of peripheral benzodiazepine receptor binding properties is mediated by subunit interaction. *Biochemistry* 40 10213–10222 11513599 10.1021/bi010431+

[B154] Goldin N. Arzoine L. Heyfets A. Israelson A. Zaslavsky Z. Bravman T. (2008). Methyl jasmonate binds to and detaches mitochondria-bound hexokinase. *Oncogene* 27 4636–4643 18408762 10.1038/onc.2008.108

[B155] Goncalves R. P. Buzhynskyy N. Prima V. Sturgis J. N. Scheuring S. (2007). Supramolecular assembly of VDAC in native mitochondrial outer membranes. *J. Mol. Biol.* 369 413–418 17439818 10.1016/j.jmb.2007.03.063

[B156] Gonzalez-Gronow M. Kalfa T. Johnson C. E. Gawdi G. Pizzo S. V. (2003). The voltage-dependent anion channel is a receptor for plasminogen kringle 5 on human endothelial cells. *J. Biol. Chem.* 278 27312–27318 12736244 10.1074/jbc.M303172200

[B157] Gottlob K. Majewski N. Kennedy S. Kandel E. Robey R. B. Hay N. (2001). Inhibition of early apoptotic events by Akt/PKB is dependent on the first committed step of glycolysis and mitochondrial hexokinase. *Genes Dev.* 15 1406–1418 11390360 10.1101/gad.889901 PMC312709

[B158] Green D. R. Evan G. I. (2002). A matter of life and death. *Cancer Cell* 1 19–30 12086884 10.1016/s1535-6108(02)00024-7

[B159] Grills C. Jithesh P. V. Blayney J. Zhang S. D. Fennell D. A. (2011). Gene expression meta-analysis identifies VDAC1 as a predictor of poor outcome in early stage non-small cell lung cancer. *PLoS ONE* 6 e14635 10.1371/journal.pone.0014635 PMC3031508 21297950

[B160] Gross A. Jockel J. Wei M. C. Korsmeyer S. J. (1998). Enforced dimerization of BAX results in its translocation, mitochondrial dysfunction and apoptosis. *EMBO J.* 17 3878–3885 9670005 10.1093/emboj/17.14.3878 PMC1170723

[B161] Gunter T. E. Buntinas L. Sparagna G. Eliseev R. Gunter K. (2000). Mitochondrial calcium transport: mechanisms and functions. *Cell Calcium* 28 285–296 11115368 10.1054/ceca.2000.0168

[B162] Guo L. Pietkiewicz D. Pavlov E. V. Grigoriev S. M. Kasianowicz J. J. Dejean L. M. (2004). Effects of cytochrome c on the mitochondrial apoptosis-induced channel MAC. *Am. J. Physiol. Cell Physiol.* 286 C1109–C1117 15075210 10.1152/ajpcell.00183.2003

[B163] Guo X. W. Smith P. R. Cognon B. D’Arcangelis D. Dolginova E. Mannella C. A. (1995). Molecular design of the voltage-dependent, anion-selective channel in the mitochondrial outer membrane. *J. Struct. Biol.* 114 41–59 7772417 10.1006/jsbi.1995.1004

[B164] Gurnev P. A. Queralt-Martin M. Aguilella V. M. Rostovtseva T. K. Bezrukov S. M. (2012). Probing tubulin-blocked state of VDAC by varying membrane surface charge. *Biophys. J.* 102 2070–2076 22824270 10.1016/j.bpj.2012.03.058 PMC3341544

[B165] Hajnoczky G. Davies E. Madesh M. (2003). Calcium signaling and apoptosis. *Biochem. Biophys. Res. Commun.* 304 445–454 12729578 10.1016/s0006-291x(03)00616-8

[B166] Halestrap A. P. Green D. R. (2000). Mitochondria and cell death. *Biochem. Soc. Trans.* 28 170–177 10816121 10.1042/bst0280170

[B167] Halestrap A. P. McStay G. P. Clarke S. J. (2002). The permeability transition pore complex: another view. *Biochimie* 84 153–166 12022946 10.1016/s0300-9084(02)01375-5

[B168] Hall A. (2009). The cytoskeleton and cancer. *Cancer Metastasis Rev.* 28 5–14 19153674 10.1007/s10555-008-9166-3

[B169] Hamanaka R. B. Chandel N. S. (2009). Mitochondrial reactive oxygen species regulate hypoxic signaling. *Curr. Opin. Cell Biol.* 21 894–899 19781926 10.1016/j.ceb.2009.08.005 PMC2787901

[B170] Han D. Antunes F. Canali R. Rettori D. Cadenas E. (2003). Voltage-dependent anion channels control the release of the superoxide anion from mitochondria to cytosol. *J. Biol. Chem.* 278 5557–5563 12482755 10.1074/jbc.M210269200

[B171] Hanahan D. Weinberg R. A. (2000). The hallmarks of cancer. *Cell* 100 57–70 10647931 10.1016/s0092-8674(00)81683-9

[B172] Hanahan D. Weinberg R. A. (2011). Hallmarks of cancer: the next generation. *Cell* 144 646–674 21376230 10.1016/j.cell.2011.02.013

[B173] Haridas V. Li X. Mizumachi T. Higuchi M. Lemeshko V. V. Colombini M. (2007). Avicins, a novel plant-derived metabolite lowers energy metabolism in tumor cells by targeting the outer mitochondrial membrane. *Mitochondrion* 7 234–240 17317337 10.1016/j.mito.2006.12.005

[B174] Haridas V. Sultana R. Piroddi M. Cai J. Pierce W. M. Klein J. B. (2005). Avicinylation (thioesterification): a protein modification that can regulate the response to oxidative and nitrosative stress. *Proc. Natl. Acad. Sci. U.S.A.* 102 10088–10093 16030151 10.1073/pnas.0504430102 PMC1177405

[B175] Haworth R. A. Hunter D. R. (2000). Control of the mitochondrial permeability transition pore by high-affinity ADP binding at the ADP/ATP translocase in permeabilized mitochondria. *J. Bioenerg. Biomembr.* 32 91–96 11768766 10.1023/a:1005568630151

[B176] He L. Lemasters J. J. (2003). Heat shock suppresses the permeability transition in rat liver mitochondria. *J. Biol. Chem.* 278 16755–16760 12611884 10.1074/jbc.M300153200

[B177] Hickman J. A. (2002). Apoptosis and tumourigenesis. *Curr. Opin. Genet. Dev.* 12 67–72 11790557 10.1016/s0959-437x(01)00266-0

[B178] Hiller S. Garces R. G. Malia T. J. Orekhov V. Y. Colombini M. Wagner G. (2008). Solution structure of the integral human membrane protein VDAC-1 in detergent micelles. *Science* 321 1206–1210 18755977 10.1126/science.1161302 PMC2579273

[B179] Hinsch K. D. De Pinto V. Aires V. A. Schneider X. Messina A. Hinsch E. (2004). Voltage-dependent anion-selective channels VDAC2 and VDAC3 are abundant proteins in bovine outer dense fibers, a cytoskeletal component of the sperm flagellum. *J. Biol. Chem.* 279 15281–15288 14739283 10.1074/jbc.M313433200

[B180] Hirsch T. Decaudin D. Susin S. A. Marchetti P. Larochette N. (1998). PK11195, a ligand of the mitochondrial benzodiazepine receptor, facilitates the induction of apoptosis and reverses Bcl-2-mediated cytoprotection. *Exp. Cell Res.* 241 426–434 9637784 10.1006/excr.1998.4084

[B181] Holmuhamedov E. Lemasters J. J. (2009). Ethanol exposure decreases mitochondrial outer membrane permeability in cultured rat hepatocytes. *Arch. Biochem. Biophys.* 481 226–233 19014900 10.1016/j.abb.2008.10.036 PMC2656607

[B182] Hoogenboom B. W. Suda K. Engel A. Fotiadis D. (2007). The supramolecular assemblies of voltage-dependent anion channels in the native membrane. *J. Mol. Biol.* 370 246–255 17524423 10.1016/j.jmb.2007.04.073

[B183] Hou Q. Cymbalyuk E. Hsu S. C. Xu M. Hsu Y. T. (2003). Apoptosis modulatory activities of transiently expressed Bcl-2: roles in cytochrome *c* release and Bax regulation. *Apoptosis* 8 617–629 14739607 10.1023/A:1026187526113

[B184] Irusta P. M. Chen Y. B. Hardwick J. M. (2003). Viral modulators of cell death provide new links to old pathways. *Curr. Opin. Cell Biol.* 15 700–705 14644194 10.1016/j.ceb.2003.10.007

[B185] Isaacs J. S. Xu W. Neckers L. (2003). Heat shock protein 90 as a molecular target for cancer therapeutics. *Cancer Cell* 3 213–217 12676580 10.1016/s1535-6108(03)00029-1

[B186] Israelson A. Zaid H. Abu-Hamad S. Nahon E. Shoshan-Barmatz V. (2008). Mapping the ruthenium red-binding site of the voltage-dependent anion channel-1. *Cell Calcium* 43 196–204 17590433 10.1016/j.ceca.2007.05.006

[B187] Izard C. Libermann C. (1978). Acrolein. *Mutat. Res.* 47 115–138 415230 10.1016/0165-1110(78)90016-7

[B188] Jenei Z. A. Borthwick K. Zammit V. A. Dixon A. M. (2009). Self-association of transmembrane domain 2 (TM2), but not TM1, in carnitine palmitoyltransferase 1A: role of GXXXG(A) motifs. *J. Biol. Chem.* 284 6988–6997 19136561 10.1074/jbc.M808487200 PMC2652319

[B189] Jiang D. Zhao L. Clapham D. E. (2009). Genome-wide RNAi screen identifies Letm1 as a mitochondrial Ca^2+^/H^+^ antiporter. *Science* 326 144–147 19797662 10.1126/science.1175145 PMC4067766

[B190] Jiang N. Kham S. K. Koh G. S. Suang Lim J. Y. Ariffin H. Chew F. T. (2011). Identification of prognostic protein biomarkers in childhood acute lymphoblastic leukemia (ALL). *J. Proteomics* 74 843–157 21396490 10.1016/j.jprot.2011.02.034

[B191] Johnstone R. W. Ruefli A. A. Lowe S. W. (2002). Apoptosis: a link between cancer genetics and chemotherapy. *Cell* 108 153–164 11832206 10.1016/s0092-8674(02)00625-6

[B192] Jung J. Y. Han C. R. Jeong Y. J. Kim H. J. Lim H. S. Lee K. H. (2007). Epigallocatechin gallate inhibits nitric oxide-induced apoptosis in rat PC12 cells. *Neurosci. Lett.* 411 222–227 17116366 10.1016/j.neulet.2006.09.089

[B193] Karamouzis M. V. Moschos S. J. (2009). The use of endostatin in the treatment of solid tumors. *Expert Opin. Biol. Ther.* 9 641–648 19368526 10.1517/14712590902882118

[B194] Kayser H. Kratzin H. D. Thinnes F. P. Götz H. Schmidt W. E. Eckart K. (1989). Identification of human porins. II. Characterization and primary structure of a 31-lDa porin from human B lymphocytes (Porin 31HL). *Biol. Chem. Hoppe Seyler* 370 1265–1278 2559745

[B195] Keeble J. A. Gilmore A. P. (2007). Apoptosis commitment – translating survival signals into decisions on mitochondria. *Cell Res*. 17 976–984 18071367 10.1038/cr.2007.101

[B196] Keinan N. Tyomkin D. Shoshan-Barmatz V. (2010). Oligomerization of the mitochondrial protein voltage-dependent anion channel is coupled to the induction of apoptosis. *Mol. Cell. Biol.* 30 5698–5709 20937774 10.1128/MCB.00165-10 PMC3004265

[B197] Kerner J. Lee K. Tandler B. Hoppel C. L. (2012). VDAC proteomics: post-translation modifications. *Biochim. Biophys. Acta* 1818 1520–1525 22120575 10.1016/j.bbamem.2011.11.013 PMC4120668

[B198] Kim S. C. Sprung R. Chen Y. Xu Y. Ball H. Pei J. (2006). Substrate and functional diversity of lysine acetylation revealed by a proteomics survey. *Mol. Cell* 23 607–618 16916647 10.1016/j.molcel.2006.06.026

[B199] Kim Y. M. Bombeck C. A. Billiar T. R. (1999). Nitric oxide as a bifunctional regulator of apoptosis. *Circ. Res.* 84 253–256 10024298 10.1161/01.res.84.3.253

[B200] Kim Y. M. Kim T. H. Seol D. W. Talanian R. V. Billiar T. R. (1998). Nitric oxide suppression of apoptosis occurs in association with an inhibition of Bcl-2 cleavage and cytochrome c release. *J. Biol. Chem.* 273 31437–31441 9813055 10.1074/jbc.273.47.31437

[B201] Kinnally K. W. Peixoto P. M. Ryu S. Y. Dejean L. M. (2011). Is mPTP the gatekeeper for necrosis, apoptosis, or both? *Biochim. Biophys. Acta* 1813 616–622 20888866 10.1016/j.bbamcr.2010.09.013 PMC3050112

[B202] Ko Y. H. McFadden B. A. (1990). Alkylation of isocitrate lyase from *Escherichia coli* by 3-bromopyruvate. *Arch. Biochem. Biophys.* 278 373–380 2183722 10.1016/0003-9861(90)90273-2

[B203] Ko Y. H. Pedersen P. L. Geschwind J. F. (2001). Glucose catabolism in the rabbit VX2 tumor model for liver cancer: characterization and targeting hexokinase. *Cancer Lett.* 173 83–91 11578813 10.1016/s0304-3835(01)00667-x

[B204] Ko Y. H. Verhoeven H. A. Lee M. J. Corbin D. J. Vogl T. J. Pedersen P. L. (2012). A translational study “case report” on the small molecule “energy blocker” 3-bromopyruvate (3BP) as a potent anticancer agent: from bench side to bedside. *J. Bioenerg. Biomembr.* 44 163–170 22328020 10.1007/s10863-012-9417-4

[B205] Kokoszka J. E. Waymire K. G. Levy S. E. Sligh J. E. Cai J. Jones D. P. (2004). The ADP/ATP translocator is not essential for the mitochondrial permeability transition pore. *Nature* 427 461–465 14749836 10.1038/nature02229 PMC3049806

[B206] Koppenol W. H. Bounds P. L. Dang C. V. (2011). Otto Warburg’s contributions to current concepts of cancer metabolism. *Nat. Rev. Cancer* 11 325–337 21508971 10.1038/nrc3038

[B207] Koren I. Raviv Z. Shoshan-Barmatz V. (2010). Downregulation of voltage-dependent anion channel-1 expression by RNA interference prevents cancer cell growth in vivo. *Cancer Biol. Ther.* 9 1046–1052 20404552 10.4161/cbt.9.12.11879

[B208] Korkhov V. M. Sachse C. Short J. M. Tate C. G. (2010). Three-dimensional structure of TspO by electron cryomicroscopy of helical crystals. *Structure* 18 677–687 20541505 10.1016/j.str.2010.03.001 PMC2911597

[B209] Korsmeyer S. J. Wei M. C. Saito M. Weiler S. Oh K. J. Schlesinger P. H. (2000). Pro-apoptotic cascade activates BID, which oligomerizes BAK or BAX into pores that result in the release of cytochrome. *c. Cell Death Differ.* 7 1166–1173 10.1038/sj.cdd.4400783 11175253

[B210] Krauskopf A. Eriksson O. Craigen W. J. Forte M. A. Bernardi P. (2006). Properties of the permeability transition in VDAC1(–/–) mitochondria. *Biochim. Biophys. Acta* 1757 590–595 16626625 10.1016/j.bbabio.2006.02.007

[B211] Kroemer G. (1997). The proto-oncogene Bcl-2 and its role in regulating apoptosis. *Nat. Med.* 3 614–620 9176486 10.1038/nm0697-614

[B212] Kroemer G. Galluzzi L. Brenner C. (2007). Mitochondrial membrane permeabilization in cell death. *Physiol. Rev.* 87 99–163 17237344 10.1152/physrev.00013.2006

[B213] Kroemer G. Pouyssegur J. (2008). Tumor cell metabolism: cancer’s Achilles’ heel. *Cancer Cell* 13 472–482 18538731 10.1016/j.ccr.2008.05.005

[B214] Kugler W. Veenman L. Shandalov Y. Leschiner S. Spanier I. Lakomek M. (2008). Ligands of the mitochondrial 18 kDa translocator protein attenuate apoptosis of human glioblastoma cells exposed to erucylphosphohomocholine. *Cell. Oncol.* 30 435–450 18791274 10.3233/CLO-2008-0431 PMC4618834

[B215] Kusano H. Shimizu S. Koya R. C. Fujita H. Kamada S. Kuzumaki N. (2000). Human gelsolin prevents apoptosis by inhibiting apoptotic mitochondrial changes via closing VDAC. *Oncogene* 19 4807–4814 11039896 10.1038/sj.onc.1203868

[B216] Kuwana T. Mackey M. R. Perkins G. Ellisman M. H. Latterich M. Schneiter R. (2002). Bid, Bax, and lipids cooperate to form supramolecular openings in the outer mitochondrial membrane. *Cell* 111 331–342 12419244 10.1016/s0092-8674(02)01036-x

[B217] Lai J. C. Tan W. Benimetskaya L. Miller P. Colombini M. Stein C. A. (2006). A pharmacologic target of G3139 in melanoma cells may be the mitochondrial VDAC. *Proc. Natl. Acad. Sci. U.S.A.* 103 7494–7499 16648253 10.1073/pnas.0602217103 PMC1464367

[B218] Larochette N. Decaudin D. Jacotot E. Brenner C. Marzo I. Susin S. A. (1999). Arsenite induces apoptosis via a direct effect on the mitochondrial permeability transition pore. *Exp. Cell Res.* 249 413–421 10366441 10.1006/excr.1999.4519

[B219] Le Bras M. Clément M. V. Pervaiz S. Brenner C. (2005). Reactive oxygen species and the mitochondrial signaling pathway of cell death. *Histol. Histopathol.* 20 205–219 15578439 10.14670/HH-20.205

[B220] Lee A. C. Zizi M. Colombini M. (1994). Beta-NADH decreases the permeability of the mitochondrial outer membrane to ADP by a factor of 6. *J. Biol. Chem.* 269 30974–30980 7983033

[B221] Lee B. Miles P. D. Vargas L. Luan P. Glasco S. Kushnareva Y. (2003). Inhibition of mitochondrial Na^+^-Ca^2+^ exchanger increases mitochondrial metabolism and potentiates glucose-stimulated insulin secretion in rat pancreatic islets. *Diabetes* 52 965–973 12663468 10.2337/diabetes.52.4.965

[B222] Lee C. S. Kim Y. J. Jang E. R. Kim W. Myung S. C. (2010). Fluoxetine induces apoptosis in ovarian carcinoma cell line OVCAR-3 through reactive oxygen species-dependent activation of nuclear factor-kappaB. *Basic Clin. Pharmacol. Toxicol.* 106 446–453 20050848 10.1111/j.1742-7843.2009.00509.x

[B223] Lee H. J. Kim J. W. Yim S. V. Kim M. J. Kim S. A. Kim Y. J. (2001). Fluoxetine enhances cell proliferation and prevents apoptosis in dentate gyrus of maternally separated rats. *Mol. Psychiatry* 6 725–728 10.1038/sj.mp.4000954 11673802

[B224] Lemasters J. J. Holmuhamedov E. (2006). Voltage-dependent anion channel (VDAC) as mitochondrial governator – thinking outside the box. *Biochim. Biophys. Acta* 1762 181–190 16307870 10.1016/j.bbadis.2005.10.006

[B225] Lemasters J. J. Qian T. Bradham C. A. Brenner D. A. Cascio W. E. Trost L. C. (1999). Mitochondrial dysfunction in the pathogenesis of necrotic and apoptotic cell death. *J. Bioenerg. Biomembr.* 31 305–319 10665521 10.1023/a:1005419617371

[B226] Le Mellay V. Troppmair J. Benz R. Rapp U. R. (2002). Negative regulation of mitochondrial VDAC channels by C-Raf kinase. *BMC Cell Biol.* 3 14 10.1186/1471-2121-3-14 PMC116581 12079506

[B227] Lemeshko V. V. Haridas V. Quijano Pérez J. C. Gutterman J. U. (2006). Avicins, natural anticancer saponins, permeabilize mitochondrial membranes. *Arch. Biochem. Biophys.* 454 114–122 16962987 10.1016/j.abb.2006.08.008

[B228] Levin E. Premkumar A. Veenman L. Kugler W. Leschiner S. Spanier I. (2005). The peripheral-type benzodiazepine receptor and tumorigenicity: isoquinoline binding protein (IBP) antisense knockdown in the C6 glioma cell line. *Biochemistry* 44 9924–9935 16026165 10.1021/bi050150s

[B229] Li J. Bombeck C. A. Yang S. Kim Y. M. Billiar T. R. (1999). Nitric oxide suppresses apoptosis via interrupting caspase activation and mitochondrial dysfunction in cultured hepatocytes. *J. Biol. Chem.* 274 17325–17333 10358093 10.1074/jbc.274.24.17325

[B230] Li J. Yuan J. (2008). Caspases in apoptosis and beyond. *Oncogene* 27 6194–6206 18931687 10.1038/onc.2008.297

[B231] Li M. Xiao Z. Q. Chen Z. C. Li J. L. Li C Zhang P. F. (2007). Proteomic analysis of the aging-related proteins in human normal colon epithelial tissue. *J. Biochem. Mol. Biol.* 40 72–81 17244485 10.5483/bmbrep.2007.40.1.072

[B232] Lindenboim L. Kringel S. Braun T. Borner C. Stein R. (2005). Bak but not Bax is essential for Bcl-xS-induced apoptosis. *Cell Death Differ.* 12 713–723 15861188 10.1038/sj.cdd.4401638

[B233] Liu B. Wang P. Wang Z. Zhang W. (2011). The use of anti-VDAC2 antibody for the combined assessment of human sperm acrosome integrity and ionophore A23187-induced acrosome reaction. *PLoS ONE* 6 e16985 10.1371/journal.pone.0016985 PMC3036732 21347391

[B234] Liu S. Ishikawa H. Tsuyama N. Li F. J. Abroun S. Otsuyama K. I. (2006). Increased susceptibility to apoptosis in CD45(+) myeloma cells accompanied by the increased expression of VDAC1. *Oncogene* 25 419–429 16247487 10.1038/sj.onc.1208982

[B235] Llambi F. Green D. R. (2011). Apoptosis and oncogenesis: give and take in the BCL-2 family. *Curr. Opin. Genet. Dev.* 21 12–20 21236661 10.1016/j.gde.2010.12.001 PMC3040981

[B236] Lovell J. F. Billen L. P. Bindner S. Shamas-Din A. Fradin C. Leber B. (2008). Membrane binding by tBid initiates an ordered series of events culminating in membrane permeabilization by Bax. *Cell* 135 1074–1084 19062087 10.1016/j.cell.2008.11.010

[B237] Lü A. J. Dong C. W. Du C. S. Zhang Q. Y. (2007). Characterization and expression analysis of Paralichthys olivaceus voltage-dependent anion channel (VDAC) gene in response to virus infection. *Fish Shellfish Immunol.* 23 601–613 17467295 10.1016/j.fsi.2007.01.007

[B238] Luo J. Uchida K. Shi R. (2005). Accumulation of acrolein-protein adducts after traumatic spinal cord injury. *Neurochem. Res.* 30 291–295 16018572 10.1007/s11064-005-2602-7

[B239] Maack C. Cortassa S. Aon M. A. Ganesan A. N. Liu T O’Rourke B. (2006). Elevated cytosolic Na^+^ decreases mitochondrial Ca^2+^ uptake during excitation-contraction coupling and impairs energetic adaptation in cardiac myocytes. *Circ. Res.* 99 172–182 16778127 10.1161/01.RES.0000232546.92777.05 PMC2711867

[B240] Mader A. Abu-Hamad S. Arbel N. Gutiérrez-Aguilar M. Shoshan-Barmatz V. (2010). Dominant-negative VDAC1 mutants reveal oligomeric VDAC1 to be the active unit in mitochondria-mediated apoptosis. *Biochem. J.* 429 147–155 20420578 10.1042/BJ20091338

[B241] Madesh M. Hajnoczky G. (2001). VDAC-dependent permeabilization of the outer mitochondrial membrane by superoxide induces rapid and massive cytochrome c release. *J. Cell Biol.* 155 1003–1015 11739410 10.1083/jcb.200105057 PMC2150912

[B242] Maechler P. Kennedy E. D. Pozzan T. Wollheim C. B. (1997). Mitochondrial activation directly triggers the exocytosis of insulin in permeabilized pancreatic beta-cells. *EMBO J.* 16 3833–3841 9233793 10.1093/emboj/16.13.3833 PMC1170007

[B243] Majewski N. Nogueira V. Bhaskar P. Coy P. E. Skeen J. E. Gottlob K. (2004a). Hexokinase-mitochondria interaction mediated by Akt is required to inhibit apoptosis in the presence or absence of Bax and Bak. *Mol. Cell* 16 819–830 15574336 10.1016/j.molcel.2004.11.014

[B244] Majewski N. Nogueira V. Robey R. B. Hay N. (2004b). Akt inhibits apoptosis downstream of BID cleavage via a glucose-dependent mechanism involving mitochondrial hexokinases. *Mol. Cell. Biol.* 24 730–740 14701745 10.1128/MCB.24.2.730-740.2004 PMC343797

[B245] Malia T. J. Wagner G. (2007). NMR structural investigation of the mitochondrial outer membrane protein VDAC and its interaction with antiapoptotic Bcl-xL. *Biochemistry* 46 514–525 17209561 10.1021/bi061577h PMC2579276

[B246] Manda G. Nechifor M. T. Neagu T.-M. (2009). Reactive oxygen species, cancer and anti-cancer therapies. *Curr. Chem. Biol.* 3 342–366

[B247] Marchetti P. Zamzami N. Joseph B. Schraen-Maschke S. Méreau-Richard C. Costantini P. (1999). The novel retinoid 6-[3-(1-adamantyl)-4-hydroxyphenyl]-2-naphtalene carboxylic acid can trigger apoptosis through a mitochondrial pathway independent of the nucleus. *Cancer Res.* 59 6257–6266 10626821

[B248] Martel C. Allouche M. Esposti D. D. Fanelli E. Boursier C. Henry C. (2012). GSK3-mediated VDAC phosphorylation controls outer mitochondrial membrane permeability during lipid accumulation. *Hepatology*. 10.1002/hep.25967 [Epub ahead of print]. 22814966

[B249] Martinez-Abundis E. Correa F. Pavón N. Zazueta C. (2009). Bax distribution into mitochondrial detergent-resistant microdomains is related to ceramide and cholesterol content in postischemic hearts. *FEBS J.* 276 5579–5588 19694802 10.1111/j.1742-4658.2009.07239.x

[B250] Martinez-Caballero S. Dejean L. M. Jonas E. A. Kinnally K. W. (2005). The role of the mitochondrial apoptosis induced channel MAC in cytochrome c release. *J. Bioenerg. Biomembr.* 37 155–164 16167172 10.1007/s10863-005-6570-z

[B251] Martinez-Caballero S. Dejean L. M. Kinnally K. W. (2004). Some amphiphilic cations block the mitochondrial apoptosis-induced channel, MAC. *FEBS Lett.* 568 35–38 15196916 10.1016/j.febslet.2004.05.006

[B252] Martinez-Caballero S. Dejean L. M. Kinnally M. S. Oh K. J. Mannella C. A. Kinnally K. W. (2009). Assembly of the mitochondrial apoptosis-induced channel, MAC. *J. Biol. Chem.* 284 12235–12245 19261612 10.1074/jbc.M806610200 PMC2673292

[B253] Mathupala S. P. Ko Y. H. Pedersen P. L. (2006). Hexokinase II: cancer’s double-edged sword acting as both facilitator and gatekeeper of malignancy when bound to mitochondria. *Oncogene* 25 4777–4786 16892090 10.1038/sj.onc.1209603 PMC3385868

[B254] Mathupala S. P. Ko Y. H. Pedersen P. L. (2010). The pivotal roles of mitochondria in cancer: Warburg and beyond and encouraging prospects for effective therapies. *Biochim. Biophys. Acta* 1797 1225–1230 20381449 10.1016/j.bbabio.2010.03.025 PMC2890051

[B255] Mayevsky A. (2009). Mitochondrial function and energy metabolism in cancer cells: past overview and future perspectives. *Mitochondrion* 9 165–179 19460294 10.1016/j.mito.2009.01.009

[B256] McEnery M. W. (1992). The mitochondrial benzodiazepine receptor: evidence for association with the voltage-dependent anion channel (VDAC). *J. Bioenerg. Biomembr.* 24 63–69 1380506 10.1007/BF00769532

[B257] Mello C. F. Sultana R. Piroddi M. Cai J. Pierce W. M. Klein J. B. (2007). Acrolein induces selective protein carbonylation in synaptosomes. *Neuroscience* 147 674–679 17570602 10.1016/j.neuroscience.2007.04.003 PMC1987324

[B258] Meloche H. P. Monti C. T. (1975). Bromopyruvate inactivation of 2-keto-3-deoxy-6-phosphogalactonate aldolase of *Pseudomonas saccharophila*. Kinetics and stereochemistry. *Biochemistry* 14 3682–3687 1164502 10.1021/bi00687a026

[B259] Menzel V. A. Cassará M. C. Benz R. de Pinto V. Messina A. Cunsolo V. (2009). Molecular and functional characterization of VDAC2 purified from mammal spermatozoa. *Biosci. Rep.* 29 351–362 18976238 10.1042/BSR20080123

[B260] Messina A. Reina S. Guarino F De Pinto V. (2012). VDAC isoforms in mammals. *Biochim. Biophys. Acta* 1818 1466–1476 22020053 10.1016/j.bbamem.2011.10.005

[B261] Mikhailov V. Mikhailova M. Degenhardt K. Venkatachalam M. A. White E. Saikumar P. (2003). Association of Bax and Bak homo-oligomers in mitochondria. Bax requirement for Bak reorganization and cytochrome *c* release. *J. Biol. Chem.* 278 5367–5376 12454021 10.1074/jbc.M203392200

[B262] Miura N. Takemori N. Kikugawa T. Tanji N. Higashiyama S. Yokoyama M. (2012). Adseverin: a novel cisplatin-resistant marker in the human bladder cancer cell line HT1376 identified by quantitative proteomic analysis. *Mol. Oncol.* 6 311–322 22265592 10.1016/j.molonc.2011.12.002 PMC5528333

[B263] Miyashita T. Reed J. C. (1993). Bcl-2 oncoprotein blocks chemotherapy-induced apoptosis in a human leukemia cell line. *Blood* 81 151–157 8417786

[B264] Mizuta T. Shimizu S. Matsuoka Y. Nakagawa T. Tsujimoto Y. (2007). A Bax/Bak-independent mechanism of cytochrome c release. *J. Biol. Chem.* 282 16623–16630 17409097 10.1074/jbc.M611060200

[B265] Mlayeh L. Chatkaew S. Léonetti M Homblé F. (2010). Modulation of plant mitochondrial VDAC by phytosterols. *Biophys. J.* 99 2097–2106 20923643 10.1016/j.bpj.2010.07.067 PMC3042562

[B266] Moin S. M. Panteva M. Jameel S. (2007). The hepatitis E virus Orf3 protein protects cells from mitochondrial depolarization and death. *J. Biol. Chem.* 282 21124–21133 17488721 10.1074/jbc.M701696200 PMC2440810

[B267] Murphy M. P. (2009). How mitochondria produce reactive oxygen species. *Biochem. J.* 417 1–13 19061483 10.1042/BJ20081386 PMC2605959

[B268] Nahon E. Israelson A. Abu-Hamad S. Varda S. B. (2005). Fluoxetine (Prozac) interaction with the mitochondrial voltage-dependent anion channel and protection against apoptotic cell death. *FEBS Lett.* 579 5105–5110 16139271 10.1016/j.febslet.2005.08.020

[B269] Nawarak J. Huang-Liu R. Kao S. H. Liao H. H. Sinchaikul S. Chen S. T. (2009). Proteomics analysis of A375 human malignant melanoma cells in response to arbutin treatment. *Biochim. Biophys. Acta* 1794 159–167 18996230 10.1016/j.bbapap.2008.09.023

[B270] Neumann D. Bückers J. Kastrup L. Hell S. W. Jakobs S. (2010). Two-color STED microscopy reveals different degrees of colocalization between hexokinase-I and the three human VDAC isoforms. *PMC Biophys.* 3 4 10.1186/1757-5036-3-4 PMC2838807 20205711

[B271] Nichols B. J. Denton R. M. (1995). Towards the molecular basis for the regulation of mitochondrial dehydrogenases by calcium ions. *Mol. Cell. Biochem.* 149–150 203–212 10.1007/BF01076578 8569730

[B272] Nogueira V. Park Y. Chen C. C. Xu P. Z. Chen M. L. Tonic I. (2008). Akt determines replicative senescence and oxidative or oncogenic premature senescence and sensitizes cells to oxidative apoptosis. *Cancer Cell* 14 458–470 19061837 10.1016/j.ccr.2008.11.003 PMC3038665

[B273] Oancea M. Mani A. Hussein M. A. Almasan A. (2004). Apoptosis of multiple myeloma. *Int. J. Hematol.* 80 224–231 15540896 10.1532/IJH97.04107 PMC1193518

[B274] Oh H. L. Seok J. Y. Kwon C. H. Kang S. K. Kim Y. K. (2006). Role of MAPK in ceramide-induced cell death in primary cultured astrocytes from mouse embryonic brain. *Neurotoxicology* 27 31–38 16143399 10.1016/j.neuro.2005.05.008

[B275] Okada S. F. O’Neal W. K. Huang P. Nicholas R. A. Ostrowski L. E. Craigen W. J. (2004). Voltage-dependent anion channel-1 (VDAC-1) contributes to ATP release and cell volume regulation in murine cells. *J. Gen. Physiol.* 124 513–526 15477379 10.1085/jgp.200409154 PMC2234005

[B276] Olson R. D. Boerth R. C. Gerber J. G. Nies A. S. (1981). Mechanism of adriamycin cardiotoxicity: evidence for oxidative stress. *Life Sci.* 29 1393–1401 7029182 10.1016/0024-3205(81)90001-1

[B277] Ospina A. Lagunas-Martínez A. Pardo J. Carrodeguas J. A. (2011). Protein oligomerization mediated by the transmembrane carboxyl terminal domain of Bcl-XL. *FEBS Lett.* 585 2935–2942 21856303 10.1016/j.febslet.2011.08.012 PMC7164028

[B278] Ott M. Gogvadze V. Orrenius S. Zhivotovsky B. (2007). Mitochondria, oxidative stress and cell death. *Apoptosis* 12 913–922 17453160 10.1007/s10495-007-0756-2

[B279] Ott M. Robertson J. D. Gogvadze V. Zhivotovsky B. Orrenius S. (2002). Cytochrome *c* release from mitochondria proceeds by a two-step process. *Proc. Natl. Acad. Sci. U.S.A.* 99 1259–1263 11818574 10.1073/pnas.241655498 PMC122177

[B280] Ozaki T. Yamashita T. Ishiguro S. (2009). Mitochondrial m-calpain plays a role in the release of truncated apoptosis-inducing factor from the mitochondria. *Biochim. Biophys. Acta* 1793 1848–1859 19833151 10.1016/j.bbamcr.2009.10.002

[B281] Palty R. Silverman W. F. Hershfinkel M. Caporale T. Sensi S. L. Parnis J. (2010). NCLX is an essential component of mitochondrial Na^+^/Ca^2+^ exchange. *Proc. Natl. Acad. Sci. U.S.A.* 107 436–441 20018762 10.1073/pnas.0908099107 PMC2806722

[B282] Pastorino J. G. Hoek J. B. (2008). Regulation of hexokinase binding to VDAC. *J. Bioenerg. Biomembr.* 40 171–182 18683036 10.1007/s10863-008-9148-8 PMC2662512

[B283] Pastorino J. G. Hoek J. B. Shulga N. (2005). Activation of glycogen synthase kinase 3beta disrupts the binding of hexokinase II to mitochondria by phosphorylating voltage-dependent anion channel and potentiates chemotherapy-induced cytotoxicity. *Cancer Res*. 65 10545–10554 16288047 10.1158/0008-5472.CAN-05-1925

[B284] Pastorino J. G. Shulga N. Hoek J. B. (2002). Mitochondrial binding of hexokinase II inhibits Bax-induced cytochrome c release and apoptosis. *J. Biol. Chem.* 277 7610–7618 11751859 10.1074/jbc.M109950200

[B285] Pastorino J. G. Simbula G. Gilfor E. Hoek J. B. Farber J. L. (1994). Protoporphyrin IX, an endogenous ligand of the peripheral benzodiazepine receptor, potentiates induction of the mitochondrial permeability transition and the killing of cultured hepatocytes by rotenone. *J. Biol. Chem.* 269 31041–31046 7983042

[B286] Pedersen P. L. (2007). Warburg, me and Hexokinase 2: multiple discoveries of key molecular events underlying one of cancers’ most common phenotypes, the “Warburg effect”, i.e., elevated glycolysis in the presence of oxygen. *J. Bioenerg. Biomembr.* 39 211–222 17879147 10.1007/s10863-007-9094-x

[B287] Pedersen P. L. (2008). Voltage dependent anion channels (VDACs): a brief introduction with a focus on the outer mitochondrial compartment’s roles together with hexokinase-2 in the “Warburg effect” in cancer. *J. Bioenerg. Biomembr.* 40 123–126 18780167 10.1007/s10863-008-9165-7

[B288] Pedersen P. L. Mathupala S. Rempel A. Geschwind J. F. Ko Y. H. (2002). Mitochondrial bound type II hexokinase: a key player in the growth and survival of many cancers and an ideal prospect for therapeutic intervention. *Biochim. Biophys. Acta* 1555 14–20 12206885 10.1016/s0005-2728(02)00248-7

[B289] Peixoto P. M. Dejean L. M. Kinnally K. W. (2012). The therapeutic potential of mitochondrial channels in cancer, ischemia-reperfusion injury, and neurodegeneration. *Mitochondrion* 12 14–23 21406252 10.1016/j.mito.2011.03.003 PMC3410559

[B290] Pelicano H. Feng L. Zhou Y. Carew J. S. Hileman E. O. Plunkett W. (2003). Inhibition of mitochondrial respiration: a novel strategy to enhance drug-induced apoptosis in human leukemia cells by a reactive oxygen species-mediated mechanism. *J. Biol. Chem.* 278 37832–37839 12853461 10.1074/jbc.M301546200

[B291] Penso J. Beitner R. (1998). Clotrimazole and bifonazole detach hexokinase from mitochondria of melanoma cells. *Eur. J. Pharmacol.* 342 113–117 9544799 10.1016/s0014-2999(97)01507-0

[B292] Pereira da Silva A. P. El-Bacha T. Kyaw N. dos Santos R. S. da-Silva W. S. Almeida F. C. (2009). Inhibition of energy-producing pathways of HepG2 cells by 3-bromopyruvate. *Biochem. J.* 417 717–726 18945211 10.1042/BJ20080805

[B293] Perfettini J. L. Castedo M. Roumier T. Andreau K. Nardacci R. Piacentini M. (2005). Mechanisms of apoptosis induction by the HIV-1 envelope. *Cell Death Differ.* 12(Suppl. 1) 916–923 15719026 10.1038/sj.cdd.4401584

[B294] Petrosillo G. Ruggiero F. M. Paradies G. (2003). Role of reactive oxygen species and cardiolipin in the release of cytochrome *c* from mitochondria. *FASEB J.* 17 2202–2208 14656982 10.1096/fj.03-0012com

[B295] Petrosillo G. Ruggiero F. M. Pistolese M. Paradies G. (2001). Reactive oxygen species generated from the mitochondrial electron transport chain induce cytochrome *c* dissociation from beef-heart submitochondrial particles via cardiolipin peroxidation. Possible role in the apoptosis. *FEBS Lett.* 509 435–438 11749969 10.1016/s0014-5793(01)03206-9

[B296] Pinton P. Giorgi C. Siviero R. Zecchini E. Rizzuto R. (2008). Calcium and apoptosis: ER–mitochondria Ca^2+^ transfer in the control of apoptosis. *Oncogene* 27 6407–6418 18955969 10.1038/onc.2008.308 PMC2844952

[B297] Priault M. Chaudhuri B. Clow A. Camougrand N. Manon S. (1999). Investigation of bax-induced release of cytochrome *c* from yeast mitochondria permeability of mitochondrial membranes, role of VDAC and ATP requirement. *Eur. J. Biochem.* 260 684–691 10102996 10.1046/j.1432-1327.1999.00198.x

[B298] Pro B. Leber B. Smith M. Fayad L. Romaguera J. Hagemeister F. (2008). Phase II multicenter study of oblimersen sodium, a Bcl-2 antisense oligonucleotide, in combination with rituximab in patients with recurrent B-cell non-Hodgkin lymphoma. *Br. J. Haematol.* 143 355–360 18764869 10.1111/j.1365-2141.2008.07353.x

[B299] Qiao H. McMillan J. R. (2007). Gelsolin segment 5 inhibits HIV-induced T-cell apoptosis via Vpr-binding to VDAC. *FEBS Lett.* 581 535–540 17254575 10.1016/j.febslet.2006.12.057

[B300] Rahmani Z. Huh K. W. Lasher R. Siddiqui A. (2000). Hepatitis B virus X protein colocalizes to mitochondria with a human voltage-dependent anion channel, HVDAC3, and alters its transmembrane potential. *J. Virol.* 74 2840–2846 10684300 10.1128/jvi.74.6.2840-2846.2000 PMC111774

[B301] Ralph S. J. Rodríguez-Enríquez S. Neuzil J. Saavedra E Moreno-Sánchez R. (2010). The causes of cancer revisited: “mitochondrial malignancy” and ROS-induced oncogenic transformation – why mitochondria are targets for cancer therapy. *Mol. Aspects Med.* 31 145–170 20206201 10.1016/j.mam.2010.02.008

[B302] Rapizzi E. Pinton P. Szabadkai G. Wieckowski M. R. Vandecasteele G. Baird G. (2002). Recombinant expression of the voltage-dependent anion channel enhances the transfer of Ca^2+^ microdomains to mitochondria. *J. Cell Biol.* 159 613–624 12438411 10.1083/jcb.200205091 PMC2173108

[B303] Rasola A. Bernardi P. (2011). Mitochondrial permeability transition in Ca(2+)-dependent apoptosis and necrosis. *Cell Calcium* 50 222–233 21601280 10.1016/j.ceca.2011.04.007

[B304] Ravagnan L. Marzo I. Costantini P. Susin S. A. Zamzami N. Petit P. X. (1999). Lonidamine triggers apoptosis via a direct, Bcl-2-inhibited effect on the mitochondrial permeability transition pore. *Oncogene* 18 2537–2546 10353597 10.1038/sj.onc.1202625

[B305] Reed J. C. (2006). Proapoptotic multidomain Bcl-2/Bax-family proteins: mechanisms, physiological roles, and therapeutic opportunities. *Cell Death Differ.* 13 1378–1386 16729025 10.1038/sj.cdd.4401975

[B306] Reina S. Palermo V. Guarnera A. Guarino F. Messina A. Mazzoni C. (2010). Swapping of the N-terminus of VDAC1 with VDAC3 restores full activity of the channel and confers anti-aging features to the cell. *FEBS Lett.* 584 2837–2844 20434446 10.1016/j.febslet.2010.04.066

[B307] Reymann S. Buzhynskyy N. Prima V. Sturgis J. N. Scheuring S. (1995). Further evidence for multitopological localization of mammalian porin (VDAC) in the plasmalemma forming part of a chloride channel complex affected in cystic fibrosis and encephalomyopathy. *Biochem. Mol. Med.* 54 75–87 8581362 10.1006/bmme.1995.1011

[B308] Rom J. von Minckwitz G. Marmé F. Ataseven B. Kozian D. Sievert M. (2009). Phase I study of apoptosis gene modulation with oblimersen within preoperative chemotherapy in patients with primary breast cancer. *Ann. Oncol.* 20 1829–1835 19605509 10.1093/annonc/mdp208

[B309] Romagnoli R. Baraldi P. G. Lopez-Cara C. Cruz-Lopez O. Carrion M. D. Kimatrai Salvador M. (2011). Synthesis and antitumor molecular mechanism of agents based on amino 2-(3′,4′,5′-trimethoxybenzoyl)benzo[b]furan: inhibition of tubulin and induction of apoptosis. *ChemMedChem* 6 1841–1853 21805646 10.1002/cmdc.201100279 PMC3190670

[B310] Roman I. Figys J. Steurs G. Zizi M. (2006). Direct measurement of VDAC-actin interaction by surface plasmon resonance. *Biochim. Biophys. Acta* 1758 479–486 16678788 10.1016/j.bbamem.2006.03.019

[B311] Rone M. B. Fan J. Papadopoulos V. (2009). Cholesterol transport in steroid biosynthesis: role of protein–protein interactions and implications in disease states. *Biochim. Biophys. Acta* 1791 646–658 19286473 10.1016/j.bbalip.2009.03.001 PMC2757135

[B312] Rosano C. (2011). Molecular model of hexokinase binding to the outer mitochondrial membrane porin (VDAC1): implication for the design of new cancer therapies. *Mitochondrion* 11 513–519 21315184 10.1016/j.mito.2011.01.012

[B313] Rose I. A. O’Connell E. L. Litwin S. (1974). Determination of the rate of hexokinase-glucose dissociation by the isotope-trapping method. *J. Biol. Chem.* 249 5163–5168 4604308

[B314] Rostovtseva T. K. Antonsson B. Suzuki M. Youle R. J. Colombini M. Bezrukov S. M. (2004). Bid, but not Bax, regulates VDAC channels. *J. Biol. Chem.* 279 13575–13583 14729675 10.1074/jbc.M310593200

[B315] Rostovtseva T. K. Bezrukov S. M. (2012). VDAC inhibition by tubulin and its physiological implications. *Biochim. Biophys. Acta* 1818 1526–1535 22100746 10.1016/j.bbamem.2011.11.004 PMC3302949

[B316] Rostovtseva T. K. Gurnev P. A. Chen M. Y. Bezrukov S. M. (2012). Membrane lipid composition regulates tubulin interaction with mitochondrial voltage-dependent anion channel. *J. Biol. Chem.* 287 29589–29598 22763701 10.1074/jbc.M112.378778 PMC3436136

[B317] Rostovtseva T. K. Komarov A. Bezrukov S. M. Colombini M. (2002). Dynamics of nucleotides in VDAC channels: structure-specific noise generation. *Biophys. J.* 82(Pt 1) 193–205 11751308 10.1016/S0006-3495(02)75386-1 PMC1302461

[B318] Rostovtseva T. K. Petrache H. I. Kazemi N. Hassanzadeh E. Bezrukov S. M. (2008a). Interfacial polar interactions affect gramicidin channel kinetics. *Biophys J.* 94 L23–L25 18055540 10.1529/biophysj.107.120261 PMC2212686

[B319] Rostovtseva T. K. Sheldon K. L. Hassanzadeh E. Monge C. Saks V. Bezrukov S. M. (2008b). Tubulin binding blocks mitochondrial voltage-dependent anion channel and regulates respiration. *Proc. Natl. Acad. Sci. U.S.A.* 105 18746–18751 19033201 10.1073/pnas.0806303105 PMC2596221

[B320] Saddar S. Stuart R. A. (2005). The yeast F(1)F(0)-ATP synthase: analysis of the molecular organization of subunit g and the importance of a conserved GXXXG motif. *J. Biol. Chem.* 280 24435–24442 15886192 10.1074/jbc.M502804200

[B321] Sahasrabudhe S. R. Lai S. Pierce M. Clemens C. Venkat R. Rebentisch M. (2008). Selective in vitro and in vivo anti-tumor activity of PRLX 93936 in biological models of melanoma and ovarian cancer. *J. Clin. Oncol.* 26(Suppl.) Abstr. 14586

[B322] Saks V. Guzun R. Timohhina N. Tepp K. Varikmaa M. Monge C. (2010). Structure–function relationships in feedback regulation of energy fluxes in vivo in health and disease: mitochondrial interactosome. *Biochim. Biophys. Acta* 1797 678–697 20096261 10.1016/j.bbabio.2010.01.011

[B323] Sampson M. J. Decker W. K. Beaudet A. L. Ruitenbeek W. Armstrong D. Hicks M. J. (2001). Immotile sperm and infertility in mice lacking mitochondrial voltage-dependent anion channel type 3. *J. Biol. Chem.* 276 39206–39212 11507092 10.1074/jbc.M104724200

[B324] Sampson M. J. Lovell R. S. Craigen W. J. (1997). The murine voltage-dependent anion channel gene family. Conserved structure and function. *J. Biol. Chem.* 272 18966–18973 9228078 10.1074/jbc.272.30.18966

[B325] Santin G. Piccolini V. M. Veneroni P. Barni S. Bernocchi G. Bottone M. G. (2011). Different patterns of apoptosis in response to cisplatin in B50 neuroblastoma rat cells. *Histol. Histopathol.* 26 831–842 21630213 10.14670/HH-26.831

[B326] Santo-Domingo J. Demaurex N. (2010). Calcium uptake mechanisms of mitochondria. *Biochim. Biophys. Acta* 1797 907–912 20079335 10.1016/j.bbabio.2010.01.005

[B327] Schlattner U. Dolder M. Wallimann T. Tokarska-Schlattner M. (2001). Mitochondrial creatine kinase and mitochondrial outer membrane porin show a direct interaction that is modulated by calcium. *J. Biol. Chem.* 276 48027–48030 11602586 10.1074/jbc.M106524200

[B328] Schneider R. Etzkorn M. Giller K. Daebel V. Eisfeld J. Zweckstetter M. (2010). The native conformation of the human VDAC1 N terminus. *Angew. Chem. Int. Ed. Engl.* 49 1882–1885 20140924 10.1002/anie.200906241

[B329] Schwarzer C. Barnikol-Watanabe S. Thinnes F. P. Hilschmann N. (2002). Voltage-dependent anion-selective channel (VDAC) interacts with the dynein light chain Tctex1 and the heat-shock protein PBP74. *Int. J. Biochem. Cell Biol.* 34 1059–1070 12009301 10.1016/s1357-2725(02)00026-2

[B330] Schwer B. Eckersdorff M. Li Y. Silva J. C. Fermin D. Kurtev M. V. (2009). Calorie restriction alters mitochondrial protein acetylation. *Aging Cell* 8 604–606 19594485 10.1111/j.1474-9726.2009.00503.x PMC2752488

[B331] Scorrano L. Oakes S. A. Opferman J. T. Cheng E. H. Sorcinelli M. D. Pozzan T. (2003). BAX and BAK regulation of endoplasmic reticulum Ca^2+^: a control point for apoptosis. *Science* 300 135–139 12624178 10.1126/science.1081208

[B332] Sentman C. L. Shutter J. R. Hockenbery D. Kanagawa O. Korsmeyer S. J. (1991). bcl-2 inhibits multiple forms of apoptosis but not negative selection in thymocytes. *Cell* 67 879–888 1835668 10.1016/0092-8674(91)90361-2

[B333] Shi H. Hudson L. G. Ding W. Wang S. Cooper K. L. Liu S. (2004). Arsenite causes DNA damage in keratinocytes via generation of hydroxyl radicals. *Chem. Res. Toxicol.* 17 871–878 15257611 10.1021/tx049939e

[B334] Shi Y. Chen J. Weng C. Chen R. Zheng Y. Chen Q. (2003a). Identification of the protein–protein contact site and interaction mode of human VDAC1 with Bcl-2 family proteins. *Biochem. Biophys. Res. Commun.* 305 989–996 12767928 10.1016/s0006-291x(03)00871-4

[B335] Shi Y. Jiang C. Chen Q. Tang H. (2003b). One-step on-column affinity refolding purification and functional analysis of recombinant human VDAC1. *Biochem. Biophys. Res. Commun.* 303 475–482 12659842 10.1016/s0006-291x(03)00359-0

[B336] Shimizu S. Ide T. Yanagida T. Tsujimoto Y. (2000a). Electrophysiological study of a novel large pore formed by Bax and the voltage-dependent anion channel that is permeable to cytochrome *c*. *J. Biol. Chem.* 275 12321–12325 10766872 10.1074/jbc.275.16.12321

[B337] Shimizu S. Konishi A. Kodama T. Tsujimoto Y. (2000b). BH4 domain of antiapoptotic Bcl-2 family members closes voltage-dependent anion channel and inhibits apoptotic mitochondrial changes and cell death. *Proc. Natl. Acad. Sci. U.S.A.* 97 3100–3105 10737788 10.1073/pnas.97.7.3100 PMC16199

[B338] Shimizu S. Shinohara Y. Tsujimoto Y. (2000c). Bax and Bcl-xL independently regulate apoptotic changes of yeast mitochondria that require VDAC but not adenine nucleotide translocator. *Oncogene* 19 4309–4318 10980606 10.1038/sj.onc.1203788

[B339] Shimizu S. Matsuoka Y. Shinohara Y. Yoneda Y. Tsujimoto Y. (2001). Essential role of voltage-dependent anion channel in various forms of apoptosis in mammalian cells. *J. Cell Biol.* 152 237–250 11266442 10.1083/jcb.152.2.237 PMC2199613

[B340] Shimizu S. Narita M. Tsujimoto Y. (1999). Bcl-2 family proteins regulate the release of apoptogenic cytochrome c by the mitochondrial channel VDAC. *Nature* 399 483–487 10365962 10.1038/20959

[B341] Shimizu S. Tsujimoto Y. (2000). Proapoptotic BH3-only Bcl-2 family members induce cytochrome *c* release, but not mitochondrial membrane potential loss, and do not directly modulate voltage-dependent anion channel activity. *Proc. Natl. Acad. Sci. U.S.A.* 97 577–582 10639121 10.1073/pnas.97.2.577 PMC15372

[B342] Shirakata Y. Koike K. (2003). Hepatitis B virus X protein induces cell death by causing loss of mitochondrial membrane potential. *J. Biol. Chem.* 278 22071–22078 12676947 10.1074/jbc.M301606200

[B343] Shoshan-Barmatz V. Arbel N. Arzoine L. (2008a). VDAC, the voltage-dependent anion channel: function, regulation & mitochondrial signaling in cell life and death. *Cell Sci.* 4 74–118

[B344] Shoshan-Barmatz V. Keinan N. Zaid H. (2008b). Uncovering the role of VDAC in the regulation of cell life and death. *J. Bioenerg. Biomembr.* 40 183–191 18651212 10.1007/s10863-008-9147-9

[B345] Shoshan-Barmatz V. Ben-Hail D. (2011). VDAC, a multi-functional mitochondrial protein as a pharmacological target. *Mitochondrion* 12 24–34 21530686 10.1016/j.mito.2011.04.001

[B346] Shoshan-Barmatz V. De Pinto V. Zweckstetter M. Raviv Z. Keinan N. Arbel N. (2010). VDAC, a multi-functional mitochondrial protein regulating cell life and death. *Mol. Aspects Med.* 31 227–285 20346371 10.1016/j.mam.2010.03.002

[B347] Shoshan-Barmatz V. Gincel D. (2003). The voltage-dependent anion channel: characterization, modulation, and role in mitochondrial function in cell life and death. *Cell Biochem. Biophys.* 39 279–292 14716081 10.1385/CBB:39:3:279

[B348] Shoshan-Barmatz V. Golan M. (2012). Mitochondrial VDAC1: function in cell life and death and a target for cancer therapy. *Curr. Med. Chem.* 19 714–735 22204343 10.2174/092986712798992110

[B349] Shoshan-Barmatz V. Hadad N. Feng W. Shafir I. Orr I. Varsanyi M. (1996). VDAC/porin is present in sarcoplasmic reticulum from skeletal muscle. *FEBS Lett.* 386 205–210 8647283 10.1016/0014-5793(96)00442-5

[B350] Shoshan-Barmatz V. Israelson A. (2005). The voltage-dependent anion channel in endoplasmic/sarcoplasmic reticulum: characterization, modulation and possible function. *J. Membr. Biol.* 204 57–66 16151701 10.1007/s00232-005-0749-4

[B351] Shoshan-Barmatz V. Israelson A. Brdiczka D. Sheu S. S. (2006). The voltage-dependent anion channel (VDAC): function in intracellular signalling, cell life and cell death. *Curr. Pharm. Des.* 12 2249–2270 16787253 10.2174/138161206777585111

[B352] Shoshan-Barmatz V. Zakar M. Rosenthal K. Abu-Hamad S. (2009). Key regions of VDAC1 functioning in apoptosis induction and regulation by hexokinase. *Biochim. Biophys. Acta* 1787 421–430 19094960 10.1016/j.bbabio.2008.11.009

[B353] Shoshan-Barmatz V. Zalk R. Gincel D. Vardi N. (2004). Subcellular localization of VDAC in mitochondria and ER in the cerebellum. *Biochim. Biophys. Acta* 1657 105–114 15238267 10.1016/j.bbabio.2004.02.009

[B354] Shulga N. Wilson-Smith R. Pastorino J. G. (2009). Hexokinase II detachment from the mitochondria potentiates cisplatin induced cytotoxicity through a caspase-2 dependent mechanism. *Cell Cycle* 8 3355–3364 19770592 10.4161/cc.8.20.9853 PMC2829766

[B355] Sileikyte J. Petronilli V. Zulian A. Dabbeni-Sala F. Tognon G. Nikolov P. (2011). Regulation of the inner membrane mitochondrial permeability transition by the outer membrane translocator protein (peripheral benzodiazepine receptor). *J. Biol. Chem.* 286 1046–1053 21062740 10.1074/jbc.M110.172486 PMC3020711

[B356] Simamura E. Hirai K. Shimada H. Koyama J. Niwa Y. Shimizu S. (2006). Furanonaphthoquinones cause apoptosis of cancer cells by inducing the production of reactive oxygen species by the mitochondrial voltage-dependent anion channel. *Cancer Biol. Ther.* 5 1523–1529 17012850 10.4161/cbt.5.11.3302

[B357] Simamura E. Shimada H. Ishigaki Y. Hatta T. Higashi N. Hirai K. (2008). Bioreductive activation of quinone antitumor drugs by mitochondrial voltage-dependent anion channel 1. *Anat. Sci. Int.* 83 261–266 19159355 10.1111/j.1447-073X.2008.00241.x

[B358] Siskind L. J. Colombini M. (2000). The lipids C2- and C16-ceramide form large stable channels. Implications for apoptosis. *J. Biol. Chem.* 275 38640–38644 11027675 10.1074/jbc.C000587200 PMC2094390

[B359] Siskind L. J. Kolesnick R. N. Colombini M. (2006). Ceramide forms channels in mitochondrial outer membranes at physiologically relevant concentrations. *Mitochondrion* 6 118–125 16713754 10.1016/j.mito.2006.03.002 PMC2246045

[B360] Sokolove P. M. (1994). Interactions of adriamycin aglycones with mitochondria may mediate adriamycin cardiotoxicity. *Int. J. Biochem.* 26 1341–1350 7890113 10.1016/0020-711x(94)90176-7

[B361] Song J. Midson C. Blachly-Dyson E. Forte M. Colombini M. (1998). The topology of VDAC as probed by biotin modification. *J. Biol. Chem.* 273 24406–24413 9733730 10.1074/jbc.273.38.24406

[B362] Stachowiak O. Schlattner U. Dolder M. Wallimann T. (1998). Oligomeric state and membrane binding behaviour of creatine kinase isoenzymes: implications for cellular function and mitochondrial structure. *Mol. Cell. Biochem.* 184 141–151 9746318

[B363] Stanley S. Dias J. A. D’Arcangelis D. Mannella C. A. (1995). Peptide-specific antibodies as probes of the topography of the voltage-gated channel in the mitochondrial outer membrane of Neurospora crassa. *J. Biol. Chem.* 270 16694–16700 7542652 10.1074/jbc.270.28.16694

[B364] Stiban J. Caputo L. Colombini M. (2008). Ceramide synthesis in the endoplasmic reticulum can permeabilize mitochondria to proapoptotic proteins. *J. Lipid Res.* 49 625–634 18073406 10.1194/jlr.M700480-JLR200

[B365] Su J. C. Lin K. L. Chien C. M. Tseng C. H. Chen Y. L. Chang L. S. (2010). Furano-1,2-naphthoquinone inhibits EGFR signaling associated with G2/M cell cycle arrest and apoptosis in A549 cells. *Cell Biochem. Funct.* 28 695–705 21104938 10.1002/cbf.1710

[B366] Sugiyama T. Shimizu S. Matsuoka Y. Yoneda Y. Tsujimoto Y. (2002). Activation of mitochondrial voltage-dependent anion channel by apro-apoptotic BH3-only protein Bim. *Oncogene* 21 4944–4956 12118373 10.1038/sj.onc.1205621

[B367] Sui Y. Potula R. Dhillon N. Pinson D. Li S. Nath A. (2004). Neuronal apoptosis is mediated by CXCL10 overexpression in simian human immunodeficiency virus encephalitis. *Am. J. Pathol.* 164 1557–1566 15111302 10.1016/S0002-9440(10)63714-5 PMC1615658

[B368] Summers W. A. Court D. A. (2010). Origami in outer membrane mimetics: correlating the first detailed images of refolded VDAC with over 20 years of biochemical data. *Biochem. Cell Biol.* 88 425–438 20555384 10.1139/o09-115

[B369] Sun J. Liao J. K. (2002). Functional interaction of endothelial nitric oxide synthase with a voltage-dependent anion channel. *Proc. Natl. Acad. Sci. U.S.A.* 99 13108–13113 12228731 10.1073/pnas.202260999 PMC130594

[B370] Sun L. Shukair S. Naik T. J. Moazed F. Ardehali H. (2008). Glucose phosphorylation and mitochondrial binding are required for the protective effects of hexokinases I and II. *Mol. Cell. Biol.* 28 1007–1017 18039843 10.1128/MCB.00224-07 PMC2223386

[B371] Sundararajan R. Cuconati A. Nelson D. White E. (2001). Tumor necrosis factor-alpha induces Bax-Bak interaction and apoptosis, which is inhibited by adenovirus E1B 19K. *J. Biol. Chem.* 276 45120–45127 11571294 10.1074/jbc.M106386200

[B372] Szabadkai G. Bianchi K. Várnai P. De Stefani D. Wieckowski M. R. Cavagna D. (2006). Chaperone-mediated coupling of endoplasmic reticulum and mitochondrial Ca^2+^ channels. *J. Cell Biol.* 175 901–911 17178908 10.1083/jcb.200608073 PMC2064700

[B373] Tajeddine N. Galluzzi L. Kepp O. Hangen E. Morselli E. Senovilla L. (2008). Hierarchical involvement of Bak, VDAC1 and Bax in cisplatin-induced cell death. *Oncogene* 10 4221–4232 18362892 10.1038/onc.2008.63

[B374] Tan W. Colombini M. (2007). VDAC closure increases calcium ion flux. *Biochim. Biophys. Acta* 1768 2510–2515 17617374 10.1016/j.bbamem.2007.06.002 PMC2220155

[B375] Tan W. Loke Y. H. Stein C. A. Miller P. Colombini M. (2007). Phosphorothioate oligonucleotides block the VDAC channel. *Biophys. J.* 93 1184–1191 17483171 10.1529/biophysj.107.105379 PMC1929033

[B376] Tang H. L. Le A. H. Lung H. L. (2006). The increase in mitochondrial association with actin precedes Bax translocation in apoptosis. *Biochem. J.* 396 1–5 16536728 10.1042/BJ20060241 PMC1449994

[B377] Tarze A. Deniaud A. Le Bras M. Maillier E. Molle D. Larochette N. (2007). GAPDH, a novel regulator of the pro-apoptotic mitochondrial membrane permeabilization. *Oncogene* 26 2606–2620 17072346 10.1038/sj.onc.1210074

[B378] Teijido O. Ujwal R. Hillerdal C. O. Kullman L. Rostovtseva T. K. Abramson J. (2012). Affixing the N-terminal alpha helix of the voltage dependent anion channel to the channel’s wall does not prevent its voltage gating. *J. Biol. Chem.* 287 11437–11445 22275367 10.1074/jbc.M111.314229 PMC3322836

[B379] Thinnes F. P. (2005). Does fluoxetine (Prozak) block mitochondrial permeability transition by blocking VDAC as part of permeability transition pores? *Mol. Genet. Metab.* 84 378 10.1016/j.ymgme.2004.12.008 15781203

[B380] Thinnes F. P. (2009). Human type-1 VDAC, a cisplatin target involved in either apoptotic pathway. *Mol. Genet. Metab.* 97 163 10.1016/j.ymgme.2009.01.014 19251445

[B381] Thinnes F. P. Götz H. Kayser H. Benz R. Schmidt W. E. Kratzin H. D. (1989). Identification of human porins. I. Purification of a porin from human B-lymphocytes (Porin 31HL) and the topochemical proof of its expression on the plasmalemma of the progenitor cell. *Biol. Chem. Hoppe Seyler* 370 1253–1264 2559744

[B382] Tikunov A. Johnson C. B. Pediaditakis P. Markevich N. Macdonald J. M. Lemasters J. J. (2010). Closure of VDAC causes oxidative stress and accelerates the Ca(2+)-induced mitochondrial permeability transition in rat liver mitochondria. *Arch. Biochem. Biophys.* 495 174–181 20097153 10.1016/j.abb.2010.01.008 PMC2855314

[B383] Tinhofer I. Bernhard D. Senfter M. Anether G. Loeffler M. Kroemer G. (2001). Resveratrol, a tumor-suppressive compound from grapes, induces apoptosis via a novel mitochondrial pathway controlled by Bcl-2. *FASEB J.* 15 1613–1615 11427503 10.1096/fj.00-0675fje

[B384] Tomasello F. Messina A. Lartigue L. Schembri L. Medina C. Reina S. (2009). Outer membrane VDAC1 controls permeability transition of the inner mitochondrial membrane in cellulo during stress-induced apoptosis. *Cell Res.* 19 1363–1376 19668262 10.1038/cr.2009.98

[B385] Trachootham D. Zhou Y. Zhang H. Demizu Y. Chen Z. Pelicano H. (2006). Selective killing of oncogenically transformed cells through a ROS-mediated mechanism by beta-phenylethyl isothiocyanate. *Cancer Cell* 10 241–252 16959615 10.1016/j.ccr.2006.08.009

[B386] Triphan X. Menzel V. A. Petrunkina A. M. Cassará M. C. Wemheuer W. Hinsch K. D. (2008). Localisation and function of voltage-dependent anion channels (VDAC) in bovine spermatozoa. *Pflugers Arch.* 455 677–686 17647012 10.1007/s00424-007-0316-1

[B387] Tsujimoto Y. (2003). Cell death regulation by the Bcl-2 protein family in the mitochondria. *J. Cell Physiol.* 195 158–167 12652643 10.1002/jcp.10254

[B388] Tsujimoto Y. Finger L. R. Yunis J. Nowell P. C. Croce C. M. (1984). Cloning of the chromosome breakpoint of neoplastic B cells with the t(14;18) chromosome translocation. *Science* 226 1097–1099 6093263 10.1126/science.6093263

[B389] Tsujimoto Y. Shimizu S. (2002). The voltage-dependent anion channel: an essential player in apoptosis. *Biochimie* 84 187–193 12022949 10.1016/s0300-9084(02)01370-6

[B390] Tsujimoto Y. Shimizu S. (2007). Role of the mitochondrial membrane permeability transition in cell death. *Apoptosis* 12 835–840 17136322 10.1007/s10495-006-0525-7

[B391] Ujwal R. Cascio D. Chaptal V. Ping P. Abramson J. (2009). Crystal packing analysis of murine VDAC1 crystals in a lipidic environment reveals novel insights on oligomerization and orientation. *Channels (Austin)* 3 167–170 19574737 10.4161/chan.3.3.9196 PMC3719987

[B392] Ujwal R. Cascio D. Colletier J. P. Faham S. Zhang J. Toro L. (2008). The crystal structure of mouse VDAC1 at 2.3 A resolution reveals mechanistic insights into metabolite gating. *Proc. Natl. Acad. Sci. U.S.A.* 105 17742–17747 18988731 10.1073/pnas.0809634105 PMC2584669

[B393] Vander Heiden M. G. Chandel N. S. Li X. X. Schumacker P. T. Colombini M. Thompson C. B. (2000). Outer mitochondrial membrane permeability can regulate coupled respiration and cell survival. *Proc. Natl. Acad. Sci. U.S.A.* 97 4666–4671 10781072 10.1073/pnas.090082297 PMC18290

[B394] Vander Heiden M. G. Li X. X. Gottleib E. Hill R. B. Thompson C. B. Colombini M. (2001). Bcl-xL promotes the open configuration of the voltage-dependent anion channel and metabolite passage through the outer mitochondrial membrane. *J. Biol. Chem.* 276 19414–19419 11259441 10.1074/jbc.M101590200

[B395] Veenman L. Leschiner S. Spanier I. Weisinger G. Weizman A. Gavish M. (2002). PK 11195 attenuates kainic acid-induced seizures and alterations in peripheral-type benzodiazepine receptor (PBR) protein components in the rat brain. *J. Neurochem.* 80 917–927 11948256 10.1046/j.0022-3042.2002.00769.x

[B396] Veenman L. Levin E. Weisinger G. Leschiner S. Spanier I. Snyder S. H. (2004). Peripheral-type benzodiazepine receptor density and in vitro tumorigenicity of glioma cell lines. *Biochem. Pharmacol.* 68 689–698 15276076 10.1016/j.bcp.2004.05.011

[B397] Veenman L. Papadopoulos V. Gavish M. (2007). Channel-like functions of the 18-kDa translocator protein (TSPO): regulation of apoptosis and steroidogenesis as part of the host-defense response. *Curr. Pharm. Des.* 13 2385–2405 17692008 10.2174/138161207781368710

[B398] Veenman L. Shandalov Y. Gavish M. (2008). VDAC activation by the 18 kDa translocator protein (TSPO), implications for apoptosis. *J. Bioenerg. Biomembr.* 40 199–205 18670869 10.1007/s10863-008-9142-1

[B399] Verkhratsky A. Petersen O. H. (2002). The endoplasmic reticulum as an integrating signalling organelle: from neuronal signalling to neuronal death. *Eur. J. Pharmacol.* 447 141–154 12151006 10.1016/s0014-2999(02)01838-1

[B400] Verrier F. Mignotte B. Jan G. Brenner C. (2003). Study of PTPC composition during apoptosis for identification of viral protein target. *Ann. N. Y. Acad. Sci.* 1010 126–142 15033708 10.1196/annals.1299.022

[B401] Vieira H. L. Haouzi D. El Hamel C. Jacotot E. Belzacq A. S. Brenner C. (2000). Permeabilization of the mitochondrial inner membrane during apoptosis: impact of the adenine nucleotide translocator. *Cell Death Differ.* 7 1146–1154 11175251 10.1038/sj.cdd.4400778

[B402] Voehringer D. W. Hirschberg D. L. Xiao J. Lu Q. Roederer M. Lock C. B. (2000). Gene microarray identification of redox and mitochondrial elements that control resistance or sensitivity to apoptosis. *Proc. Natl. Acad. Sci. U.S.A.* 97 2680–2685 10716996 10.1073/pnas.97.6.2680 PMC15989

[B403] Voulgaridou G. P. Anestopoulos I. Franco R. Panayiotidis M. I. Pappa A. (2011). DNA damage induced by endogenous aldehydes: current state of knowledge. *Mutat. Res.* 711 13–27 21419140 10.1016/j.mrfmmm.2011.03.006

[B404] Vyssokikh M. Y. Brdiczka D. (2003). The function of complexes between the outer mitochondrial membrane pore (VDAC) and the adenine nucleotide translocase in regulation of energy metabolism and apoptosis. *Acta Biochim. Pol.* 50 389–404 12833165

[B405] Wan K. F. Chan S. L. Sukumaran S. K. Lee M. C. Yu V. C. (2008). Chelerythrine induces apoptosis through a Bax/Bak-independent mitochondrial mechanism. *J. Biol. Chem.* 283 8423–8433 18230621 10.1074/jbc.M707687200 PMC2417179

[B406] Wang B. Malik R. Nigg E. A Körner R. (2008). Evaluation of the low-specificity protease elastase for large-scale phosphoproteome analysis. *Anal. Chem.* 80 9526–9533 19007248 10.1021/ac801708p

[B407] Wang Y. Zhang X. H. Wang H. L. (2011). Involvement of BMPR2 in the protective effect of fluoxetine against monocrotaline-induced endothelial apoptosis in rats. *Can. J. Physiol. Pharmacol.* 89 345–354 21619414 10.1139/y11-024

[B408] Watabe M. Machida K. Osada H. (2000). MT-21 is a synthetic apoptosis inducer that directly induces cytochrome c release from mitochondria. *Cancer Res.* 60 5214–5222 11016650

[B409] Weeber E. J. Levy M. Sampson M. J. Anflous K. Armstrong D. L. Brown S. E. (2002). The role of mitochondrial porins and the permeability transition pore in learning and synaptic plasticity. *J. Biol. Chem.* 277 18891–18897 11907043 10.1074/jbc.M201649200

[B410] Wei M. C. Lindsten T. Mootha V. K. Weiler S. Gross A. Ashiya M. (2000). tBID, a membrane-targeted death ligand, oligomerizes BAK to release cytochrome *c*. *Genes Dev.* 14 2060–2071 10950869 PMC316859

[B411] Wei M. C. Zong W. X. Cheng E. H. Lindsten T. Panoutsakopoulou V. Ross A. J. (2001). Proapoptotic BAX and BAK: a requisite gateway to mitochondrial dysfunction and death. *Science* 292 727–730 11326099 10.1126/science.1059108 PMC3049805

[B412] Whittington D. A. Waheed A. Ulmasov B. Shah G. N. Grubb J. H. Sly W. S. (2001). Crystal structure of the dimeric extracellular domain of human carbonic anhydrase XII, a bitopic membrane protein overexpressed in certain cancer tumor cells. *Proc. Natl. Acad. Sci. U.S.A.* 98 9545–9550 11493685 10.1073/pnas.161301298 PMC55489

[B413] Wigdal S. S. Kirkland R. A. Franklin J. L. Haak-Frendscho M. (2002). Cytochrome *c* release precedes mitochondrial membrane potential loss in cerebellar granule neuron apoptosis: lack of mitochondrial swelling. *J. Neurochem.* 82 1029–1038 12358750 10.1046/j.1471-4159.2002.01049.x

[B414] Wong D. T. Bymaster F. P. Engleman E. A. (1995). Prozac (fluoxetine, Lilly 110140), the first selective serotonin uptake inhibitor and an antidepressant drug: twenty years since its first publication. *Life Sci.* 57 411–441 7623609 10.1016/0024-3205(95)00209-o

[B415] Wright S. C. Zhong J. Larrick J. W. (1994). Inhibition of apoptosis as a mechanism of tumor promotion. *FASEB J.* 8 654–660 8005393 10.1096/fasebj.8.9.8005393

[B416] Wu S. Sampson M. J. Decker W. K. Craigen W. J. (1999). Each mammalian mitochondrial outer membrane porin protein is dispensable: effects on cellular respiration. *Biochim. Biophys. Acta* 1452 68–78 10525161 10.1016/s0167-4889(99)00120-2

[B417] Xie G. Wilson J. E. (1990). Tetrameric structure of mitochondrially bound rat brain hexokinase: a crosslinking study. *Arch. Biochem. Biophys.* 276 285–293 2297228 10.1016/0003-9861(90)90040-6

[B418] Xie Q. Wondergem R. Shen Y. Cavey G. Ke J. Thompson R. (2011). Benzoquinone ansamycin 17AAG binds to mitochondrial voltage-dependent anion channel and inhibits cell invasion. *Proc. Natl. Acad. Sci. U.S.A.* 108 4105–4110 21368131 10.1073/pnas.1015181108 PMC3053964

[B419] Xu X. Decker W. Sampson M. J. Craigen W. J. Colombini M. (1999). Mouse VDAC isoforms expressed in yeast: channel properties and their roles in mitochondrial outer membrane permeability. *J. Membr. Biol.* 170 89–102 10430654 10.1007/s002329900540

[B420] Xu X. Forbes J. G. Colombini M. (2001). Actin modulates the gating of *Neurospora crassa* VDAC. *J. Membr. Biol.* 180 73–81 11284205 10.1007/s002320010060

[B421] Yagoda N. von Rechenberg M. Zaganjor E. Bauer A. J. Yang W. S. Fridman D. J. (2007). RAS-RAF-MEK-dependent oxidative cell death involving voltage-dependent anion channels. *Nature* 447 864–868 17568748 10.1038/nature05859 PMC3047570

[B422] Yamamoto T. Yamada A. Watanabe M. Yoshimura Y. Yamazaki N. Yoshimura Y. (2006). VDAC1, having a shorter N-terminus than VDAC2 but showing the same migration in an SDS-polyacrylamide gel, is the predominant form expressed in mitochondria of various tissues. *J. Proteome Res.* 5 3336–3344 17137335 10.1021/pr060291w

[B423] Yang L. Vaitheesvaran B. Hartil K. Robinson A. J. Hoopmann M. R. Eng J. K. (2011). The fasted/fed mouse metabolic acetylome: N6-acetylation differences suggest acetylation coordinates organ-specific fuel switching. *J. Proteome Res.* 10 4134–4149 21728379 10.1021/pr200313x PMC3204869

[B424] Yang W. S. Stockwell B. R. (2008). Synthetic lethal screening identifies compounds activating iron-dependent, nonapoptotic cell death in oncogenic-RAS-harboring cancer cells. *Chem. Biol.* 15 234–245 18355723 10.1016/j.chembiol.2008.02.010 PMC2683762

[B425] Yang Z. Schumaker L. M. Egorin M. J. Zuhowski E. G. Guo Z. Cullen K. J. (2006). Cisplatin preferentially binds mitochondrial DNA and voltage-dependent anion channel protein in the mitochondrial membrane of head and neck squamous cell carcinoma: possible role in apoptosis. *Clin. Cancer Res.* 12 5817–5825 17020989 10.1158/1078-0432.CCR-06-1037

[B426] Yehezkel G. Abu-Hamad S. Shoshan-Barmatz V. (2007). An N-terminal nucleotide-binding site in VDAC1: involvement in regulating mitochondrial function. *J. Cell. Physiol.* 212 551–561 17503466 10.1002/jcp.21048

[B427] Yehezkel G. Hadad N. Zaid H. Sivan S. Shoshan-Barmatz V. (2006). Nucleotide-binding sites in the voltage-dependent anion channel: characterization and localization. *J. Biol. Chem.* 281 5938–5946 16354668 10.1074/jbc.M510104200

[B428] Yin X. M. (2000). Signal transduction mediated by Bid, a pro-death Bcl-2 family proteins, connects the death receptor and mitochondria apoptosis pathways. *Cell Res.* 10 161–167 11032168 10.1038/sj.cr.7290045

[B429] Yoo N. J. Park S. W. Lee S. H. (2011). A frameshift mutation of the pro-apoptotic VDAC1 gene in cancers with microsatellite instability. *Gut Liver* 5 548–549 22195259 10.5009/gnl.2011.5.4.548 PMC3240804

[B430] Youle R. J. Strasser A. (2008). The BCL-2 protein family: opposing activities that mediate cell death. *Nat. Rev. Mol. Cell Biol.* 9 47–59 18097445 10.1038/nrm2308

[B431] Yu W. H. Forte M. (1996). Is there VDAC in cell compartments other than the mitochondria? *J. Bioenerg. Biomembr.* 28 93–100 9132422 10.1007/BF02110638

[B432] Yuan H. Williams S. D. Adachi S. Oltersdorf T. Gottlieb R. A. (2003). Cytochrome *c* dissociation and release from mitochondria by truncated Bid and ceramide. *Mitochondrion* 2 237–244 16120324 10.1016/S1567-7249(02)00106-X

[B433] Yuan S. Galluzzi L. Kepp O. Hangen E. Morselli E. Senovilla L. (2008). Voltage-dependent anion channel 1 is involved in endostatin-induced endothelial cell apoptosis. *FASEB J.* 22 2809–2820 18381814 10.1096/fj.08-107417

[B434] Zachariae U. Schneider R. Briones R. Gattin Z. Demers J. P. Giller K. (2012). beta-Barrel mobility underlies closure of the voltage-dependent anion channel. *Structure* 20 1540–1549 22841291 10.1016/j.str.2012.06.015 PMC5650048

[B435] Zaid H. Abu-Hamad S. Israelson A. Nathan I. Shoshan-Barmatz V. (2005). The voltage-dependent anion channel-1 modulates apoptotic cell death. *Cell Death Differ.* 12 751–760 15818409 10.1038/sj.cdd.4401599

[B436] Zalk R. Israelson A. Garty E. S. Azoulay-Zohar H. Shoshan-Barmatz V. (2005). Oligomeric states of the voltage-dependent anion channel and cytochrome c release from mitochondria. *Biochem. J.* 386(Pt 1) 73–83 15456403 10.1042/BJ20041356 PMC1134768

[B437] Zamarin D. García-Sastre A. Xiao X. Wang R. Palese P. (2005). Influenza virus PB1-F2 protein induces cell death through mitochondrial ANT3 and VDAC1. *PLoS Pathog.* 1 e4 10.1371/journal.ppat.0010004 PMC1238739 16201016

[B438] Zamzami N. El Hamel C. Maisse C. Brenner C. Muñoz-Pinedo C. Belzacq A. S. (2000). Bid acts on the permeability transition pore complex to induce apoptosis. *Oncogene* 19 6342–6350 11175349 10.1038/sj.onc.1204030

[B439] Zamzami N. Marzo I. Susin S. A. Brenner C. Larochette N. Marchetti P. (1998). The thiol crosslinking agent diamide overcomes the apoptosis-inhibitory effect of Bcl-2 by enforcing mitochondrial permeability transition. *Oncogene* 16 1055–1063 9519879 10.1038/sj.onc.1201864

[B440] Zeth K. Meins T. Vonrhein C. (2008). Approaching the structure of human VDAC1, a key molecule in mitochondrial cross-talk. *J. Bioenerg. Biomembr.* 40 127–132 18690523 10.1007/s10863-008-9144-z

[B441] Zhang T. Tang S. S. Jin X. Liu F. Y. Zhang C. M. Zhao W. X. (2011). c-Myc influences olaquindox-induced apoptosis in human hepatoma G2 cells. *Mol. Cell. Biochem.* 354 253–261 21598074 10.1007/s11010-011-0825-2

[B442] Zhao S. Xu W. Jiang W. Yu W. Lin Y. Zhang T. (2010). Regulation of cellular metabolism by protein lysine acetylation. *Science* 327 1000–1004 20167786 10.1126/science.1179689 PMC3232675

[B443] Zheng Y. Shi Y. Tian C. Jiang C. Jin H. Chen J. (2004). Essential role of the voltage-dependent anion channel (VDAC) in mitochondrial permeability transition pore opening and cytochrome c release induced by arsenic trioxide. *Oncogene* 23 1239–1247 14647451 10.1038/sj.onc.1207205 PMC2913247

